# Associations of cardiometabolic burden with cardiovascular disease–death transitions in metabolic dysfunction-associated steatotic liver disease: a prospective cohort study using multistate analysis

**DOI:** 10.3389/fnut.2026.1928761

**Published:** 2026-09-18

**Authors:** Chuanyong Cui, Yi Zhao, Guangji Zhang, Zhongyan Du, Wenke Cheng

**Affiliations:** 1 Department of Cardiology, The First Affiliated Hospital of Bengbu Medical University, Bengbu, China; 2 Zhejiang Key Laboratory of Blood-Stasis-Toxin Syndrome, Zhejiang Chinese Medical University, Hangzhou, China; 3 School of Basic Medical Sciences, Zhejiang Chinese Medical University, Hangzhou, China; 4 Zhejiang Engineering Research Center for "Preventive Treatment" Smart Health of Traditional Chinese Medicine, Zhejiang Chinese Medical University, Hangzhou, China; 5 School of Life Science, Anhui Provincial Key Laboratory of Tumor Evolution and Intelligent Diagnosis and Treatment, Anhui Province Key Laboratory of Immunology in Chronic Diseases, Bengbu Medical University, Bengbu, China

**Keywords:** cardiometabolic risk factor, cardiovascular disease, cohort study, death, metabolic dysfunction-associated steatotic liver disease

## Abstract

**Aim:**

To examine the associations of cardiometabolic burden with transitions from CVD-free status to incident CVD, death without prior CVD, and death after CVD among individuals with MASLD, and to further characterize non-linear patterns, temporal variations, and potential contributing factors related to these associations.

**Methods:**

This prospective cohort analysis included 72,922 UK adults with MASLD. Cardiometabolic burden was primarily assessed by the number of cardiometabolic risk factors, with an internally derived weighted score evaluated as a complementary secondary measure. Traditional Cox models estimated overall associations with incident CVD and death, whereas multistate models quantified transition-specific associations. Penalised splines assessed non-linearity, piecewise Cox models evaluated time-stratified associations, and mediation analyses examined inflammatory and lifestyle-related factors.

**Results:**

Over a median follow-up of 15.9 years, 15,352 participants developed CVD, 4,273 died without prior CVD, and 4,228 died after incident CVD. Each additional cardiometabolic risk factor was associated with higher risks of the baseline-to-CVD, baseline-to-death, and CVD-to-death transitions (hazard ratio [HR] 1.17, 95% confidence interval [CI] 1.15–1.19; HR 1.09, 95% CI 1.06–1.13; and HR 1.13, 95% CI 1.10–1.17, respectively). Corresponding estimates per 1-standard deviation increase in the weighted score were HR 1.23 (95% CI 1.20–1.25), HR 1.12 (95% CI 1.08–1.16), and HR 1.19 (95% CI 1.15–1.23), respectively. Associations with incident CVD were stronger during early follow-up, whereas death-related associations became more apparent over longer follow-up. Significant age interactions were observed, with stronger associations among participants aged <60 years (P for interaction <0.05). In mediation analyses, estimated indirect proportions were generally <3% for circulating inflammatory markers and were larger for smoking and drinking status.

**Conclusion:**

Among individuals with MASLD, greater cardiometabolic burden was associated with all disease-state transitions, most strongly with incident CVD. Death-related associations became more apparent over longer follow-up, supporting sustained cardiometabolic surveillance. Associations were more pronounced in younger participants, suggesting that individuals under 60 years with high cardiometabolic burden may be *a priori*ty subgroup for earlier identification and transition-specific risk assessment.

## Introduction

Metabolic dysfunction-associated steatotic liver disease (MASLD), formerly termed non-alcoholic fatty liver disease, is defined by hepatic steatosis in the presence of at least one cardiometabolic risk factor (CMRF), in the absence of excessive alcohol consumption or other established causes of liver disease (1, 2). By explicitly linking hepatic steatosis to systemic metabolic dysfunction, the MASLD framework reframes fatty liver disease as a multisystem cardiometabolic disorder rather than an isolated hepatic condition. MASLD affects approximately one-third of adults worldwide and has become a major public health challenge (3). Although liver-related complications remain important, cardiovascular disease (CVD) is a leading extrahepatic complication and a major determinant of long-term prognosis in individuals with MASLD. Increasing evidence indicates that adverse outcomes in MASLD are closely related not only to hepatic steatosis itself, but also to the broader cardiometabolic abnormalities that accompany it (4, 5).

Cardiometabolic risk factors have been extensively studied in the general population and in the context of metabolic syndrome (6, 7). However, examining cardiometabolic burden within MASLD has distinct clinical relevance. MASLD represents hepatic steatosis occurring in the context of systemic metabolic dysfunction, yet individuals with MASLD may exhibit substantial variation in the extent of cardiometabolic abnormalities despite a shared diagnosis of MASLD. Because all individuals with MASLD have at least one CMRF by definition, the key clinical question is not simply whether metabolic abnormalities are present, but whether the degree of cardiometabolic burden further stratifies disease progression within an already metabolically vulnerable population. Prior studies have shown that both the number of CMRFs and their longitudinal changes are associated with incident CVD and mortality in MASLD (8–10). However, these studies have largely relied on endpoint-specific analyses that evaluate CVD and death separately. Even competing-risk approaches do not fully capture the sequential evolution from CVD-free status to incident CVD, death before CVD, and death after CVD. A multistate framework may therefore provide a more comprehensive assessment of how cardiometabolic burden shapes disease progression in MASLD.

This distinction is clinically important because individuals with MASLD may follow different life-course disease trajectories: remaining free of CVD, developing CVD before death, or dying without recorded CVD. CVD is therefore not only an endpoint but also an intermediate state that may alter subsequent mortality risk. Conventional endpoint-based analyses cannot separate these trajectories or determine whether cardiometabolic burden is primarily associated with CVD onset, direct death without prior CVD, or death after CVD. A multistate framework addresses this gap by estimating transition-specific associations across successive disease states and may provide a more clinically informative characterisation of the dynamic disease course of MASLD.

Another unresolved issue is how cardiometabolic burden should be summarised. A simple count of CMRFs is intuitive and clinically accessible, but it assumes that all components contribute equally to risk. Because individual metabolic abnormalities may differ in their prognostic relevance, a weighted summary score may provide a complementary approach by capturing component-level heterogeneity (11). However, internally derived scores require validation to mitigate concerns regarding overfitting. Accordingly, we used two complementary approaches to characterise cardiometabolic burden: a count-based measure reflecting the accumulation of CMRFs and an internally derived weighted score reflecting component-level heterogeneity, which was additionally evaluated through split-sample internal validation. Against this background, we used data from the UK Biobank to examine the associations of cardiometabolic burden with disease-state transitions from CVD-free status to incident CVD, death without prior CVD, and death after CVD among individuals with MASLD.

## Methods

### Study population

This analysis was conducted using data from more than 502,000 participants enrolled in the UK Biobank between 2006 and 2010 (12). Baseline data were collected through touchscreen questionnaires, nurse-led interviews, physical examinations, and biological sample collection. Linked primary care, hospital admission, and registry data were used for outcome ascertainment. Details of the study design, recruitment procedures, and data collection have been described previously (13). The UK Biobank received ethical approval from the North West Multi-centre Research Ethics Committee (11/NW/0382), and all participants provided written informed consent. This study was registered with the Medical Research Registration Information System (registration no. MR-34-26-058122).

Participants with missing FLI data (*n* = 35,526), missing alcohol consumption data (*n* = 52,822), excessive alcohol consumption according to MASLD criteria (*n* = 127,662) (Supplementary Table S1), or a history of alcohol-related or other non-metabolic chronic liver disease (*n* = 3,041) (Supplementary Tables S2, S3) were first excluded to define the population eligible for MASLD assessment. Among the remaining participants, 102,572 met the diagnostic criteria for MASLD at baseline. We then excluded individuals with prevalent CVD, defined as a history of coronary artery disease (CAD), stroke, atrial fibrillation (AF), or heart failure (HF) (Supplementary Table S4), as well as those with cancer, pregnant participants, and individuals with incomplete information required to fully define cardiometabolic abnormalities. Participants with cancer at baseline were excluded in the primary analysis because pre-existing malignancy may substantially alter subsequent mortality risk and disease trajectories. This criterion was relaxed in sensitivity analyses to assess the robustness of the findings. Finally, 72,922 participants were included in the analytic cohort. The participant selection process is shown in Supplementary Figure S1.

### Assessment of MASLD and cardiometabolic burden

MASLD was defined in accordance with the 2023 multi-society Delphi consensus nomenclature as hepatic steatosis in the presence of at least one CMRF (2). Excessive alcohol consumption was defined as ≥210 g/week in men and ≥140 g/week in women. Consistent with previous UK Biobank studies, hepatic steatosis was defined using the fatty liver index (FLI), with FLI ≥ 60 indicating steatosis (14, 15). The FLI incorporates body mass index (BMI), waist circumference (WC), triglycerides (TG), and gamma-glutamyltransferase, and has been validated against imaging-based measures of hepatic steatosis in previous studies (16) (Supplementary Table S5). FLI was used only to identify participants with hepatic steatosis, whereas cardiometabolic burden was assessed separately to characterize the extent of metabolic abnormalities among individuals with MASLD.

CMRFs were defined as five abnormalities: (i) overweight/obesity or central adiposity, defined as body mass index ≥25 kg/m^2^ or waist circumference >94 cm in men and >80 cm in women; (ii) dysglycaemia, defined as haemoglobin A1c ≥ 39 mmol/mol, type 2 diabetes, or treatment for type 2 diabetes; (iii) elevated blood pressure, defined as blood pressure ≥130/85 mmHg or use of antihypertensive therapy; (iv) hypertriglyceridaemia, defined as triglycerides ≥1.70 mmol/L or use of lipid-lowering therapy; and (v) low high-density lipoprotein cholesterol, defined as ≤1.0 mmol/L in men and ≤1.3 mmol/L in women, or use of lipid-lowering therapy (Supplementary Table S6) (2).

Cardiometabolic burden was assessed in two complementary ways. Count-based cardiometabolic burden was calculated as the total number of CMRFs present and ranged from 1 to 5, as MASLD requires at least one CMRF by definition. As a supplementary measure of cardiometabolic burden, a weighted score was derived using coefficients from a multivariable Cox proportional hazards model for incident CVD. The model simultaneously included the five binary CMRF components and prespecified covariates. The weighted score was calculated as the sum of each CMRF multiplied by its corresponding regression coefficient (β) and standardised to a mean of 0 and standard deviation of 1 for analysis. Component-specific coefficients are provided in Supplementary Table S7 and Supplementary Figure S2. This internally derived weighted score was used as a supplementary exposure measure rather than a clinical prediction model.

### Assessment of CVD and death

Incident CVD was ascertained from the UK Biobank First Occurrences CVD section (Category 1712), which integrates primary care records, hospital inpatient records, death registry data, and self-reported diagnoses mapped to harmonised ICD-10 phenotypes. The primary outcome was composite CVD, defined as the first occurrence of CAD (ICD-10 codes I20–I25), stroke (I60–I64), AF (I48), or HF (I50), with the CVD event date defined as the earliest recorded date among these four components (Supplementary Table S4). Secondary outcomes included the individual CVD components: CAD, stroke, AF, and HF. Dates of death were obtained through linkage to national death registries, including NHS Digital for England and Wales and the NHS Central Register for Scotland (13). Participants without a recorded death date were censored at the end of available follow-up, defined by loss to follow-up or administrative censoring, whichever occurred first.

To ensure consistent ascertainment of both CVD and death outcomes, all analyses were administratively censored on 2 November 2024. Follow-up began at baseline and ended at the first occurrence of the relevant outcome event, loss to follow-up, or administrative censoring, whichever occurred first.

### Covariates

Covariates were prespecified a priori based on established epidemiological evidence regarding potential confounding factors for the associations between cardiometabolic burden and cardiovascular outcomes, previous MASLD-related studies, and data availability in the UK Biobank (10, 17). These included age, sex, self-reported ethnicity, smoking status, alcohol intake, physical activity, socioeconomic deprivation, and liver biochemistry markers. Self-reported ethnicity was categorised as White, Black, Asian, or other ethnic groups. Due to limited numbers within individual minority categories, smaller groups were combined into the “other” category to ensure sufficient statistical precision for covariate adjustment. Smoking status was categorised as never, previous, current, or unknown. Alcohol intake was estimated as weekly ethanol consumption in grams from beverage-specific intake using standard UK unit equivalents, with 1 unit corresponding to 8 g of ethanol (18) (Supplementary Table S1).

Physical activity was assessed using the International Physical Activity Questionnaire short form and categorised as low, moderate, or high; missing values were retained as a separate category. Socioeconomic deprivation was assessed using the Townsend Deprivation Index (TDI), with higher values indicating greater deprivation. Liver biochemistry markers included alanine aminotransferase (ALT) and aspartate aminotransferase (AST). Missingness was handled according to variable type. Missing continuous covariates were rare (<0.5%) and handled by mean imputation. For categorical covariates, including questionnaire-derived behavioural variables, missing values were retained as a separate “missing” category to preserve the analytic sample.

### Statistical analysis

Baseline characteristics were summarised as mean (standard deviation [SD]) or median (interquartile range [IQR]) for continuous variables, depending on their distribution, and as number (percentage) for categorical variables. Count-based cardiometabolic burden was analysed as an ordinal variable ranging from 1 to 5 and as a binary variable defined as high burden (≥3 CMRFs) versus low burden (<3 CMRFs) (10). The weighted cardiometabolic burden score was standardised and analysed per 1-SD increment and was additionally dichotomised at the median. The primary analyses were based on the composite CVD outcome, with secondary analyses conducted separately for CAD, stroke, AF, and HF. Traditional Cox proportional hazards models were first used to estimate the overall associations of cardiometabolic burden with incident CVD and all-cause mortality. To further characterise dynamic disease trajectories, multistate models were fitted using a clock-forward approach implemented with the R package mstate. Unlike conventional Cox models, multistate models estimate transition-specific associations while preserving the temporal ordering of disease states. Three prespecified transitions were modelled: baseline to CVD, baseline to death without prior CVD, and CVD to death. Subgroup analyses were conducted by age (<60 vs. ≥60 years) and sex, with interaction terms used to assess effect modification. For participants with CVD and death recorded on the same date, the CVD time was shifted 0.5 days earlier to establish event ordering required for the multistate model. In additional analyses, count-based cardiometabolic burden was modelled across all five categories, and the weighted score was analysed in quintiles. All models were adjusted for age, sex, self-reported ethnicity, smoking status, alcohol intake, physical activity, TDI, ALT, and AST. Effect estimates are presented as hazard ratios (HRs) with 95% confidence intervals (CIs). No evidence of non-proportionality was observed for the primary exposures, although global tests suggested departures in a minority of multistate models (Supplementary Table S8).

Three additional analyses were performed. First, to evaluate time-stratified associations, piecewise Cox models were fitted across prespecified follow-up intervals of 0–1, 1–3, and >3 years until the end of follow-up. Second, to describe disease-state occupancy over time, state occupation probabilities for remaining CVD-free, developing CVD, or dying were estimated at 1, 3, 5, 10, and 15 years. Third, to examine potential non-linearity, penalised spline models with 3 degrees of freedom were fitted, and likelihood ratio tests were used to compare non-linear and linear specifications.

Mediation analyses were conducted for inflammatory markers (Supplementary Table S9), including C-reactive protein, systemic immune-inflammation index (19), inflammatory burden index (20), and neutrophil-to-lymphocyte ratio (21), as well as lifestyle-related factors, including smoking and drinking status, physical activity, and dietary score (Supplementary Table S10). Because cardiometabolic burden and candidate mediators were assessed at baseline, mediation analyses were considered exploratory and interpreted as decompositions of observed associations rather than causal pathways. Causal mediation assumptions, including the absence of unmeasured confounding, cannot be fully verified in this observational setting. Mediation effects were estimated for each transition using g-computation with transition-specific Cox models, with 95% CIs obtained from 2,000 non-parametric bootstrap replications.

Sensitivity analyses included (i) excluding self-reported outcome events; (ii) excluding participants who developed CVD or died within the first year of follow-up; (iii) applying alternative time thresholds of 30 days, 180 days, 1 year, 2 years, and 5 years to determine event ordering when CVD and death occured on the same day; (iv) including participants with cancer at baseline; (v) using multiple imputation by chained equations for missing data; (vi) further adjusting for dietary score, C-reactive protein, and estimated glomerular filtration rate; and (vii) redefining CVD as a composite of CAD, stroke, and HF; (viii) conducting split-sample internal validation of the weighted cardiometabolic burden score by deriving the score in a randomly selected training set (60%) and applying the training-derived regression coefficients and standardisation parameters unchanged to the validation set (40%); (ix) Fine–Gray subdistribution hazard models were applied to evaluate whether accounting for the competing risk of death altered the associations observed in Cox models; (x) redefining MASLD within the UK Biobank imaging subcohort as MRI-derived proton density fat fraction ≥5% in combination with at least one cardiometabolic risk criterion (22) (Supplementary Table S11); (xi) assessing the robustness of non-linear associations by refitting penalised spline models using alternative degrees of freedom (2, 4–6); (xii) repeating the traditional Cox and multistate analyses after natural-log transformation of markedly right-skewed continuous covariates, including alcohol intake, ALT, and AST, to evaluate the robustness of the estimated associations to alternative covariate parameterisation.

All statistical tests were two-sided. Nominal *p* values < 0.05 were considered statistically significant unless otherwise specified. All analyses were performed using R software (version 4.2.3). For multistate and piecewise Cox models, *p* values were adjusted using the Benjamini–Hochberg false discovery rate procedure to account for multiple transition-specific and time-stratified comparisons; FDR-adjusted *p* values were used for interpretation, while both nominal and adjusted *p* values are presented. Subgroup analyses were assessed using interaction tests, and mediation analyses were considered exploratory secondary analyses.

## Results

### Baseline characteristics of the study population

During a median follow-up of 15.9 years, 15,352 of 72,922 participants with MASLD developed incident CVD, 4,273 died without prior CVD, and 4,228 died after incident CVD (Figure 1). Participants experiencing adverse transitions were generally older and had varying sex distributions across transition pathways, with a higher proportion of males in all transition groups. They were also more likely to smoke and experience greater socioeconomic deprivation, higher inflammatory marker levels, higher FLI, greater weighted cardiometabolic burden, and lower estimated glomerular filtration rate, with the most adverse profile observed among those who died after CVD. Count-based cardiometabolic burden shifted toward higher categories across adverse transition groups, particularly among participants who died after CVD. Detailed baseline characteristics are presented in Table 1.

**Figure 1:**
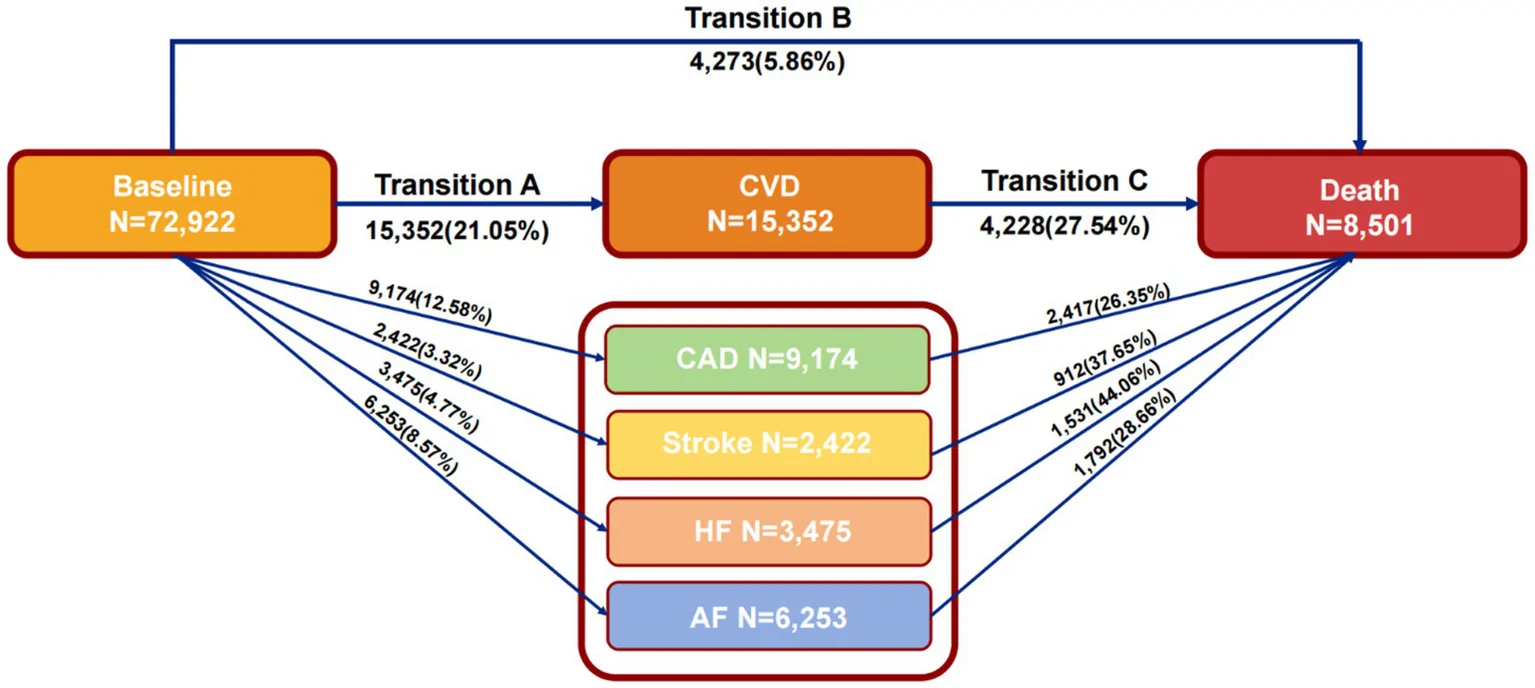
Multistate transitions from baseline to CVD and death in the study population (*N* = 72,922). The diagram illustrates the observed transitions among three health states: baseline, CVD, and death. The prespecified transition pathways included baseline to CVD (Transition A), baseline to death without prior CVD (Transition B), and CVD to death (Transition C). Numbers and percentages indicate the observed events for each transition. The inset shows the constituent CVD outcomes contributing to the composite CVD endpoint, including CAD, stroke, HF, and AF, with corresponding event counts and proportions. CVD: cardiovascular disease; CAD, coronary artery disease; HF, heart failure; AF, atrial fibrillation.

**Table 1:** Baseline characteristics of the study population across multi-state transition groups.

Variable	Total (*N* = 72,922)	Baseline to CVD (*N* = 15,352)	Baseline to death (*N* = 4,273)	CVD to death (*N* = 4,228)
Age (years)	56.84 (7.89)	60.06 (6.91)	60.95 (6.61)	62.33 (5.94)
Sex				
Female	29,931 (41%)	5,438 (35.4%)	1,695 (39.7%)	1,442 (34.1%)
Male	42,991 (59%)	9,914 (64.6%)	2,578 (60.3%)	2,786 (65.9%)
Race				
White	67,268 (92.2%)	14,371 (93.6%)	4,075 (95.4%)	4,009 (94.8%)
Black	1,586 (2.2%)	217 (1.4%)	68 (1.6%)	57 (1.3%)
Asian	2,399 (3.3%)	483 (3.1%)	68 (1.6%)	90 (2.1%)
Other	1,669 (2.3%)	281 (1.8%)	62 (1.5%)	72 (1.7%)
Smoking status				
Never	40,505 (55.5%)	7,386 (48.1%)	1,888 (44.2%)	1,700 (40.2%)
Previous	25,071 (34.4%)	6,095 (39.7%)	1,754 (41%)	1,810 (42.8%)
Current	7,011 (9.6%)	1,782 (11.6%)	601 (14.1%)	687 (16.2%)
Missing	335 (0.5%)	89 (0.6%)	30 (0.7%)	31 (0.7%)
Drinking status				
Never	5,449 (7.5%)	1,235 (8%)	309 (7.2%)	343 (8.1%)
Previous	4,550 (6.2%)	1,207 (7.9%)	365 (8.5%)	422 (10%)
Current	62,885 (86.2%)	12,902 (84%)	3,597 (84.2%)	3,460 (81.8%)
Missing	38 (0.1%)	8 (0.1%)	2 (0%)	3 (0.1%)
Physical activity				
Low	13,239 (18.2%)	2,792 (18.2%)	762 (17.8%)	830 (19.6%)
Moderate	22,091 (30.3%)	4,302 (28%)	1,190 (27.8%)	1,143 (27%)
High	18,760 (25.7%)	3,921 (25.5%)	1,078 (25.2%)	954 (22.6%)
Missing	18,832 (25.8%)	4,337 (28.3%)	1,243 (29.1%)	1,301 (30.8%)
Dietary score				
Low	54,897 (75.3%)	11,487 (74.8%)	3,191 (74.7%)	3,069 (72.6%)
High	14,363 (19.7%)	3,010 (19.6%)	826 (19.3%)	867 (20.5%)
Missing	3,662 (5%)	855 (5.6%)	256 (6%)	292 (6.9%)
ALT U/L	25.68 (19.58–34.64)	24.87 (19.21–33.47)	24.62 (19–32.99)	23.83 (18.33–31.54)
AST U/L	25.9 (22.2–30.8)	25.8 (22.1–30.8)	25.8 (22–31)	25.5 (21.7–30.8)
TDI	−1.06 (3.2)	−0.88 (3.28)	−0.8 (3.33)	−0.5 (3.41)
Alcohol intake (g/week)	60.8 (0–120)	56.8 (0–120)	50.4 (0–117.6)	41.6 (0–117.6)
CRP (mg/L)	1.91 (0.95–3.56)	2.02 (1–3.75)	2.21 (1.05–3.92)	2.24 (1.05–4.19)
NLR	2.12 (1.67–2.74)	2.17 (1.69–2.82)	2.19 (1.7–2.87)	2.25 (1.73–2.95)
SII	530.28 (392.42–711.93)	542.94 (398.47–731.94)	551.63 (403–754.47)	575.38 (418.54–798)
IBI	3.98 (1.89–8.32)	4.37 (2.08–9.13)	4.68 (2.2–9.95)	5.06 (2.29–10.87)
FLI	80.93 (70.67–90.66)	83.27 (72.48–92.67)	82.18 (71.61–92.01)	85.46 (73.63–94.14)
eGFR (mL/min/1.73 m^2^)	96.17 (85.26–103.14)	94.38 (82.47–100.69)	93.98 (82.04–100.02)	93.41 (80.7–99.24)
Count-based cardiometabolic burden				
Any 1 cardiometabolic factors	2,272 (3.1%)	260 (1.7%)	91 (2.1%)	50 (1.2%)
Any 2 cardiometabolic factors	13,300 (18.2%)	2,122 (13.8%)	625 (14.6%)	499 (11.8%)
Any 3 cardiometabolic factors	23,774 (32.6%)	4,398 (28.6%)	1,248 (29.2%)	999 (23.6%)
Any 4 cardiometabolic factors	21,528 (29.5%)	4,912 (32%)	1,328 (31.1%)	1,353 (32%)
Any 5 cardiometabolic factors	12,048 (16.5%)	3,660 (23.8%)	981 (23%)	1,327 (31.4%)
Weighted cardiometabolic score	0.64 (0.2)	0.69 (0.19)	0.68 (0.2)	0.72 (0.18)

Data are presented as mean (SD) or median (IQR) for continuous variables according to their distribution, and as number (%) for categorical variables. CVD, cardiovascular disease; ALT: alanine aminotransferase; AST: aspartate aminotransferase; TDI, Townsend deprivation index; CRP, C-reactive protein; NLR, neutrophil-to-lymphocyte ratio; SII, systemic immune-inflammation index; IBI, inflammatory burden index; eGFR, estimated glomerular filtration rate, calculated using the 2021 race-free Chronic Kidney Disease Epidemiology Collaboration (CKD-EPI) creatinine equation.; FLI, fatty liver index.

### Overall and transition-specific associations of cardiometabolic burden

In traditional Cox models, both count-based and weighted cardiometabolic burden were associated with higher risks of incident CVD and all-cause mortality (Table 2). For the count-based measure, each additional CMRF was associated with a 17% higher risk of CVD (HR 1.17, 95% CI 1.15–1.19) and a 17% higher risk of mortality (HR 1.17, 95% CI 1.14–1.19). Compared with low burden, high burden was associated with a 30% higher risk of CVD (HR 1.30, 95% CI 1.24–1.36) and a 22% higher risk of mortality (HR 1.22, 95% CI 1.15–1.30). For the weighted score, each 1-SD increment was associated with a 23% higher risk of CVD (HR 1.23, 95% CI 1.20–1.25) and a 22% higher risk of mortality (HR 1.22, 95% CI 1.19–1.25); high versus low weighted burden was associated with 34 and 30% higher risks, respectively (CVD: HR 1.34, 95% CI 1.29–1.38; mortality: HR 1.30, 95% CI 1.25–1.36).

**Table 2:** Associations of cardiometabolic burden with CVD and death evaluated by traditional cox and multi-state models.

Outcome/Transition	*N*	Events	Proportion (%)	HR (95%CI)	*p*-value	FDR
Traditional cox model						
Count-based Cardiometabolic Burden (Per 1 higher count)						
CVD	72,922	15,352	21.05%	1.17 (1.15–1.19)	<0.001	
Death	72,922	8,501	11.66%	1.17 (1.14–1.19)	<0.001	
Count-based Cardiometabolic Burden (High vs. low)						
CVD	57,350	12,970	22.62%	1.30 (1.24–1.36)	<0.001	
Death	57,350	7,236	12.62%	1.22 (1.15–1.30)	<0.001	
Weighted Cardiometabolic Burden Score (Per 1 higher SD)						
CVD	72,922	15,352	21.05%	1.23 (1.20–1.25)	<0.001	
Death	72,922	8,501	11.66%	1.22 (1.19–1.25)	<0.001	
Weighted Cardiometabolic Burden Score (high vs. low)						
CVD	31,942	8,130	25.45%	1.34 (1.29–1.38)	<0.001	
Death	31,942	4,695	14.70%	1.30 (1.25–1.36)	<0.001	
Multi-state model						
Count-based Cardiometabolic Burden (Per 1 higher count)						
Baseline to CVD	72,922	15,352	21.05%	1.17 (1.15–1.19)	<0.001	<0.001
Baseline to death	72,922	4,273	5.86%	1.09 (1.06–1.13)	<0.001	<0.001
CVD to death	15,352	4,228	27.54%	1.13 (1.10–1.17)	<0.001	<0.001
Count-based Cardiometabolic Burden (High vs. low)						
Baseline to CVD	57,350	12,970	22.62%	1.30 (1.24–1.36)	<0.001	<0.001
Baseline to death	57,350	3,557	6.20%	1.13 (1.05–1.23)	0.002	0.003
CVD to death	12,970	3,679	28.37%	1.14 (1.04–1.25)	0.005	0.005
Weighted Cardiometabolic Burden Score (Per 1 higher SD)						
Baseline to CVD	72,922	15,352	21.05%	1.23 (1.20–1.25)	<0.001	<0.001
Baseline to death	72,922	4,273	5.86%	1.12 (1.08–1.16)	<0.001	<0.001
CVD to death	15,352	4,228	27.54%	1.19 (1.15–1.23)	<0.001	<0.001
Weighted Cardiometabolic Burden Score (High vs. low)						
Baseline to CVD	31,942	8,130	25.45%	1.34 (1.29–1.38)	<0.001	<0.001
Baseline to death	31,942	2,171	6.80%	1.17 (1.10–1.24)	<0.001	<0.001
CVD to death	8,130	2,524	31.05%	1.22 (1.14–1.30)	<0.001	<0.001

The values are hazard ratios (HRs) and their 95% confidence intervals (CIs) from multivariable Cox proportional hazards models and Multistate models. Models were adjusted for age, sex, race, smoking status, alcohol intake, physical activity, TDI, ALT, and AST. Nominal *p* values are reported alongside false discovery rate (FDR)-adjusted *p* values, which were calculated using the Benjamini–Hochberg procedure separately for each cardiometabolic burden definition across the three prespecified multistate transitions. For continuous exposure analyses, N represents the total analytic sample; for high-versus-low analyses, *N* and Events represent the number of participants and events in the high-burden group, respectively, with the low-burden group included as the reference category. Proportion (%) was calculated as Events/*N* within the corresponding group. CVD, cardiovascular disease; ALT, alanine aminotransferase; AST, aspartate aminotransferase; TDI, Townsend deprivation index.

Multistate analyses showed that the magnitude of association varied across transition pathways (Table 2). For count-based burden, each additional CMRF was associated with higher risks of the baseline-to-CVD (HR 1.17, 95% CI 1.15–1.19), baseline-to-death (HR 1.09, 95% CI 1.06–1.13), and CVD-to-death transitions (HR 1.13, 95% CI 1.10–1.17). In categorical analyses, high vs. low burden was associated with higher risks across these transitions: HR 1.30 (95% CI 1.24–1.36), HR 1.13 (95% CI 1.05–1.23), and HR 1.14 (95% CI 1.04–1.25), respectively. For the weighted score, each 1-SD increment was associated with higher risks of the baseline-to-CVD (HR 1.23, 95% CI 1.20–1.25), baseline-to-death (HR 1.12, 95% CI 1.08–1.16), and CVD-to-death transitions (HR 1.19, 95% CI 1.15–1.23). High vs. low weighted burden showed corresponding HRs of 1.34 (95% CI 1.29–1.38), 1.17 (95% CI 1.10–1.24), and 1.22 (95% CI 1.14–1.30), respectively.

When CAD, stroke, AF, and HF were analysed separately, the associations were generally directionally consistent with the primary composite CVD analysis, although the magnitude varied across individual outcomes (Supplementary Tables S12–S15).

### Graded associations across burden categories

In traditional Cox models, both the count-based cardiometabolic burden and weighted burden score showed graded associations with CVD. In contrast, associations with mortality were mainly observed at higher burden levels. For the count-based measure, mortality risk increased among participants with four CMRFs (HR: 1.33; 95% CI: 1.13–1.58) and five CMRFs (HR: 1.78; 95% CI: 1.50–2.11). For the weighted score, higher mortality risks were evident from the third quintile onward, with HRs of 1.25 (95% CI: 1.14–1.36), 1.26 (95% CI: 1.16–1.36), and 1.67(95% CI: 1.54–1.81) for the third, fourth, and fifth quintiles, respectively (Supplementary Table S16).

Multi-state models showed similar patterns. Both burden measures were associated with the transition from baseline to CVD across increasing burden levels, whereas associations with baseline-to-death transition were largely restricted to the highest burden categories: five CMRFs (HR: 1.38; 95% CI: 1.12–1.72) and the highest weighted-score quintile (HR: 1.30; 95% CI: 1.17–1.45). For the CVD-to-death transition, the count-based measure was significantly associated with mortality only in participants with five CMRFs (HR: 1.58; 95% CI: 1.19–2.09) (Supplementary Table S16; Figure 2), whereas the weighted score showed consistent associations from the third quintile onward, with HRs of 1.26(95% CI: 1.10–1.44), 1.17(95% CI: 1.03–1.34), and 1.51(95% CI: 1.33–1.72) for the third, fourth, and fifth quintiles, respectively (Supplementary Table S16; Figure 2).

**Figure 2:**
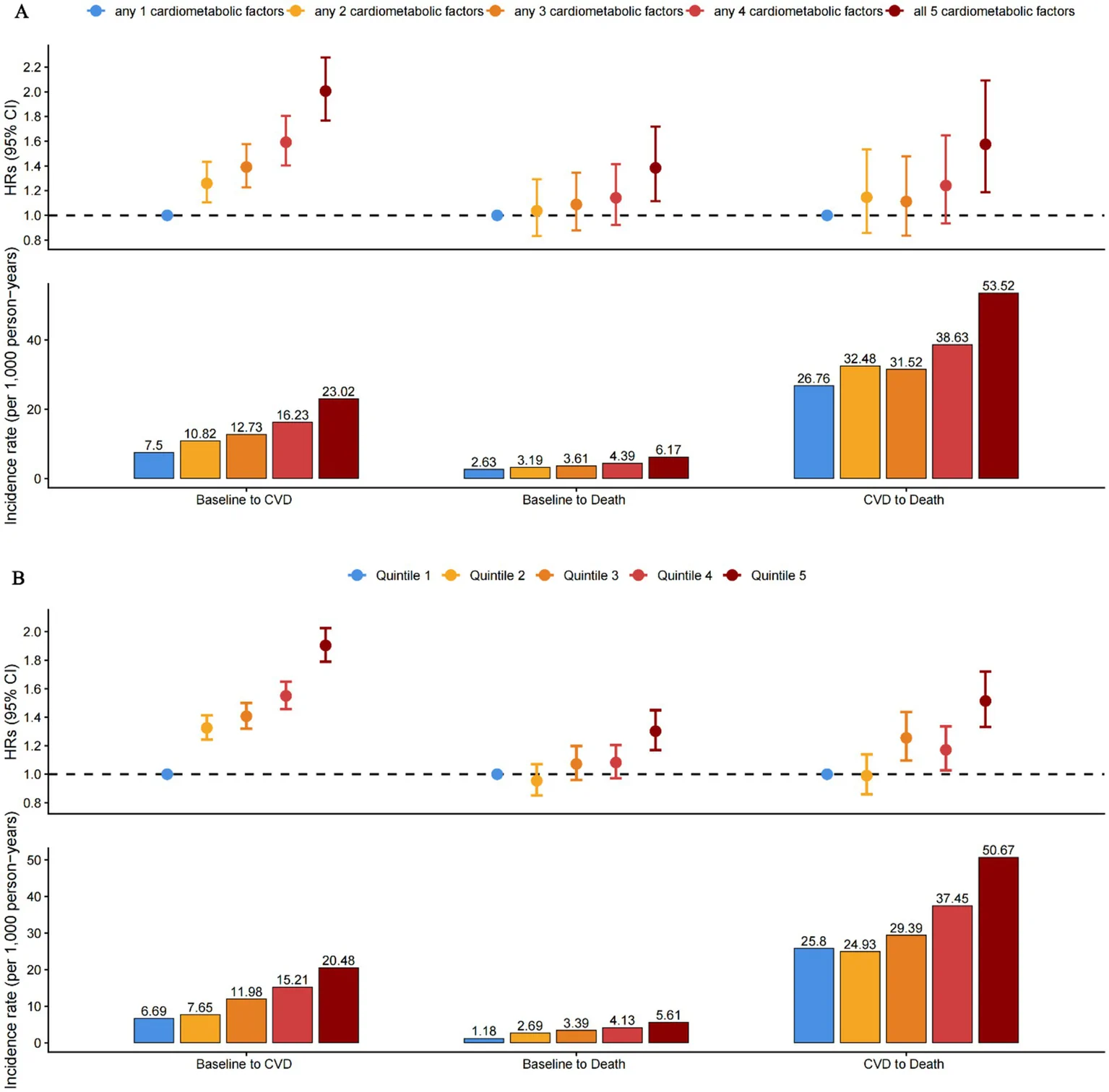
Graded associations of cardiometabolic burden categories with CVD and death in traditional cox and multi-state models. **(A)** Shows hazard ratios (HRs) and 95% confidence intervals (CIs), together with the corresponding incidence rates per 1,000 person-years, for transitions from baseline to CVD, baseline to death, and CVD to death according to categories of Count-based Cardiometabolic Burden. **(B)** Shows HRs, 95% CIs, and corresponding incidence rates per 1,000 person-years for the same transition pathways according to quintiles of the Weighted Cardiometabolic Burden Score. The reference groups were participants with any 1 cardiometabolic factor in **(A)** and those in Quintile 1 in **(B)**. Models were adjusted for age, sex, race, smoking status, alcohol intake, physical activity, TDI, ALT, and AST. CVD, cardiovascular disease.

In subtype-specific multi-state analyses of CAD, stroke, AF, and HF, association patterns varied across CVD outcomes. The weighted burden score showed more consistent associations with baseline-to-death transitions (Supplementary Tables S17–S20).

### Temporal patterns of association

In time-stratified analyses, both burden measures were associated with the baseline-to-CVD transition across all follow-up intervals, with the strongest associations observed early after baseline and attenuation over time (Figure 3). In contrast, transitions involving death showed more pronounced temporal heterogeneity.

**Figure 3:**
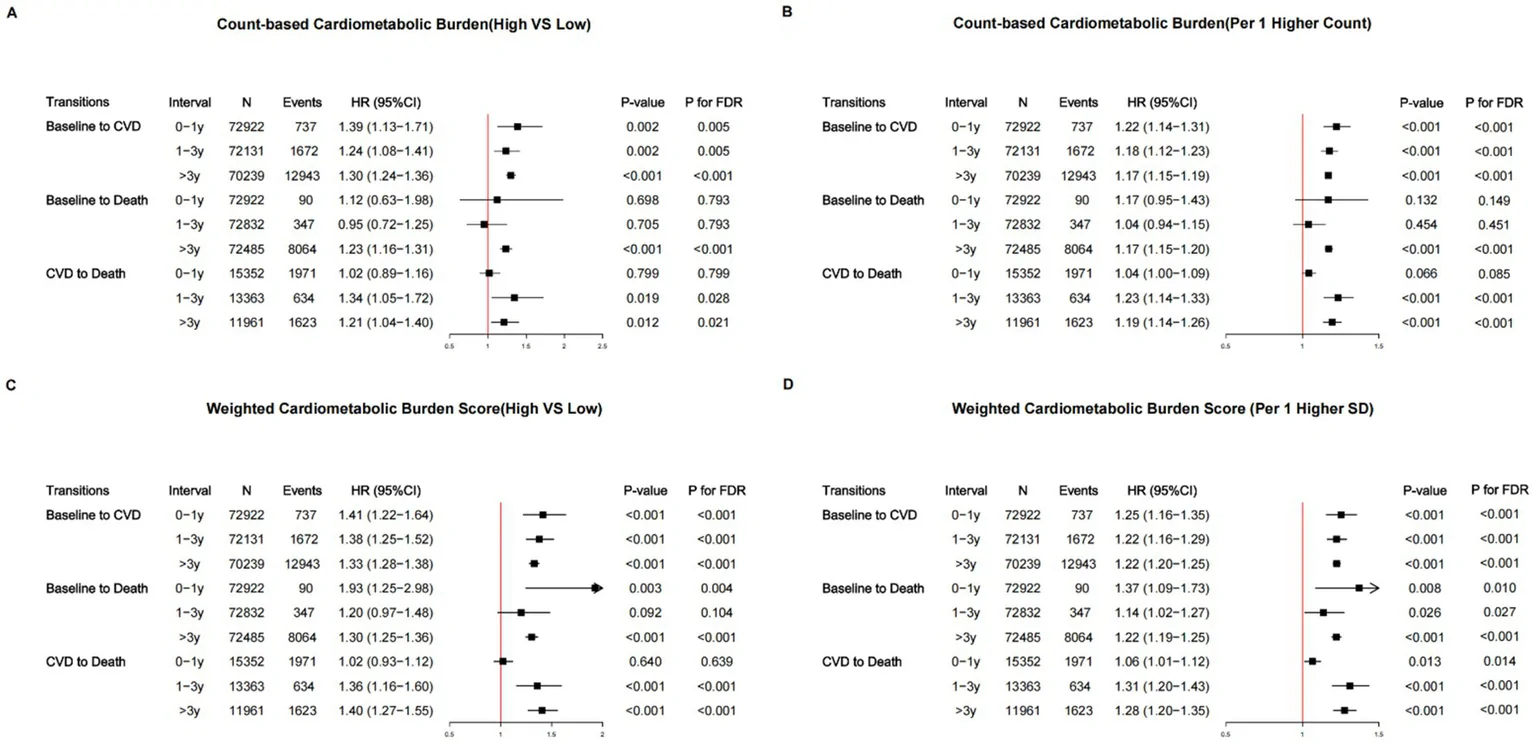
Time-stratified associations between cardiometabolic burden and risks of transitions from baseline to CVD, baseline to death, and CVD to death. Piecewise Cox models were adjusted for age, sex, race, smoking status, alcohol intake, physical activity, TDI, ALT, and AST. Time-specific hazard ratios (*HRs*) and 95% confidence intervals (*CIs*) for the associations of cardiometabolic burden with transitions from baseline to CVD, baseline to death, and CVD to death were estimated within predefined follow-up intervals (0–1 year, 1–3 years, and >3 years). Nominal *p* values are reported alongside *FDR*-adjusted *p* values, which were calculated using the Benjamini–Hochberg. CVD, cardiovascular disease.

For the baseline-to-death transition, count-based burden was associated with risk mainly during longer-term follow-up. Beyond 3 years, both high vs. low burden and each additional CMRF were associated with higher risk (HR 1.23, 95% CI 1.16–1.31; HR 1.17, 95% CI 1.15–1.20, respectively). The weighted score showed associations during both early and late follow-up. During the first year, high vs. low weighted burden was associated with higher risk (HR 1.93, 95% CI 1.25–2.98), as was each 1-SD increment (HR 1.37, 95% CI 1.09–1.73). Beyond 3 years, the corresponding HRs were 1.30 (95% CI 1.25–1.36) and 1.22 (95% CI 1.19–1.25), respectively.

For the CVD-to-death transition, count-based burden was not associated with risk during the first year but became associated during intermediate and longer-term follow-up. During 1–3 years, high vs. low burden was associated with an HR of 1.34 (95% CI 1.05–1.72), and each additional CMRF with an HR of 1.23 (95% CI 1.14–1.33). Beyond 3 years, the corresponding HRs were 1.21 (95% CI 1.04–1.40) and 1.19 (95% CI 1.14–1.26), respectively. For the weighted score, high vs. low burden was associated with higher risk during 1–3 years (HR 1.36, 95% CI 1.16–1.60) and beyond 3 years (HR 1.40, 95% CI 1.27–1.55). When analysed continuously, the weighted score was associated with CVD-to-death risk across all intervals, with HRs of 1.06 (95% CI 1.01–1.12), 1.31 (95% CI 1.20–1.43), and 1.28 (95% CI 1.20–1.35) for 0–1, 1–3, and >3 years, respectively.

### Dose–response patterns and transition probabilities

Penalised spline analyses revealed non-linear associations between the standardised weighted cardiometabolic burden score and all three transitions, including baseline to CVD, baseline to death, and CVD to death (Figure 4). Across transition pathways, risk increased progressively with higher burden levels, with steeper gradients at the upper end of the distribution.

**Figure 4:**
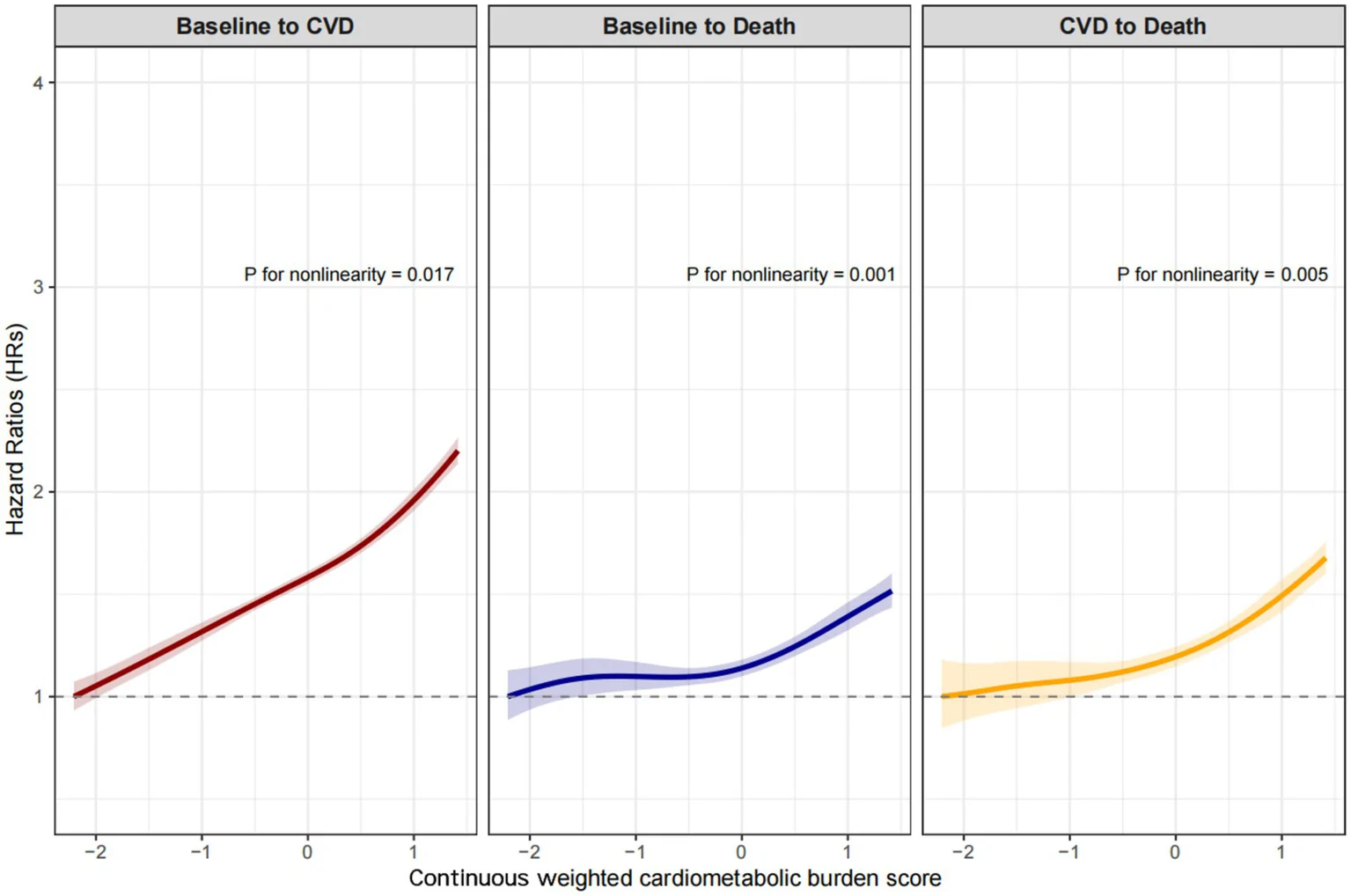
Non-linear associations of the Weighted Cardiometabolic Burden Score with transition risks in participants with MASLD. Penalised spline curves show the associations between the Weighted Cardiometabolic Burden Score and the risks of transition from baseline to CVD, baseline to death, and CVD to death. Solid lines indicate hazard ratios (HRs), and shaded areas indicate 95% confidence intervals (CIs). *p* values for non-linearity were derived from likelihood ratio tests comparing penalised spline models with the corresponding linear models. Models were adjusted for age, sex, race, smoking status, alcohol intake, physical activity, TDI, ALT, and AST. *p* values for non-linearity are presented in each panel. CVD, cardiovascular disease.

Transition probabilities derived from the multistate model further illustrated these associations in absolute terms. At 15 years, participants in the high-burden category had higher estimated probabilities of CVD and death, and a lower probability of remaining CVD-free, than those in the low-burden category (Supplementary Table S21). These differences widened over follow-up and were observed at 1, 3, 5, 10, and 15 years.

### Mediation analysis

Exploratory mediation analyses decomposed the observed associations between cardiometabolic burden and disease-state transitions into direct and indirect components (Supplementary Tables S22–S29). The estimated indirect components corresponding to inflammatory markers were generally small, with the largest proportions being approximately 3% across transitions. Lifestyle-related factors showed relatively larger estimated indirect proportions in the observed associations, particularly smoking and drinking status (Figure 5). For count-based burden, the estimated proportions corresponding to smoking were 4.40, 14.66, and 11.15% for the baseline-to-CVD, baseline-to-death, and CVD-to-death transitions, respectively; the corresponding estimates for drinking status were 3.22, 5.11, and 3.40%. For the weighted score, the corresponding estimates for smoking were 1.68, 6.19, and 4.13%, and those for drinking status were 2.12, 2.99, and 2.40%, respectively.

**Figure 5:**
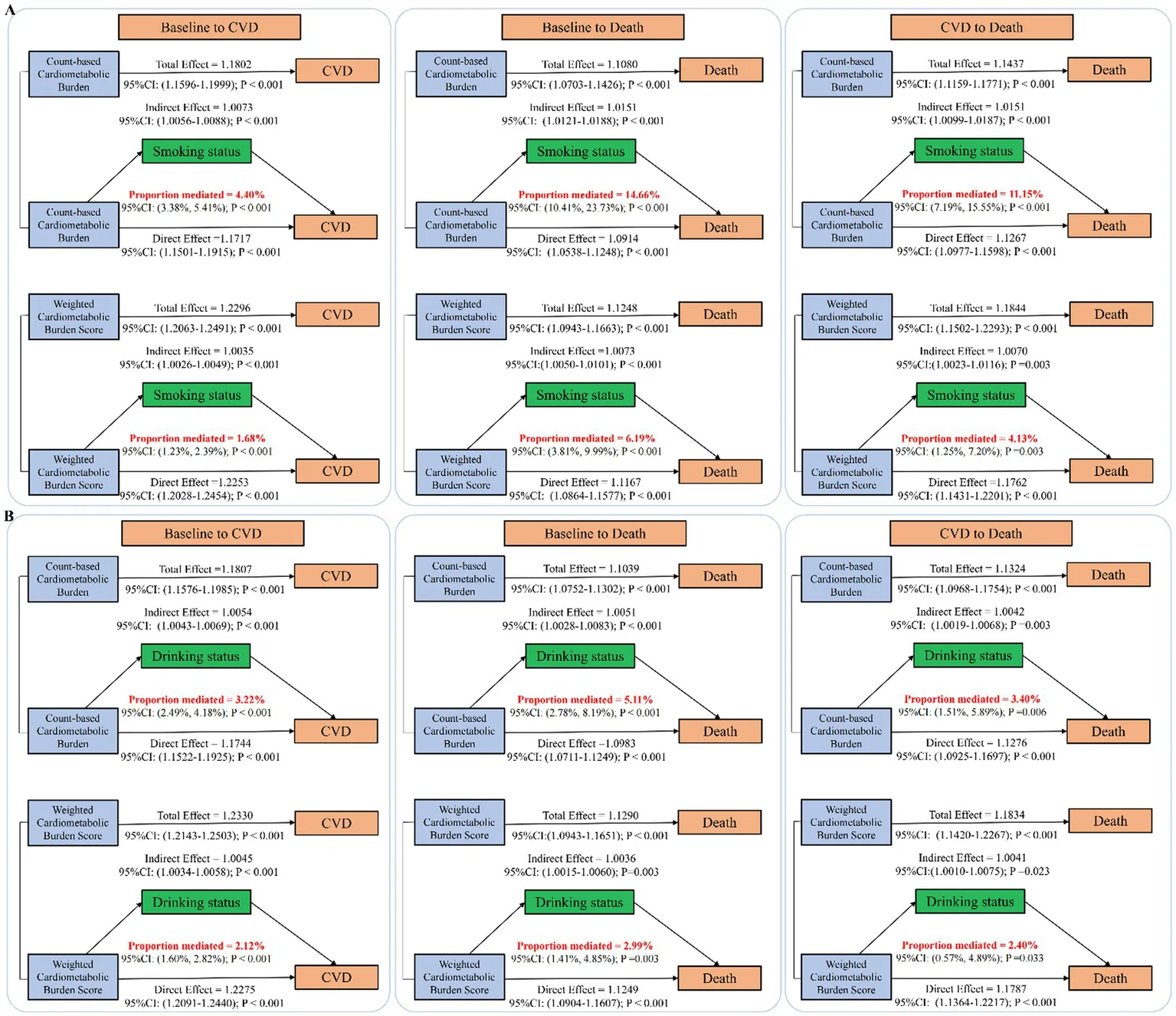
Estimated indirect components associated with drinking status and smoking status in the associations between cardiometabolic burden and multistate disease transitions. Panel **(A)** presents mediation by smoking status, and Panel **(B)** presents mediation by drinking status. Mediation was evaluated for transitions from baseline to CVD, baseline to death, and CVD to death, using both Count-based Cardiometabolic Burden and the weighted cardiometabolic burden score as exposures. Total, direct, and indirect effects, together with the proportion mediated, are shown with 95% confidence intervals and *p* values. Analyses were adjusted for age, sex, race, smoking status, alcohol intake, physical activity, TDI, ALT, and AST; however, when a given factor was evaluated as a mediator, it was not simultaneously included as a covariate in the corresponding model. Values shown in red or bold indicate mediation proportions that are statistically significant (*p* < 0.05). CVD: cardiovascular disease.

### Subgroup and sensitivity analyses

In subgroup analyses, associations with the baseline-to-CVD transition were consistently stronger among participants aged <60 years than among those aged ≥60 years. Similar age-related differences were observed for the baseline-to-death transition in most models. By contrast, associations were generally comparable between men and women across transition pathways (Supplementary Table S30; Supplementary Figure S3).

Across sensitivity analyses, associations of both count-based cardiometabolic burden and the weighted score with CVD, all-cause mortality, and the three transition-specific outcomes remained broadly consistent with the primary findings. In addition, sensitivity analyses using alternative penalised spline specifications (2–6 degrees of freedom) yielded broadly similar non-linear patterns across alternative spline specifications. In the MRI-PDFF-defined MASLD subgroup, estimates for incident CVD and the baseline-to-CVD transition were broadly comparable in direction and magnitude to those observed in the primary analysis. Estimates for the baseline-to-death and CVD-to-death transitions generally pointed in the same direction but were imprecise, with wider confidence intervals, likely owing to the substantially smaller imaging subgroup and the limited number of mortality events (Supplementary Tables S31–S42; Supplementary Figures S4–S7).

## Discussion

In this large prospective study of over 72,000 UK Biobank participants with MASLD, greater cardiometabolic burden was associated with higher risks of incident CVD and all-cause mortality. When examined within a multistate framework, these associations were not uniform across disease-state transitions. The strongest association was observed for the baseline-to-CVD transition, whereas associations with death-related transitions were smaller in magnitude but became more evident over longer follow-up. The weighted cardiometabolic burden score provided complementary estimates of the associations with incident CVD, all-cause mortality, and disease-state transitions alongside the count-based measure. Associations between the weighted score and transition risks were non-linear, with steeper gradients observed at higher burden levels. Mediation analyses suggested that inflammatory markers and lifestyle-related factors were associated with the estimated indirect components of these associations, with modest proportions for inflammatory markers and relatively larger proportions for smoking and drinking status.

These findings extend prior evidence linking cardiometabolic burden to adverse outcomes in MASLD. Prior studies have linked greater CMRF burden and its longitudinal worsening to incident CVD and mortality in MASLD. Our estimate for incident CVD per additional CMRF (HR 1.17) closely matched that of a nationwide cohort (HR 1.18) (8), whereas the association with all-cause mortality for five versus one CMRF was weaker than that reported in NHANES (HR 1.78 vs. 3.57), potentially reflecting differences in study populations, outcome ascertainment, and covariate adjustment (9). The present study builds on this work by placing cardiometabolic burden within a multistate framework that distinguishes three clinically relevant transitions across the disease life course: CVD onset from a disease-free state, death without prior CVD, and death following CVD. This approach provides a more granular description of how cardiometabolic burden relates to life-course disease progression than conventional endpoint-based survival analyses, which do not distinguish these sequential pathways. Importantly, this framework showed that cardiometabolic burden was more strongly associated with CVD onset than with death-related transitions, a distinction that could not be resolved by traditional Cox models of overall CVD and mortality.

The observation that cardiometabolic burden was most strongly associated with the baseline-to-CVD transition is biologically plausible. Cardiometabolic abnormalities are closely linked to chronic systemic inflammation (23–25), insulin resistance, endothelial dysfunction (26, 27), and atherogenic dyslipidaemia (28, 29), all of which are implicated in endothelial injury, atherogenic lipoprotein metabolism, and atherosclerotic progression. These processes may be particularly relevant before clinical CVD has developed, when cardiometabolic burden remains a major marker of underlying vascular and metabolic susceptibility. Once CVD has occurred, however, the risk set changes substantially. Individuals entering the CVD state already have a markedly higher baseline risk of death, and subsequent prognosis may be influenced by disease-specific factors, including myocardial remodelling, plaque instability, arrhythmogenic substrate, treatment response, and competing comorbidities. In the post-CVD state, the relative association of cardiometabolic burden with subsequent mortality may be attenuated against a higher underlying risk of death, despite continued clinical relevance. These more modest estimates should also be interpreted in light of the smaller number of post-CVD deaths, which limited statistical precision.

The time-stratified analyses further refine this interpretation. The association with incident CVD was strongest during early follow-up, whereas associations with death-related transitions became more apparent over longer follow-up. This pattern is consistent with a prolonged cumulative process in which persistent cardiometabolic dysfunction may be linked not only to cardiovascular complications but also to liver disease progression, multiorgan injury, and extrahepatic sequelae (30). Accordingly, weaker short-term associations with death-related transitions should not be interpreted as indicating limited relevance for long-term survival; rather, mortality risk in MASLD may emerge through a longer and more complex disease trajectory. Taken together, these findings suggest stage-specific associations between cardiometabolic burden and MASLD progression, with the strongest associations observed for CVD onset and death-related associations becoming more apparent over longer follow-up. However, later-interval estimates are conditional on survival and remaining in the relevant risk state, and individuals who died early may represent a clinically higher-risk subgroup. Survivor selection and selective depletion of susceptible individuals may therefore partly contribute to the observed temporal variation, and these findings should not be interpreted as evidence of a delayed causal effect.

The weighted cardiometabolic burden score provided a complementary representation of cardiometabolic burden, with some heterogeneity in risk estimates across components and time-stratified models. This observation is consistent with the possibility that individual cardiometabolic risk factors may differ in their associations with disease progression in MASLD. Component-level analyses suggested that hypertension was more strongly associated with incident CVD than several other metabolic abnormalities, whereas hypertriglyceridaemia showed weaker associations, in line with prior evidence that conventional metabolic abnormalities do not contribute uniformly to disease progression in MASLD (11). These differences suggest that similar CMRF counts may represent heterogeneous risk profiles, highlighting a potential limitation of simple counts in capturing component-specific risk. A recent UK Biobank proteomic study identified distinct MASLD cardiometabolic endotypes with divergent survival profiles and molecular signatures involving hepatic lipid metabolism, one-carbon metabolism, and immune–vascular integrity. These findings provide complementary molecular context for the heterogeneity observed across conventional cardiometabolic components, suggesting that similar aggregate burden may reflect biologically distinct disease processes (31). Accordingly, the weighted score may offer an exploratory means of summarising component-level heterogeneity alongside the count-based measure. Its association with the baseline-to-CVD transition may partly reflect outcome-informed weighting because the score was derived using incident CVD coefficients and should therefore be interpreted cautiously. Moreover, given its internal derivation and lack of external validation, the weighted score should be regarded solely as a supplementary analytical measure rather than as evidence of improved prediction or a validated clinical risk tool. Its internal derivation within the same cohort may also have introduced some degree of overfitting and optimistic estimation.

Mediation analyses suggested that lifestyle-related factors, particularly smoking and drinking status, showed larger estimated indirect proportions in the observed associations between cardiometabolic burden and disease-state transitions than inflammatory markers. Although inflammatory markers showed only small estimated indirect proportions in these associations, this does not exclude a role of systemic inflammation in MASLD progression. Rather, it suggests that circulating inflammatory markers may capture only one component of a broader cardiometabolic milieu involving metabolic dysregulation, vascular injury, hepatic dysfunction, inflammation, and behavioural factors. Prior studies have suggested that cardiometabolic dysfunction in MASLD is often accompanied by chronic low-grade inflammation, greater symptom burden, and psychological stress, which may be associated with unhealthy behaviours (32). In turn, smoking and alcohol exposure are associated with both hepatic and cardiovascular injury (33, 34). These findings suggest that smoking and drinking status may represent modifiable risk-related factors among individuals with MASLD and high cardiometabolic burden. However, because cardiometabolic burden and the potential mediators were assessed at baseline, temporal ordering could not be established and reverse causation cannot be excluded. These analyses should therefore be interpreted as an exploratory decomposition of observed associations rather than evidence of causal mediation. Moreover, cardiometabolic burden and lifestyle factors may share common socioeconomic and behavioural determinants, and their relationships may not follow a simple unidirectional pathway. Whether lifestyle modification attenuates cardiovascular and mortality risk in this population requires confirmation in prospective and intervention studies.

Age-stratified analyses showed stronger associations of cardiometabolic burden with the baseline-to-CVD and baseline-to-death transitions among participants younger than 60 years. Younger individuals generally have fewer competing risks and comorbidities, whereas the greater competing-risk burden in older adults may attenuate relative associations and partly contribute to the observed age heterogeneity. Earlier exposure to an adverse metabolic milieu may also translate into a longer cumulative burden across the life course, consistent with previous reports that younger individuals are particularly susceptible to the cumulative effects of metabolic syndrome and related risk factors (9, 35, 36). These findings suggest that younger individuals with MASLD and high cardiometabolic burden may represent a vulnerable subgroup. Earlier identification and timely intervention may therefore be important to reduce cumulative cardiometabolic exposure and mitigate later CVD and mortality risk in this group.

## Limitations

Several limitations should be acknowledged. First, the UK Biobank predominantly comprises White, middle-aged adults and is subject to healthy-volunteer bias. Compared with the general UK population, participants generally have more favourable socioeconomic and health profiles, and absolute transition probabilities may therefore be lower than those in the broader population. Although differences in baseline risk do not necessarily imply biased relative associations, selective participation may still affect exposure–outcome relationships, with the direction and magnitude of such bias remaining uncertain. Generalisability to more diverse and less healthy populations is therefore limited. Second, hepatic steatosis was defined using the FLI rather than direct imaging or histology. Because FLI incorporates cardiometabolic components, some degree of exposure-related misclassification or selection cannot be excluded. The magnitude and direction of any resulting bias are uncertain and may differ across transition pathways. Findings for incident CVD and the baseline-to-CVD transition were broadly consistent in the MRI-PDFF-defined MASLD subgroup, although the smaller imaging sample and limited number of deaths reduced precision for mortality-related transitions. Third, cardiometabolic risk factors and covariates were assessed only at baseline. Changes in metabolic burden and treatment during follow-up may differentially influence transition-specific associations; in particular, treatment intensification after incident CVD may attenuate the association between baseline burden and subsequent CVD-to-death risk. Fourth, outcome misclassification cannot be excluded because CVD events were ascertained from multiple linked clinical and self-reported data sources. Nevertheless, the integration of these complementary sources and concordant sensitivity analyses supports the robustness of the primary findings. Fifth, Mediation analyses were exploratory; because exposure and candidate mediators were measured at baseline, temporal ordering could not be established, and the assumptions required for causal mediation (including absence of unmeasured exposure–mediator and mediator–outcome confounding) cannot be fully verified in observational data. Therefore, these findings should be interpreted as decompositions of observed associations rather than causal pathways. Finally, residual confounding from unmeasured or incompletely measured factors, such as family history of CVD and treatment-related characteristics, cannot be excluded, and the observational design does not support causal inference.

## Conclusion

In participants with MASLD, greater cardiometabolic burden was associated with higher risks of CVD and death across all three disease-state transitions. The strongest association was observed for incident CVD, particularly during early follow-up, whereas associations with death-related transitions were more consistent with longer-term cumulative risk. Smoking and drinking status showed relatively larger estimated indirect proportions in these associations, highlighting their potential relevance as modifiable factors for further investigation. Although associations were observed across age groups, they were most pronounced in younger participants, suggesting that individuals under 60 years with MASLD and high cardiometabolic burden may represent a priority subgroup for early identification, targeted prevention, and sustained cardiometabolic surveillance.

## Data Availability

The data supporting the conclusions of this article are available via the UK BioBank. Access to the UK Biobank resource can be obtained via an approved application https://www.ukbiobank.ac.uk and https://biobank.ndph.ox.ac.uk/ukb.

## References

[1] Eslam M Sanyal AJ George J. MAFLD: a consensus-driven proposed nomenclature for metabolic associated fatty liver disease. Gastroenterology. (2020) 158:1999–2014.e1. doi: 10.1053/j.gastro.2019.11.312, 32044314

[2] Rinella ME Lazarus JV Ratziu V Francque SM Sanyal AJ Kanwal F . A multisociety Delphi consensus statement on new fatty liver disease nomenclature. J Hepatol. (2023) 79:1542–56. doi: 10.1016/j.jhep.2023.06.003 37364790

[3] Miao L Targher G Byrne CD Cao Y-Y Zheng M-H. Current status and future trends of the global burden of MASLD. Trends Endocrinol Metab. (2024) 35:697–707. doi: 10.1016/j.tem.2024.02.007, 38429161

[4] Fan X Zhang B Shi Y Liu L Zhao J. Systemic metabolic abnormalities: key drivers of complications and mortality in MASLD. J Hepatol. (2024) 80:e246–8. doi: 10.1016/j.jhep.2024.02.001, 38311122

[5] Kwak M Kim H-S Jiang ZG Yeo YH Trivedi HD Noureddin M . MASLD/MetALD and mortality in individuals with any cardio-metabolic risk factor: a population-based study with 26.7 years of follow-up. Hepatology. (2025) 81:228–37. doi: 10.1097/HEP.0000000000000925, 38739848 PMC12169893

[6] Mottillo S Filion KB Genest J Joseph L Pilote L Poirier P . The metabolic syndrome and cardiovascular risk a systematic review and meta-analysis. J Am Coll Cardiol. (2010) 56:1113–32. doi: 10.1016/j.jacc.2010.05.034 20863953

[7] Li J Liu W Li H Ye X Qin JJ. Changes of metabolic syndrome status alter the risks of cardiovascular diseases, stroke and all cause mortality. Sci Rep. (2025) 15:5448. doi: 10.1038/s41598-025-86385-1, 39952953 PMC11828854

[8] Lee H-H Lee HA Kim E-J Kim HY Kim HC Ahn SH . Cardiovascular risk from metabolic dysfunction-associated Steatotic liver disease, Cardiometabolic risk factor count, and their longitudinal changes: a Nationwide cohort study. Am J Gastroenterol. (2025) 120:2321–9. doi: 10.14309/ajg.0000000000003274, 39787330

[9] Wen Y Min Y Lei Y Wei Z. Cardiometabolic dysfunction burden and mortality outcomes in metabolic dysfunction-associated steatotic liver disease. PLoS One. (2025) 20:e0327772. doi: 10.1371/journal.pone.0327772, 40608810 PMC12225798

[10] Cuthbertson DJ Kennedy OJ Bilson J Hydes TJ Targher G Glyn-Owen K . Impact of metabolic dysfunction severity in steatotic liver disease and its interaction with liver fibrosis on all-cause mortality and multiple hepatic and extra-hepatic outcomes. Metabolism. (2025) 170:156306. doi: 10.1016/j.metabol.2025.156306, 40414560

[11] Dukewich M Dodge JL Yuan L Terrault NA. Differential effects of cardiometabolic risk factors on all-cause mortality in United States adults with metabolic dysfunction-associated steatotic liver disease (MASLD). Clin Gastroenterol Hepatol. (2025) 24:1344–53. doi: 10.1016/j.cgh.2025.09.003, 40972880 PMC12704451

[12] Littlejohns TJ Sudlow C Allen NE Collins R. UK biobank: opportunities for cardiovascular research. Eur Heart J. (2019) 40:1158–66. doi: 10.1093/eurheartj/ehx254, 28531320 PMC6451771

[13] Sudlow C Gallacher J Allen N Beral V Burton P Danesh J . UK biobank: an open access resource for identifying the causes of a wide range of complex diseases of middle and old age. PLoS Med. (2015) 12:e1001779. doi: 10.1371/journal.pmed.1001779, 25826379 PMC4380465

[14] Wu S Yuan C Yang Z Liu S Zhang Q Zhang S . Non-alcoholic fatty liver is associated with increased risk of irritable bowel syndrome: a prospective cohort study. BMC Med. (2022) 20:262. doi: 10.1186/s12916-022-02460-8, 35989356 PMC9394037

[15] Ho B Thompson A Jorgensen AL Pirmohamed M. Role of fatty liver index in risk-stratifying comorbid disease outcomes in non-alcoholic fatty liver disease. JHEP Rep. (2023) 5:100896. doi: 10.1016/j.jhepr.2023.100896, 37928746 PMC10624587

[16] Jones GS Alvarez CS Graubard BI McGlynn KA. Agreement between the prevalence of nonalcoholic fatty liver disease determined by transient Elastography and fatty liver indices. Clin Gastroenterol Hepatol. (2022) 20:227–229.e2. doi: 10.1016/j.cgh.2020.11.028, 33227430 PMC8529633

[17] Huo Z Chen Y Huang Y Yang Z Long Y Zhang Q . Long-term prognosis of lean MASLD: evidence from three population-based prospective cohorts. Gut. (2026) 75:772–85. doi: 10.1136/gutjnl-2025-336127, 41093635

[18] Ding C Ng Fat L Britton A Im PK Lin K Topiwala A . Binge-pattern alcohol consumption and genetic risk as determinants of alcohol-related liver disease. Nat Commun. (2023) 14:8041. doi: 10.1038/s41467-023-43064-x, 38097541 PMC10721893

[19] Hu B Yang X-R Xu Y Sun YF Sun C Guo W . Systemic immune-inflammation index predicts prognosis of patients after curative resection for hepatocellular carcinoma. Clin Cancer Res. (2014) 20:6212–22. doi: 10.1158/1078-0432.CCR-14-0442, 25271081

[20] Lu W Liao X Jiang Y Luo B Chen L Lin Y. Association between inflammatory burden index and prognosis in patients with coronary heart disease: a retrospective study. PLoS One. (2025) 20:e0325287. doi: 10.1371/journal.pone.0325287, 40622923 PMC12233253

[21] Wang X Zhang G Jiang X Zhu H Lu Z Xu L. Neutrophil to lymphocyte ratio in relation to risk of all-cause mortality and cardiovascular events among patients undergoing angiography or cardiac revascularization: a meta-analysis of observational studies. Atherosclerosis. (2014) 234:206–13. doi: 10.1016/j.atherosclerosis.2014.03.003, 24681815

[22] Fan W Yang S Wei Y Tian M Liu Q Li X . Characterization of brain morphology associated with metabolic dysfunction-associated steatotic liver disease in the UK biobank. Diabetes Obes Metab. (2025) 27:3419–30. doi: 10.1111/dom.16362, 40171859

[23] Senn JJ Klover PJ Nowak IA Mooney RA. Interleukin-6 induces cellular insulin resistance in hepatocytes. Diabetes. (2002) 51:3391–9. doi: 10.2337/diabetes.51.12.3391, 12453891

[24] Yuen DYC Dwyer RM Matthews VB Zhang L Drew BG Neill B . Interleukin-6 attenuates insulin-mediated increases in endothelial cell signaling but augments skeletal muscle insulin action via differential effects on tumor necrosis factor-alpha expression. Diabetes. (2009) 58:1086–95. doi: 10.2337/db08-0775 PMC2671037 19188427

[25] Tilg H Moschen AR. Evolution of inflammation in nonalcoholic fatty liver disease: the multiple parallel hits hypothesis. Hepatology. (2010) 52:1836–46. doi: 10.1002/hep.24001, 21038418

[26] Nasiri-Ansari N Androutsakos T Flessa C-M Kyrou I Siasos G Randeva HS . Endothelial cell dysfunction and nonalcoholic fatty liver disease (NAFLD): a concise review. Cells. (2022) 11:2511. doi: 10.3390/cells11162511, 36010588 PMC9407007

[27] Persico M Masarone M Damato A Ambrosio M Federico A Rosato V . Non alcoholic fatty liver disease and eNOS dysfunction in humans. BMC Gastroenterol. (2017) 17:35. doi: 10.1186/s12876-017-0592-y, 28264657 PMC5340006

[28] Targher G Byrne CD Tilg H. NAFLD and increased risk of cardiovascular disease: clinical associations, pathophysiological mechanisms and pharmacological implications. Gut. (2020) 69:1691–705. doi: 10.1136/gutjnl-2020-320622, 32321858

[29] Ståhlman M Pham HT Adiels M Mitchell TW Blanksby SJ Fagerberg B . Clinical dyslipidaemia is associated with changes in the lipid composition and inflammatory properties of apolipoprotein-B-containing lipoproteins from women with type 2 diabetes. Diabetologia. (2012) 55:1156–66. doi: 10.1007/s00125-011-2444-6, 22252473

[30] Huang DQ Wong VWS Rinella ME Boursier J Lazarus JV Yki-Järvinen H . Metabolic dysfunction-associated steatotic liver disease in adults. Nat Rev Dis Primers. (2025) 11:14. doi: 10.1038/s41572-025-00599-1, 40050362

[31] Diambra L Sookoian S Pirola CJ. Proteomic resolution of the MASLD cardiometabolic spectrum identifies sex-driven endotypes and predicts systemic mortality. Gut. (2026):gutjnl-2026-338674. doi: 10.1136/gutjnl-2026-338674, 42527119

[32] Yang D Zhao N Zhang W Peng S Qin X He W. Association between mental health and MASLD molecular insights through metabolomics. Commun Med (Lond). (2025) 6:59. doi: 10.1038/s43856-025-01317-4 PMC12848062 41455836

[33] Xu J Li Y Feng Z Chen H. Cigarette smoke contributes to the progression of MASLD: from the molecular mechanisms to therapy. Cells. (2025) 14:221. doi: 10.3390/cells14030221, 39937012 PMC11816580

[34] Jarvis H O’Keefe H Craig D Stow D Hanratty B Anstee QM. Does moderate alcohol consumption accelerate the progression of liver disease in NAFLD? A systematic review and narrative synthesis. BMJ Open. (2022) 12:e049767. doi: 10.1136/bmjopen-2021-049767 PMC8728442 34983755

[35] Kaneko H Yano Y Okada A Itoh H Suzuki Y Yokota I . Age-dependent association between modifiable risk factors and incident cardiovascular disease. J Am Heart Assoc. (2023) 12:e027684. doi: 10.1161/JAHA.122.027684, 36628975 PMC9939069

[36] Kane AE Gregson E Theou O Rockwood K Howlett SE. The association between frailty, the metabolic syndrome, and mortality over the lifespan. Geroscience. (2017) 39:221–9. doi: 10.1007/s11357-017-9967-9, 28281219 PMC5411368

