# Supplementary material for: Associations of cardiometabolic burden with cardiovascular disease–death transitions in metabolic dysfunction-associated steatotic liver disease: a prospective cohort study using multistate analysis
Source: Front Nutr. 2026 Sep 18;13:1928761. doi: 10.3389/fnut.2026.1928761 (PMC13630770; doi:10.3389/fnut.2026.1928761)
Supplement: Supplementary file 1 [file Supplementary_file_1.docx]

**Supplement**

**Associations of Cardiometabolic Burden With Cardiovascular Disease–Death Transitions in Metabolic Dysfunction-Associated Steatotic Liver Disease:**

**A Prospective Cohort Study Using Multistate Analysis**

Chuanyong Cui, Yi Zhao,Guangji Zhang, Zhongyan Du ,Wenke Cheng

| **Table S1. Alcohol Intake Assessment** | | | |
| --- | --- | --- | --- |
| **Step** | **Question / assessment** | | **Recorded response** |
| 1 | Participants reported how often they drank alcohol using a touchscreen questionnaire. | | Daily or almost daily; 3–4 times a week; 1–2 times a week; monthly or less; Special occasions only; never |
| 2 | Participants who drank alcohol at least weekly were asked to report the average number of glasses consumed per week for each beverage type. | | Average weekly glasses by beverage type |
| 3 | Each reported glass was converted to alcohol units using standard beverage-specific conversion factors. | | Alcohol units per glass |
| 4 | Alcohol units were converted to grams of ethanol. | | 1 unit = 8 g ethanol |
| **Beverage type** | **Units per glass** | **Grams ethanol per glass** | **Calculation** |
| Beer | 2.5 | 20.0 g | 2.5 × 8 |
| White wine | 2.1 | 16.8 g | 2.1 × 8 |
| Fortified wine | 1.0 | 8.0 g | 1.0 × 8 |
| Red wine | 2.1 | 16.8 g | 2.1 × 8 |
| Spirits | 1.0 | 8.0 g | 1.0 × 8 |
| Other alcoholic drinks | 1.5 | 12.0 g | 1.5 × 8 |

Note: Special occasions only” was treated as equivalent to “never” in the assessment.

**Table S2. ICD-9 codes for other liver diseases and alcohol- or drug-related disorders.**

| **Diagnosis** | **ICD-9 codes** |
| --- | --- |
| Alcoholic liver disease | 571.0–571.3 |
| Viral hepatitis | 070.2x–070.6x |
| Autoimmune hepatitis | 571.42 |
| Cholestatic liver diseases | 576.1, 571.6 |
| Chronic hepatitis, unspecified | 571.4x |
| Wilson disease | 275.1 |
| Hemochromatosis | 275.0 |
| Budd–Chiari syndrome | 453.0 |
| Alpha-1-antitrypsin deficiency | 273.4 |
| Secondary or unspecified biliary cirrhosis | 571.6 |
| Alcohol use disorder | 303.xx, 305.0x, 357.5, 425.5, 535.3, 980.0 |
| Drug use disorder | 304.xx, 305.2–305.9 |

ICD-9, International Classification of Diseases, Ninth Revision.

**Table S3. ICD-10 codes for other liver diseases and alcohol- or drug-related disorders**

| **Diagnosis** | **ICD-10 codes** |
| --- | --- |
| Alcoholic liver disease | K70 |
| Viral hepatitis | B16, B17, B18, B19 |
| Autoimmune liver disease | K75.4 |
| Chronic hepatitis, unspecified | K73 |
| Wilson disease | E83.0 |
| Hemochromatosis | E83.1 |
| Budd–Chiari syndrome | I82.0, K76.5 |
| Alpha-1-antitrypsin deficiency | E88.0 |
| Secondary or unspecified biliary cirrhosis | K74.4, K74.5 |
| Alcohol use disorder | F10, E24.4, G62.1, I42.6, K29.2, G31.2, G72.1, K85.2, K86.0, T51.0, T51.9, X65, Z50.2, Z71.4, Z72.1 |
| Drug use disorder | F11, F12, F13, F14, F16, F18, F19 |

ICD-10, International Classification of Diseases, Tenth Revision.

| **Table S4. Diagnostic codes for diseases** | | | | | |
| --- | --- | --- | --- | --- | --- |
| Diseases | ICD-9 diagnosis | ICD-10 diagnosis | Self-report ^a^ | Primary Care ^b^ | Medication ^c^ / Operation code, self-reported ^d^ |
| Hypertension | 401, 4010, 4011, 4019, 402, 4020, 4021, 4029, 403, 4030, 4031, 4039, 404, 4040, 4041, 4049, 405, 4050, 4051, 4059 | I10-I13, I15 | 20002 | Category 3000 | Blood pressure medication / NA |
| Diabetes | 2500, 25000, 25001, 25009, 2501, 25011, 25019, 2503, 2504, 2505, 25099 | E10-E14 | 20002 | Category 3000 | Insulin/ NA |
| CAD | 410, 4109, 411, 4119, 412, 4129, 413, 4139, 4140, 4148, 4149 | I20-I25 | 20002 | Category 3000 | NA / Coronary angioplasty (PTCA) +/- stent; Coronary artery bypass grafts (CABG); Triple heart bypass |
| Stroke | 430-434, 436 | I60-64,Field 40001, Field 40002 | 20002 | Category 3000 | NA |
| Heart failure | 428, 4280, 4281, 4289 | I50 | 20002 | Category 3000 | NA |
| Atrial fibrillation | 4273 | I48 | 20002 | Category 3000 | NA / Pacemaker; Defibrillator insertion; Cardiac ablation |

ICD: International Classification of Disease. CAD, coronary artery disease. NA, not applicable.

^a^ ICD-10 codes in the Hospital inpatient data and Death Register records

^b^ 20002 is the data code used in UK Biobank: Non-cancer illness code, self-reported.

^c^ This category contains data on primary care data recorded by health professionals working at general practices.

^d^ Data-field 6153 and 6177.

^e^ Data-Field 20004

**Table S5. Assessment of hepatic steatosis.**

| **Definition** | **Criteria** |
| --- | --- |
| Hepatic steatosis | Fatty liver index (FLI) ≥60  FLI = 1 / (1 + e^–^*^x^*) × 100;  Where *x* = 0.953 × log_e_ *triglyceride* + 0.139 × *body mass index* + 0.718  × log_e_*γ-glutamyl-transferase* + 0.053 × *waist circumference* − 15.745 |

| **Table S6. Items used to ascertain prior medication use** | | | |
| --- | --- | --- | --- |
| **Glucose-lowering agents** | | | |
| **Field ID 6153/6177 and Data-Field 20003(Data-Coding 4)** | | | |
| **Medication Name** | **Code** | **Medication Name** | **Code** |
| Insulin | 1140883066 | Orabet | 1140874690 |
| Metformin | 1140884600 | Chlorpropamide | 1140874706 |
| Glipizide | 1140874646 | Diabinese | 1140874712 |
| Glibenese | 1140874650 | Glymese | 1140874716 |
| Gliquidone | 1140874658 | Glibenclamide | 1140874718 |
| Glurenorm | 1140874660 | Daonil | 1140874724 |
| Nateglinide | 1141173882 | Acarbose | 1140868902 |
| Tolazamide | 1140874664 | Semi-daonil | 1140874726 |
| Tolanase | 1140874666 | Euglucon | 1140874728 |
| Tolbutamide | 1140874674 | Diabetamide | 1140874736 |
| Glyconon | 1140874678 | Gliclazide | 1140874744 |
| Rastinon | 1140874680 | Diamicron | 1140874746 |
| Glucophage | 1140874686 | Rosiglitazone | 1141177600 |
| Glipizide | 1140874646 | Glipizide | 1140874646 |
| Rosiglitazone + metformin | 1141189090 | Glipizide product | 1141157284 |
| Pioglitazone | 1141171646 | Troglitazone | 1141153254 |
| Glimepiride | 1141152590 |  |  |
|  |  |  |  |
| **Field ID 6153/6177** |  |  |  |
| **Antihypertensive agents** | Do you regularly take any of the following medications?  (You can select more than one answer)   - Cholesterol lowering medication - Blood pressure medication - Insulin - Hormone replacement therapy - Oral contraceptive pill or minipill - None of the above - Do not knowPrefer not to answer | | |
| **Lipid-lowering agents** |  |  |  |
|  |  |  |  |

| **Table S7. Derivation of the weighted cardiometabolic burden score based on component-specific regression coefficients** | | | | | |
| --- | --- | --- | --- | --- | --- |
| **CMRF component** | **Coding** | **coefficient** | **SE** | **P value** | **HR (95% CI)** |
| Hypertension | 0/1 | 0.343 | 0.027 | <0.001 | 1.41 (1.34–1.48) |
| Obesity/central adiposity | 0/1 | 0.230 | 0.092 | 0.012 | 1.26 (1.05–1.51) |
| Low HDL-C or treatment | 0/1 | 0.196 | 0.018 | <0.001 | 1.22 (1.18–1.26) |
| Dysglycaemia or treatment | 0/1 | 0.195 | 0.018 | <0.001 | 1.22 (1.17–1.26) |
| Hypertriglyceridaemia or treatment | 0/1 | -0.041 | 0.022 | 0.056 | 0.96 (0.92–1.00) |

| **Table S8. Schoenfeld residual tests for the primary exposures** | | | |  |
| --- | --- | --- | --- | --- |
| **Model** | **Exposure** | **Outcome** | **Exposure-specific PH test P value** | **Global PH test P value** |
| Traditional Cox | Count-based Cardiometabolic Burden(Per 1 higher count) | CVD | 0.413 | 0.158 |
| Traditional Cox | Weighted Cardiometabolic Burden Score (Per 1 higher SD) | CVD | 0.330 | 0.154 |
| Traditional Cox | Count-based Cardiometabolic Burden(High vs. low) | CVD | 0.661 | 0.245 |
| Traditional Cox | Weighted Cardiometabolic Burden Score (High vs. low) | CVD | 0.154 | 0.098 |
| Traditional Cox | Count-based Cardiometabolic Burden(Per 1 higher count) | Death | 0.427 | 0.216 |
| Traditional Cox | Weighted Cardiometabolic Burden Score (Per 1 higher SD) | Death | 0.201 | 0.174 |
| Traditional Cox | Count-based Cardiometabolic Burden(High vs. low) | Death | 0.350 | 0.138 |
| Traditional Cox | Weighted Cardiometabolic Burden Score (High vs. low) | Death | 0.403 | 0.261 |
| Multistate Cox | Count-based Cardiometabolic Burden(Per 1 higher count) | Baseline to CVD | 0.415 | 0.149 |
| Multistate Cox | Count-based Cardiometabolic Burden(Per 1 higher count) | Baseline to Death | 0.137 | 0.016 |
| Multistate Cox | Count-based Cardiometabolic Burden(Per 1 higher count) | CVD to Death | 0.143 | 0.008 |
| Multistate Cox | Weighted Cardiometabolic Burden Score (Per 1 higher SD) | Baseline to CVD | 0.339 | 0.146 |
| Multistate Cox | Weighted Cardiometabolic Burden Score (Per 1 higher SD) | Baseline to Death | 0.216 | 0.170 |
| Multistate Cox | Weighted Cardiometabolic Burden Score (Per 1 higher SD) | CVD to Death | 0.339 | 0.139 |
| Multistate Cox | Count-based Cardiometabolic Burden(High vs. low) | Baseline to CVD | 0.659 | 0.238 |
| Multistate Cox | Count-based Cardiometabolic Burden(High vs. low) | Baseline to Death | 0.609 | 0.002 |
| Multistate Cox | Count-based Cardiometabolic Burden(High vs. low) | CVD to Death | 0.371 | 0.164 |
| Multistate Cox | Weighted Cardiometabolic Burden Score (High vs. low) | Baseline to CVD | 0.158 | 0.194 |
| Multistate Cox | Weighted Cardiometabolic Burden Score (High vs. low) | Baseline to Death | 0.291 | 0.173 |
| Multistate Cox | Weighted Cardiometabolic Burden Score (High vs. low) | CVD to Death | 0.656 | 0.225 |

**Table S9. Definitions and calculation criteria for inflammatory biomarkers used in the mediation analysis.**

| **Definition** | **Criteria** |
| --- | --- |
| systemic immune-inflammation index (SII) | SII = platelet count × neutrophil count / lymphocyte count |
| inflammatory burden index (IBI) | IBI = C-reactive protein × neutrophil count / lymphocyte count |
| neutrophil-to-lymphocyte ratio (NLR) | NLR = neutrophil count / lymphocyte count |

| **Table S10. The calculation method for cumulative dietary risk** | | |
| --- | --- | --- |
| Variable | Categories reported from the touch-screen questionnaire | Binary Variables |
| Fruit & vegetables (regrouped from fruit, dried fruit & Vegetable) | Serving/day | ≥5 serving/day (Ref.)  <5 serving/day |
| Total fish intake (regrouped from Both total non-oily fish and oily fish) | Never  Less than once a week  Once a week  2-4 times a week  5-6 times a week  Once or more daily | ≥2 times a week (at least once a week of each category) (Ref.)  < once a week of each one |
| Processed meat intake (e.g., ham, bacon, sausages) | Never  Less than once a week  Once a week  2-4 times a week  5-6 times a week  Once or more daily | ≤Once a week (Ref.)  > Once a week |
| Non-processed Red meat (e.g., beef, pork, lamb/mutton) | Never  Less than once a week  Once a week  2-4 times a week  5-6 times a week  Once or more daily | ≤Once a week (Ref.)  >Once a week |
| Milk type used | Full cream  Semi-skimmed  Skimmed  Soya  another type of milk  Never rarely have milk | Semi-skimmed/skimmed (Ref.)  Full cream/ another type of milk/ never rarely have milk |
| Spread type | Never/rarely  Butter  Other type/ margarine  Flora pro-active/benecol | Never/rarely (Ref.)  Another selection |
| Cereal intake | Bowls/week | >5 bowls (Ref)  ≤5 bowls |
| Salt added to food | Never/rarely  Sometimes  Usually  Always | Never/rarely (Ref.)  Another selection |
| Water intake | Glasses/day | ≥6 glasses (Ref.)  <6 glasses |

“Ref.” denotes the reference (healthier) category used to calculate the cumulative dietary risk score. Each non-reference category received 1 point, yielding a total score ranging from 0 (healthiest) to 9 (least healthy).

| **Table S11. Comparison of baseline characteristics between the main MASLD cohort and the MRI-PDFF-defined MASLD subgroup** | | | | |
| --- | --- | --- | --- | --- |
| **Variable** | **Main MASLD cohort (N=72,922)** | **MRI-PDFF-defined MASLD subgroup (N=4,243)** | **P-value** | **SMD** |
| Age(years) | 56.84 (7.89) | 55.19 (7.35) | <0.001 | 0.216 |
| Sex |  |  |  |  |
| Female | 29931 (41.0) | 1767 (41.6) | 0.45 | 0.012 |
| Male | 42991 (59.0) | 2476 (58.4) |  |  |
| Race |  |  |  |  |
| White | 67268 (92.2) | 4055 (95.6) | <0.001 | 0.16 |
| Black | 1586 (2.2) | 28 (0.7) |  |  |
| Asian | 2399 (3.3) | 106 (2.5) |  |  |
| Other | 1669 (2.3) | 54 (1.3) |  |  |
| Smoking status |  |  |  |  |
| Never | 40505 (55.5) | 2713 (63.9) | <0.001 | 0.196 |
| Previous | 25071 (34.4) | 1270 (29.9) |  |  |
| Current | 7011 (9.6) | 254 (6.0) |  |  |
| Missing | 335 (0.5) | 6 (0.1) |  |  |
| IPAQ |  |  |  |  |
| Low | 13239 (18.2) | 820 (19.3) | <0.001 | 0.075 |
| Moderate | 22091 (30.3) | 1506 (35.5) |  |  |
| High | 18760 (25.7) | 1214 (28.6) |  |  |
| Missing | 18832 (25.8) | 703 (16.6) |  |  |
| ALT(U/L) | 25.68 (19.58–34.64) | 24.60 (18.60–34.58) | 0.026 | 0.035 |
| AST(U/L) | 25.9 (22.2–30.8) | 25.50 (21.80–30.20) | 0.007 | 0.043 |
| TDI | -1.06 (3.20) | -1.82 (2.76) | <0.001 | 0.256 |
| Alcohol intake(g/week) | 60.8 (0–120) | 75.20 (0.00–124.00) | <0.001 | 0.147 |
| Count-based Cardiometabolic Burden |  |  |  |  |
| any 1 cardiometabolic factors | 2272 (3.1) | 441 (10.4) | <0.001 | 0.397 |
| any 2 cardiometabolic factors | 13300 (18.2) | 1087 (25.6) |  |  |
| any 3 cardiometabolic factors | 23774 (32.6) | 1275 (30.0) |  |  |
| any 4 cardiometabolic factors | 21528 (29.5) | 1029 (24.3) |  |  |
| any 5 cardiometabolic factors | 12048 (16.5) | 411 (9.7) |  |  |
| Weighted Cardiometabolic Burden Score | 0.64 (0.20) | 0.72 (0.29) | <0.001 | 0.334 |

Data are presented as mean (SD) or median (IQR) for continuous variables according to their distribution, and as number (%) for categorical variables;

IPAQ: International Physical Activity Questionnaire；ALT: alanine aminotransferase; AST: aspartate aminotransferase; ; TDI: Townsend deprivation index

| **Table S12. Associations of cardiometabolic burden with CAD and Death Evaluated by Traditional Cox and Multistate Models.** | | | | | | |
| --- | --- | --- | --- | --- | --- | --- |
|  | **N** | **Events** | **Proportion(%)** | **HR(95%CI)** | **P-value** | **P for FDR** |
| **Traditional Cox model** |  |  |  |  |  |  |
| **Count-based Cardiometabolic Burden(Per 1 higher count)** |  |  |  |  |  |  |
| CAD | 72922 | 9174 | 12.58% | 1.24(1.21-1.27) | <0.001 |  |
| Death | 72922 | 8501 | 11.66% | 1.17(1.14-1.19) | <0.001 |  |
| **Count-based Cardiometabolic Burden(High vs. low)** |  |  |  |  |  |  |
| CAD | 57350 | 7891 | 13.76% | 1.47(1.39-1.56) | <0.001 |  |
| Death | 57350 | 7236 | 12.62% | 1.22(1.15-1.30) | <0.001 |  |
| **Weighted Cardiometabolic Burden Score(Per 1 higher SD)** |  |  |  |  |  |  |
| CAD | 72922 | 9174 | 12.58% | 1.27(1.24-1.30) | <0.001 |  |
| Death | 72922 | 8501 | 11.66% | 1.22(1.19-1.25) | <0.001 |  |
| **Weighted Cardiometabolic Burden Score(High vs. low)** |  |  |  |  |  |  |
| CAD | 31942 | 4968 | 15.55% | 1.41(1.35-1.47) | <0.001 |  |
| Death | 31942 | 4695 | 14.70% | 1.30(1.25-1.36) | <0.001 |  |
| **Multistate model** |  |  |  |  |  |  |
| **Count-based Cardiometabolic Burden(Per 1 higher count)** |  |  |  |  |  |  |
| Baseline to CAD | 72922 | 9174 | 12.58% | 1.24(1.21-1.27) | <0.001 | <0.001 |
| Baseline to Death | 72922 | 6084 | 8.34% | 1.11(1.09-1.14) | <0.001 | <0.001 |
| CAD to Death | 9174 | 2417 | 26.35% | 1.16(1.12-1.21) | <0.001 | <0.001 |
| **Count-based Cardiometabolic Burden(High vs. low)** |  |  |  |  |  |  |
| Baseline to CAD | 57350 | 7891 | 13.76% | 1.47(1.39-1.56) | <0.001 | <0.001 |
| Baseline to Death | 57350 | 5104 | 8.90% | 1.16(1.08-1.24) | <0.001 | <0.001 |
| CAD to Death | 7891 | 2132 | 27.02% | 1.13(0.99-1.28) | 0.062 | 0.062 |
| **Weighted Cardiometabolic Burden Score(Per 1 higher SD)** |  |  |  |  |  |  |
| Baseline to CAD | 72922 | 9174 | 12.58% | 1.27(1.24-1.30) | <0.001 | <0.001 |
| Baseline to Death | 72922 | 6084 | 8.34% | 1.16(1.12-1.19) | <0.001 | <0.001 |
| CAD to Death | 9174 | 2417 | 26.35% | 1.23(1.17-1.29) | <0.001 | <0.001 |
| **Weighted Cardiometabolic Burden Score(High vs. low)** |  |  |  |  |  |  |
| Baseline to CAD | 31942 | 4968 | 15.55% | 1.41(1.35-1.47) | <0.001 | <0.001 |
| Baseline to Death | 31942 | 3204 | 10.03% | 1.22(1.16-1.28) | <0.001 | <0.001 |
| CAD to Death | 4968 | 1491 | 30.01% | 1.27(1.16-1.38) | <0.001 | <0.001 |

The values are hazard ratios (HRs) and their 95% confidence intervals (CIs) from multivariable Cox proportional hazards models and Multistate models. Models were adjusted for age, sex, race, smoking status, alcohol intake, physical activity, TDI, ALT, and AST. Nominal P values are reported alongside false discovery rate (FDR)-adjusted P values, which were calculated using the Benjamini–Hochberg procedure separately for each cardiometabolic burden definition across the three prespecified multistate transitions. For continuous exposure analyses, N represents the total analytic sample; for high-versus-low analyses, N and Events represent the number of participants and events in the high-burden group, respectively, with the low-burden group included as the reference category. Proportion (%) was calculated as Events/N within the corresponding group. CAD: coronary artery disease; ALT: alanine aminotransferase; AST: aspartate aminotransferase; TDI: Townsend deprivation index

| **Table S13. Associations of cardiometabolic burden with Stroke and Death Evaluated by Traditional Cox and Multistate Models.** | | | | | | |
| --- | --- | --- | --- | --- | --- | --- |
|  | **N** | **Events** | **Proportion(%)** | **HR(95%CI)** | **P-value** | **P for FDR** |
| **Traditional Cox model** |  |  |  |  |  |  |
| **Count-based Cardiometabolic Burden(Per 1 higher count)** |  |  |  |  |  |  |
| Stroke | 72922 | 2422 | 3.32% | 1.16(1.11-1.21) | <0.001 |  |
| Death | 72922 | 8501 | 11.66% | 1.17(1.14-1.19) | <0.001 |  |
| **Count-based Cardiometabolic Burden(High vs. low)** |  |  |  |  |  |  |
| Stroke | 57350 | 2080 | 3.63% | 1.37(1.22-1.53) | <0.001 |  |
| Death | 57350 | 7236 | 12.62% | 1.22(1.15-1.30) | <0.001 |  |
| **Weighted Cardiometabolic Burden Score(Per 1 higher SD)** |  |  |  |  |  |  |
| Stroke | 72922 | 2422 | 3.32% | 1.21(1.16-1.27) | <0.001 |  |
| Death | 72922 | 8501 | 11.66% | 1.22(1.19-1.25) | <0.001 |  |
| **Weighted Cardiometabolic Burden Score(High vs. low)** |  |  |  |  |  |  |
| Stroke | 31942 | 1285 | 4.02% | 1.24(1.14-1.34) | <0.001 |  |
| Death | 31942 | 4695 | 14.70% | 1.30(1.25-1.36) | <0.001 |  |
| **Multistate model** |  |  |  |  |  |  |
| **Count-based Cardiometabolic Burden(Per 1 higher count)** |  |  |  |  |  |  |
| Baseline to Stroke | 72922 | 2422 | 3.32% | 1.16(1.11-1.21) | <0.001 | <0.001 |
| Baseline to Death | 72922 | 7589 | 10.41% | 1.16(1.14-1.19) | <0.001 | <0.001 |
| Stroke to Death | 2422 | 912 | 37.65% | 1.10(1.03-1.17) | 0.007 | 0.007 |
| **Count-based Cardiometabolic Burden(High vs. low)** |  |  |  |  |  |  |
| Baseline to Stroke | 57350 | 2080 | 3.63% | 1.37(1.22-1.53) | <0.001 | <0.001 |
| Baseline to Death | 57350 | 6431 | 11.21% | 1.20(1.12-1.28) | <0.001 | <0.001 |
| Stroke to Death | 2080 | 805 | 38.70% | 1.21(0.99-1.48) | 0.068 | 0.068 |
| **Weighted Cardiometabolic Burden Score(Per 1 higher SD)** |  |  |  |  |  |  |
| Baseline to Stroke | 72922 | 2422 | 3.32% | 1.21(1.16-1.27) | <0.001 | <0.001 |
| Baseline to Death | 72922 | 7589 | 10.41% | 1.21(1.18-1.24) | <0.001 | <0.001 |
| Stroke to Death | 2422 | 912 | 37.65% | 1.14(1.06-1.24) | <0.001 | 0.001 |
| **Weighted Cardiometabolic Burden Score(High vs. low)** |  |  |  |  |  |  |
| Baseline to Stroke | 31942 | 1285 | 4.02% | 1.24(1.14-1.34) | <0.001 | <0.001 |
| Baseline to Death | 31942 | 4162 | 13.03% | 1.30(1.24-1.36) | <0.001 | <0.001 |
| Stroke to Death | 1285 | 533 | 41.48% | 1.21(1.05-1.38) | 0.007 | 0.007 |

The values are hazard ratios (HRs) and their 95% confidence intervals (CIs) from multivariable Cox proportional hazards models and Multistate models. Models were adjusted for age, sex, race, smoking status, alcohol intake, physical activity, TDI, ALT, and AST. Nominal P values are reported alongside false discovery rate (FDR)-adjusted P values, which were calculated using the Benjamini–Hochberg procedure separately for each cardiometabolic burden definition across the three prespecified multistate transitions. For continuous exposure analyses, N represents the total analytic sample; for high-versus-low analyses, N and Events represent the number of participants and events in the high-burden group, respectively, with the low-burden group included as the reference category. Proportion (%) was calculated as Events/N within the corresponding group. ALT: alanine aminotransferase; AST: aspartate aminotransferase; TDI: Townsend deprivation index

| **Table S14. Associations of cardiometabolic burden with HF and Death Evaluated by Traditional Cox and Multistate Models.** | | | | | | |
| --- | --- | --- | --- | --- | --- | --- |
|  | **N** | **Events** | **Proportion(%)** | **HR(95%CI)** | **P-value** | **P for FDR** |
| **Traditional Cox model** |  |  |  |  |  |  |
| **Count-based Cardiometabolic Burden(Per 1 higher count)** |  |  |  |  |  |  |
| HF | 72922 | 3475 | 4.77% | 1.25(1.20-1.29) | <0.001 |  |
| Death | 72922 | 8501 | 11.66% | 1.17(1.14-1.19) | <0.001 |  |
| **Count-based Cardiometabolic Burden(High vs. low)** |  |  |  |  |  |  |
| HF | 57350 | 2983 | 5.20% | 1.30(1.18-1.44) | <0.001 |  |
| Death | 57350 | 7236 | 12.62% | 1.22(1.15-1.30) | <0.001 |  |
| **Weighted Cardiometabolic Burden Score(Per 1 higher SD)** |  |  |  |  |  |  |
| HF | 72922 | 3475 | 4.77% | 1.36(1.31-1.41) | <0.001 |  |
| Death | 72922 | 8501 | 11.66% | 1.22(1.19-1.25) | <0.001 |  |
| **Weighted Cardiometabolic Burden Score(High vs. low)** |  |  |  |  |  |  |
| HF | 31942 | 2029 | 6.35% | 1.48(1.39-1.59) | <0.001 |  |
| Death | 31942 | 4695 | 14.70% | 1.30(1.25-1.36) | <0.001 |  |
| **Multistate model** |  |  |  |  |  |  |
| **Count-based Cardiometabolic Burden(Per 1 higher count)** |  |  |  |  |  |  |
| Baseline to HF | 72922 | 3475 | 4.77% | 1.25(1.20-1.29) | <0.001 | <0.001 |
| Baseline to Death | 72922 | 6970 | 9.56% | 1.13(1.11-1.16) | <0.001 | <0.001 |
| HF to Death | 3475 | 1531 | 44.06% | 1.14(1.09-1.20) | <0.001 | <0.001 |
| **Count-based Cardiometabolic Burden(High vs. low)** |  |  |  |  |  |  |
| Baseline to HF | 57350 | 2983 | 5.20% | 1.30(1.18-1.44) | <0.001 | <0.001 |
| Baseline to Death | 57350 | 5893 | 10.28% | 1.19(1.12-1.27) | <0.001 | <0.001 |
| HF to Death | 2983 | 1343 | 45.02% | 1.15(0.99-1.35) | 0.072 | 0.072 |
| **Weighted Cardiometabolic Burden Score(Per 1 higher SD)** |  |  |  |  |  |  |
| Baseline to HF | 72922 | 3475 | 4.77% | 1.36(1.31-1.41) | <0.001 | <0.001 |
| Baseline to Death | 72922 | 6970 | 9.56% | 1.17(1.14-1.20) | <0.001 | <0.001 |
| HF to Death | 3475 | 1531 | 44.06% | 1.18(1.11-1.25) | <0.001 | <0.001 |
| **Weighted Cardiometabolic Burden Score(High vs. low)** |  |  |  |  |  |  |
| Baseline to HF | 31942 | 2029 | 6.35% | 1.48(1.39-1.59) | <0.001 | <0.001 |
| Baseline to Death | 31942 | 3732 | 11.68% | 1.25(1.19-1.31) | <0.001 | <0.001 |
| HF to Death | 2029 | 963 | 47.46% | 1.18(1.06-1.31) | 0.003 | 0.003 |

The values are hazard ratios (HRs) and their 95% confidence intervals (CIs) from multivariable Cox proportional hazards models and Multistate models. Models were adjusted for age, sex, race, smoking status, alcohol intake, physical activity, TDI, ALT, and AST. Nominal P values are reported alongside false discovery rate (FDR)-adjusted P values, which were calculated using the Benjamini–Hochberg procedure separately for each cardiometabolic burden definition across the three prespecified multistate transitions. For continuous exposure analyses, N represents the total analytic sample; for high-versus-low analyses, N and Events represent the number of participants and events in the high-burden group, respectively, with the low-burden group included as the reference category. Proportion (%) was calculated as Events/N within the corresponding group. HF: heart failure; ALT: alanine aminotransferase; AST: aspartate aminotransferase; TDI: Townsend deprivation index

| **Table S15. Associations of cardiometabolic burden with AF and Death Evaluated by Traditional Cox and Multistate Models.** | | | | | | |
| --- | --- | --- | --- | --- | --- | --- |
|  | **N** | **Events** | **Proportion(%)** | **HR(95%CI)** | **P-value** | **P for FDR** |
| **Traditional Cox model** |  |  |  |  |  |  |
| **Count-based Cardiometabolic Burden(Per 1 higher count)** |  |  |  |  |  |  |
| AF | 72922 | 6253 | 8.57% | 1.09(1.07-1.12) | <0.001 |  |
| Death | 72922 | 8501 | 11.66% | 1.17(1.14-1.19) | <0.001 |  |
| **Count-based Cardiometabolic Burden(High vs. low)** |  |  |  |  |  |  |
| AF | 57350 | 5159 | 9.00% | 1.06(0.99-1.13) | 0.076 |  |
| Death | 57350 | 7236 | 12.62% | 1.22(1.15-1.30) | <0.001 |  |
| **Weighted Cardiometabolic Burden Score(Per 1 higher SD)** |  |  |  |  |  |  |
| AF | 72922 | 6253 | 8.57% | 1.18(1.15-1.22) | <0.001 |  |
| Death | 72922 | 8501 | 11.66% | 1.22(1.19-1.25) | <0.001 |  |
| **Weighted Cardiometabolic Burden Score(High vs. low)** |  |  |  |  |  |  |
| AF | 31942 | 3347 | 10.48% | 1.31(1.24-1.38) | <0.001 |  |
| Death | 31942 | 4695 | 14.70% | 1.30(1.25-1.36) | <0.001 |  |
| **Multistate model** |  |  |  |  |  |  |
| **Count-based Cardiometabolic Burden(Per 1 higher count)** |  |  |  |  |  |  |
| Baseline to AF | 72922 | 6253 | 8.57% | 1.10(1.07-1.12) | <0.001 | <0.001 |
| Baseline to Death | 72922 | 6709 | 9.20% | 1.15(1.12-1.17) | <0.001 | <0.001 |
| AF to Death | 6253 | 1792 | 28.66% | 1.19(1.14-1.25) | <0.001 | <0.001 |
| **Count-based Cardiometabolic Burden(High vs. low)** |  |  |  |  |  |  |
| Baseline to AF | 57350 | 5159 | 9.00% | 1.06(0.99-1.13) | 0.074 | 0.074 |
| Baseline to Death | 57350 | 5687 | 9.92% | 1.21(1.13-1.29) | <0.001 | <0.001 |
| AF to Death | 5159 | 1549 | 30.03% | 1.28(1.12-1.47) | <0.001 | <0.001 |
| **Weighted Cardiometabolic Burden Score(Per 1 higher SD)** |  |  |  |  |  |  |
| Baseline to AF | 72922 | 6253 | 8.57% | 1.18(1.15-1.22) | <0.001 | <0.001 |
| Baseline to Death | 72922 | 6709 | 9.20% | 1.19(1.16-1.22) | <0.001 | <0.001 |
| AF to Death | 6253 | 1792 | 28.66% | 1.26(1.19-1.33) | <0.001 | <0.001 |
| **Weighted Cardiometabolic Burden Score(High vs. low)** |  |  |  |  |  |  |
| Baseline to AF | 31942 | 3347 | 10.48% | 1.31(1.24-1.37) | <0.001 | <0.001 |
| Baseline to Death | 31942 | 3586 | 11.23% | 1.25(1.19-1.31) | <0.001 | <0.001 |
| AF to Death | 3347 | 1109 | 33.13% | 1.34(1.21-1.47) | <0.001 | <0.001 |

The values are hazard ratios (HRs) and their 95% confidence intervals (CIs) from multivariable Cox proportional hazards models and Multistate models. Models were adjusted for age, sex, race, smoking status, alcohol intake, physical activity, TDI, ALT, and AST. Nominal P values are reported alongside false discovery rate (FDR)-adjusted P values, which were calculated using the Benjamini–Hochberg procedure separately for each cardiometabolic burden definition across the three prespecified multistate transitions. For continuous exposure analyses, N represents the total analytic sample; for high-versus-low analyses, N and Events represent the number of participants and events in the high-burden group, respectively, with the low-burden group included as the reference category. Proportion (%) was calculated as Events/N within the corresponding group. AF:atrial fibrillation; ALT: alanine aminotransferase; AST: aspartate aminotransferase; TDI: Townsend deprivation index

| **Table S16. Associations of cardiometabolic burden with CVD and Death Evaluated by Traditional Cox and Multistate Models.** | | | | | | | |
| --- | --- | --- | --- | --- | --- | --- | --- |
|  | **N** | **Events** | **Proportion**  **(%)** | **HR(95%CI)** | ***P***  **value** | **P for FDR** | **P for trend** |
| **Traditional Cox model** |  |  |  |  |  |  |  |
| **Count-based Cardiometabolic Burden** |  |  |  |  |  |  |  |
| **CVD** |  |  |  |  |  |  | <0.001 |
| any 2 cardiometabolic factors | 13300 | 2122 | 15.95% | 1.61(1.10-2.35) | 0.015 |  |  |
| any 3 cardiometabolic factors | 23774 | 4398 | 18.50% | 1.93(1.33-2.80) | 0.001 |  |  |
| any 4 cardiometabolic factors | 21528 | 4912 | 22.82% | 2.01(1.39-2.93) | <0.001 |  |  |
| any 5 cardiometabolic factors | 12048 | 3660 | 30.38% | 2.51(1.73-3.65) | <0.001 |  |  |
| **Death** |  |  |  |  |  |  | <0.001 |
| any 2 cardiometabolic factors | 13300 | 1124 | 8.45% | 1.14(0.96-1.36) | 0.136 |  |  |
| any 3 cardiometabolic factors | 23774 | 2247 | 9.45% | 1.18(0.99-1.40) | 0.058 |  |  |
| any 4 cardiometabolic factors | 21528 | 2681 | 12.45% | 1.33(1.13-1.58) | 0.001 |  |  |
| any 5 cardiometabolic factors | 12048 | 2308 | 19.16% | 1.78(1.50-2.11) | <0.001 |  |  |
| **Weighted Cardiometabolic Burden Score** |  |  |  |  |  |  |  |
| **CVD** |  |  |  |  |  |  | <0.001 |
| Quintile 2 | 14991 | 2864 | 19.10% | 1.32 (1.24–1.41) | <0.001 |  |  |
| Quintile 3 | 14537 | 2950 | 20.29% | 1.40 (1.32–1.50) | <0.001 |  |  |
| Quintile 4 | 16997 | 3892 | 22.90% | 1.55 (1.45–1.64) | <0.001 |  |  |
| Quintile 5 | 14945 | 4238 | 28.36% | 1.90 (1.79–2.02) | <0.001 |  |  |
| **Death** |  |  |  |  |  |  | <0.001 |
| Quintile 2 | 14991 | 1361 | 9.08% | 1.03 (0.94–1.13) | 0.484 |  |  |
| Quintile 3 | 14537 | 1655 | 11.38% | 1.25 (1.14–1.36) | <0.001 |  |  |
| Quintile 4 | 16997 | 2085 | 12.27% | 1.26 (1.16–1.36) | <0.001 |  |  |
| Quintile 5 | 14945 | 2610 | 17.46% | 1.67 (1.54–1.81) | <0.001 |  |  |
| **Multistate model** |  |  |  |  |  |  |  |
| **Count-based Cardiometabolic Burden** |  |  |  |  |  |  |  |
| **Baseline to CVD** |  |  |  |  |  |  | <0.001 |
| any 2 cardiometabolic factors | 13300 | 2122 | 15.95% | 1.26(1.11-1.43) | 0.001 | 0.002 |  |
| any 3 cardiometabolic factors | 23774 | 4398 | 18.50% | 1.39(1.23-1.58) | <0.001 | <0.001 |  |
| any 4 cardiometabolic factors | 21528 | 4912 | 22.82% | 1.59(1.40-1.80) | <0.001 | <0.001 |  |
| any 5 cardiometabolic factors | 12048 | 3660 | 30.38% | 2.01(1.77-2.28) | <0.001 | <0.001 |  |
| **Baseline to Death** |  |  |  |  |  |  | <0.001 |
| any 2 cardiometabolic factors | 13300 | 625 | 4.70% | 1.04(0.83-1.29) | 0.749 | 0.749 |  |
| any 3 cardiometabolic factors | 23774 | 1248 | 5.25% | 1.09(0.88-1.35) | 0.438 | 0.508 |  |
| any 4 cardiometabolic factors | 21528 | 1328 | 6.17% | 1.14(0.92-1.41) | 0.222 | 0.332 |  |
| any 5 cardiometabolic factors | 12048 | 981 | 8.14% | 1.38(1.12-1.72) | 0.003 | 0.006 |  |
| **CVD to Death** |  |  |  |  |  |  | <0.001 |
| any 2 cardiometabolic factors | 2121 | 499 | 23.53% | 1.15(0.86-1.53) | 0.358 | 0.477 |  |
| any 3 cardiometabolic factors | 4398 | 999 | 22.71% | 1.11(0.84-1.48) | 0.466 | 0.508 |  |
| any 4 cardiometabolic factors | 4911 | 1353 | 27.55% | 1.24(0.94-1.65) | 0.135 | 0.231 |  |
| any 5 cardiometabolic factors | 3660 | 1327 | 36.26% | 1.58(1.19-2.09) | 0.002 | 0.004 |  |
| **Weighted Cardiometabolic Burden Score** |  |  |  |  |  |  |  |
| **Baseline to CVD** |  |  |  |  |  |  | <0.001 |
| Quintile 2 | 14991 | 2864 | 19.10% | 1.32 (1.24-1.41) | <0.001 | <0.001 |  |
| Quintile 3 | 14537 | 2950 | 20.29% | 1.41 (1.32-1.50) | <0.001 | <0.001 |  |
| Quintile 4 | 16997 | 3892 | 22.90% | 1.55 (1.46-1.65) | <0.001 | <0.001 |  |
| Quintile 5 | 14945 | 4238 | 28.36% | 1.90 (1.79-2.03) | <0.001 | <0.001 |  |
| **Baseline to Death** |  |  |  |  |  |  | <0.001 |
| Quintile 2 | 14991 | 754 | 5.03% | 0.95 (0.85-1.07) | 0.422 | 0.460 |  |
| Quintile 3 | 14537 | 846 | 5.82% | 1.07 (0.96-1.20) | 0.223 | 0.268 |  |
| Quintile 4 | 16997 | 1039 | 6.11% | 1.08(0.97-1.21) | 0.154 | 0.205 |  |
| Quintile 5 | 14945 | 1132 | 7.57% | 1.30 (1.17-1.45) | <0.001 | <0.001 |  |
| **CVD to Death** |  |  |  |  |  |  | 0.043 |
| Quintile 2 | 2864 | 607 | 21.19% | 0.99 (0.86-1.14) | 0.884 | 0.884 |  |
| Quintile 3 | 2950 | 809 | 27.43% | 1.26 (1.10-1.44) | 0.001 | 0.002 |  |
| Quintile 4 | 3892 | 1046 | 26.88% | 1.17 (1.03-1.34) | 0.018 | 0.028 |  |
| Quintile 5 | 4238 | 1478 | 34.87% | 1.51 (1.33-1.72) | <0.001 | <0.001 |  |

The values are hazard ratios (HRs) and their 95% confidence intervals (CIs) from multivariable Cox proportional hazards models and Multistate models. Models were adjusted for age, sex, race, smoking status, alcohol intake, physical activity, TDI, ALT, and AST. Nominal P values are reported alongside false discovery rate (FDR)-adjusted P values, which were calculated using the Benjamini–Hochberg procedure separately for each cardiometabolic burden definition across the three prespecified multistate transitions. For continuous exposure analyses, N represents the total analytic sample; for high-versus-low analyses, N and Events represent the number of participants and events in the high-burden group, respectively, with the low-burden group included as the reference category. Proportion (%) was calculated as Events/N within the corresponding group. CVD: cardiovascular disease; ALT: alanine aminotransferase; AST: aspartate aminotransferase; TDI: Townsend deprivation index

| **Table S17. Associations of cardiometabolic burden with CAD and Death Evaluated by Traditional Cox and Multistate Models.** | | | | | | |  |
| --- | --- | --- | --- | --- | --- | --- | --- |
|  | **N** | **Events** | **Proportion**  **(%)** | **HR(95%CI)** | **P-value** | **P for FDR** | **P for trend** |
| **Traditional Cox model** |  |  |  |  |  |  |  |
| **Count-based Cardiometabolic Burden** |  |  |  |  |  |  |  |
| **CAD** |  |  |  |  |  |  | <0.001 |
| any 2 cardiometabolic factors | 13300 | 1149 | 8.64% | 1.33(1.11-1.59) | 0.002 |  |  |
| any 3 cardiometabolic factors | 23774 | 2565 | 10.79% | 1.59(1.33-1.89) | <0.001 |  |  |
| any 4 cardiometabolic factors | 21528 | 3003 | 13.95% | 1.92(1.62-2.29) | <0.001 |  |  |
| any 5 cardiometabolic factors | 12048 | 2323 | 19.28% | 2.51(2.11-2.99) | <0.001 |  |  |
| **Death** |  |  |  |  |  |  | <0.001 |
| any 2 cardiometabolic factors | 13300 | 1124 | 8.45% | 1.14(0.96-1.36) | 0.136 |  |  |
| any 3 cardiometabolic factors | 23774 | 2247 | 9.45% | 1.18(0.99-1.40) | 0.058 |  |  |
| any 4 cardiometabolic factors | 21528 | 2681 | 12.45% | 1.33(1.13-1.58) | 0.001 |  |  |
| any 5 cardiometabolic factors | 12048 | 2308 | 19.16% | 1.78(1.50-2.11) | <0.001 |  |  |
| **Weighted Cardiometabolic Burden Score** | |  |  |  |  |  |  |
| **CAD** |  |  |  |  |  |  | <0.001 |
| Quintile 2 | 14991 | 1691 | 11.28% | 1.32(1.21–1.43) | <0.001 |  |  |
| Quintile 3 | 14537 | 1667 | 11.47% | 1.34(1.24–1.46) | <0.001 |  |  |
| Quintile 4 | 16997 | 2325 | 13.68% | 1.57(1.45–1.71) | <0.001 |  |  |
| Quintile 5 | 14945 | 2643 | 17.68% | 2.00(1.85–2.16) | <0.001 |  |  |
| **Death** |  |  |  |  |  |  | <0.001 |
| Quintile 2 | 14991 | 1361 | 9.08% | 1.03(0.94–1.13) | 0.484 |  |  |
| Quintile 3 | 14537 | 1655 | 11.38% | 1.25(1.14–1.36) | <0.001 |  |  |
| Quintile 4 | 16997 | 2085 | 12.27% | 1.26(1.16–1.36) | <0.001 |  |  |
| Quintile 5 | 14945 | 2610 | 17.46% | 1.67(1.54–1.81) | <0.001 |  |  |
| **Multistate model** |  |  |  |  |  |  |  |
| **Count-based Cardiometabolic Burden** |  |  |  |  |  |  |  |
| **Baseline to CAD** |  |  |  |  |  |  | <0.001 |
| any 2 cardiometabolic factors | 13300 | 1149 | 8.64% | 1.33(1.11-1.59) | 0.002 | 0.004 |  |
| any 3 cardiometabolic factors | 23774 | 2565 | 10.79% | 1.59(1.33-1.89) | <0.001 | <0.001 |  |
| any 4 cardiometabolic factors | 21528 | 3003 | 13.95% | 1.93(1.62-2.30) | <0.001 | <0.001 |  |
| any 5 cardiometabolic factors | 12048 | 2323 | 19.28% | 2.5(2.11-2.99) | <0.001 | <0.001 |  |
| **Baseline to Death** |  |  |  |  |  |  | <0.001 |
| any 2 cardiometabolic factors | 13300 | 863 | 6.49% | 1.09(0.89-1.32) | 0.411 | 0.487 |  |
| any 3 cardiometabolic factors | 23774 | 1708 | 7.18% | 1.12(0.93-1.36) | 0.222 | 0.334 |  |
| any 4 cardiometabolic factors | 21528 | 1917 | 8.90% | 1.22(1.01-1.47) | 0.036 | 0.062 |  |
| any 5 cardiometabolic factors | 12048 | 1479 | 12.28% | 1.51(1.25-1.82) | <0.001 | <0.001 |  |
| **CAD to Death** |  |  |  |  |  |  | <0.001 |
| any 2 cardiometabolic factors | 1148 | 261 | 22.74% | 1.18(0.77-1.79) | 0.447 | 0.487 |  |
| any 3 cardiometabolic factors | 2565 | 539 | 21.01% | 1.09(0.73-1.64) | 0.675 | 0.675 |  |
| any 4 cardiometabolic factors | 3002 | 764 | 25.45% | 1.21(0.81-1.82) | 0.352 | 0.469 |  |
| any 5 cardiometabolic factors | 2323 | 829 | 35.69% | 1.69(1.13-2.54) | 0.011 | 0.023 |  |
| **Weighted Cardiometabolic Burden Score** | |  |  |  |  |  |  |
| **Baseline to CAD** |  |  |  |  |  |  | <0.001 |
| Quintile 2 | 14991 | 1691 | 11.28% | 1.32(1.22-1.44) | <0.001 | <0.001 |  |
| Quintile 3 | 14537 | 1667 | 11.47% | 1.35(1.24-1.46) | <0.001 | <0.001 |  |
| Quintile 4 | 16997 | 2325 | 13.68% | 1.58(1.46-1.71) | <0.001 | <0.001 |  |
| Quintile 5 | 14945 | 2643 | 17.68% | 2.00(1.85-2.17) | <0.001 | <0.001 |  |
| **Baseline to Death** |  |  |  |  |  |  | <0.001 |
| Quintile 2 | 14991 | 1042 | 6.95% | 1.04(0.94-1.15) | 0.477 | 0.477 |  |
| Quintile 3 | 14537 | 1220 | 8.39% | 1.20(1.09-1.32) | <0.001 | <0.001 |  |
| Quintile 4 | 16997 | 1512 | 8.90% | 1.21(1.10-1.33) | <0.001 | <0.001 |  |
| Quintile 5 | 14945 | 1692 | 11.32% | 1.47(1.34-1.61) | <0.001 | <0.001 |  |
| **CAD to Death** |  |  |  |  |  |  | 0.056 |
| Quintile 2 | 1691 | 319 | 18.86% | 0.87(0.72-1.05) | 0.140 | 0.168 |  |
| Quintile 3 | 1667 | 435 | 26.11% | 1.22(1.02-1.45) | 0.031 | 0.041 |  |
| Quintile 4 | 2325 | 573 | 24.66% | 1.07(0.90-1.27) | 0.444 | 0.477 |  |
| Quintile 5 | 2643 | 918 | 34.73% | 1.55(1.32-1.83) | <0.001 | <0.001 |  |

The values are hazard ratios (HRs) and their 95% confidence intervals (CIs) from multivariable Cox proportional hazards models and Multistate models. Models were adjusted for age, sex, race, smoking status, alcohol intake, physical activity, TDI, ALT, and AST. Nominal P values are reported alongside false discovery rate (FDR)-adjusted P values, which were calculated using the Benjamini–Hochberg procedure separately for each cardiometabolic burden definition across the three prespecified multistate transitions. For continuous exposure analyses, N represents the total analytic sample; for high-versus-low analyses, N and Events represent the number of participants and events in the high-burden group, respectively, with the low-burden group included as the reference category. Proportion (%) was calculated as Events/N within the corresponding group. CAD: coronary artery disease; ALT: alanine aminotransferase; AST: aspartate aminotransferase; TDI: Townsend deprivation index

| **Table S18. Associations of cardiometabolic burden with stroke and Death Evaluated by Traditional Cox and Multistate Models.** | | | | | | |  |
| --- | --- | --- | --- | --- | --- | --- | --- |
|  | **N** | **Events** | **Proportion**  **(%)** | **HR(95%CI)** | **P-value** | **P for FDR** | **P for trend** |
| **Traditional Cox model** |  |  |  |  |  |  |  |
| **Count-based Cardiometabolic Burden** |  |  |  |  |  |  |  |
| **Stroke** |  |  |  |  |  |  | <0.001 |
| any 2 cardiometabolic factors | 13300 | 313 | 2.35% | 1.61(1.09-2.35) | 0.015 |  |  |
| any 3 cardiometabolic factors | 23774 | 717 | 3.02% | 1.93(1.33-2.80) | 0.001 |  |  |
| any 4 cardiometabolic factors | 21528 | 766 | 3.56% | 2.02(1.39-2.93) | <0.001 |  |  |
| any 5 cardiometabolic factors | 12048 | 597 | 4.96% | 2.51(1.73-3.65) | <0.001 |  |  |
| **Death** |  |  |  |  |  |  | <0.001 |
| any 2 cardiometabolic factors | 13300 | 1124 | 8.45% | 1.14(0.96-1.36) | 0.136 |  |  |
| any 3 cardiometabolic factors | 23774 | 2247 | 9.45% | 1.18(0.99-1.40) | 0.058 |  |  |
| any 4 cardiometabolic factors | 21528 | 2681 | 12.45% | 1.33(1.13-1.58) | 0.001 |  |  |
| any 5 cardiometabolic factors | 12048 | 2308 | 19.16% | 1.78(1.50-2.11) | <0.001 |  |  |
| **Weighted Cardiometabolic Burden Score** | |  |  |  |  |  |  |
| **Stroke** |  |  |  |  |  |  | <0.001 |
| Quintile 2 | 14991 | 469 | 3.13% | 1.60 (1.35–1.90) | <0.001 |  |  |
| Quintile 3 | 14537 | 484 | 3.33% | 1.64 (1.38–1.95) | <0.001 |  |  |
| Quintile 4 | 16997 | 593 | 3.49% | 1.64 (1.38–1.93) | <0.001 |  |  |
| Quintile 5 | 14945 | 692 | 4.63% | 2.06 (1.74–2.43) | <0.001 |  |  |
| **Death** |  |  |  |  |  |  | <0.001 |
| Quintile 2 | 14991 | 1361 | 9.08% | 1.03(0.94–1.13) | 0.484 |  |  |
| Quintile 3 | 14537 | 1655 | 11.38% | 1.25(1.14–1.36) | <0.001 |  |  |
| Quintile 4 | 16997 | 2085 | 12.27% | 1.26(1.16–1.36) | <0.001 |  |  |
| Quintile 5 | 14945 | 2610 | 17.46% | 1.67(1.54–1.81) | <0.001 |  |  |
| **Multistate model** |  |  |  |  |  |  |  |
| **Count-based Cardiometabolic Burden** |  |  |  |  |  |  |  |
| **Baseline to Stroke** |  |  |  |  |  |  | <0.001 |
| any 2 cardiometabolic factors | 13300 | 313 | 2.35% | 1.61(1.10-2.35) | 0.015 | 0.030 |  |
| any 3 cardiometabolic factors | 23774 | 717 | 3.02% | 1.93(1.33-2.80) | 0.001 | 0.002 |  |
| any 4 cardiometabolic factors | 21528 | 766 | 3.56% | 2.02(1.39-2.93) | <0.001 | 0.001 |  |
| any 5 cardiometabolic factors | 12048 | 597 | 4.96% | 2.51(1.73-3.65) | <0.001 | <0.001 |  |
| **Baseline to Death** |  |  |  |  |  |  | <0.001 |
| any 2 cardiometabolic factors | 13300 | 1026 | 7.71% | 1.13(0.94-1.35) | 0.190 | 0.284 |  |
| any 3 cardiometabolic factors | 23774 | 2006 | 8.44% | 1.14(0.96-1.36) | 0.137 | 0.235 |  |
| any 4 cardiometabolic factors | 21528 | 2381 | 11.06% | 1.30(1.08-1.54) | 0.004 | 0.010 |  |
| any 5 cardiometabolic factors | 12048 | 2044 | 16.97% | 1.74(1.46-2.08) | <0.001 | <0.001 |  |
| **Stroke to Death** |  |  |  |  |  |  | <0.001 |
| any 2 cardiometabolic factors | 313 | 98 | 31.31% | 0.70(0.35-1.34) | 0.315 | 0.420 |  |
| any 3 cardiometabolic factors | 717 | 241 | 33.61% | 0.81(0.41-1.58) | 0.536 | 0.644 |  |
| any 4 cardiometabolic factors | 766 | 300 | 39.16% | 0.86(0.44-1.68) | 0.655 | 0.714 |  |
| any 5 cardiometabolic factors | 597 | 264 | 44.22% | 0.98(0.50-1.92) | 0.956 | 0.956 |  |
| **Weighted Cardiometabolic Burden Score** | |  |  |  |  |  |  |
| **Baseline to Stroke** |  |  |  |  |  |  | <0.001 |
| Quintile 2 | 14991 | 469 | 3.13% | 1.60(1.35-1.90) | <0.001 | <0.001 |  |
| Quintile 3 | 14537 | 484 | 3.33% | 1.65(1.39-1.95) | <0.001 | <0.001 |  |
| Quintile 4 | 16997 | 593 | 3.49% | 1.64(1.39-1.94) | <0.001 | <0.001 |  |
| Quintile 5 | 14945 | 692 | 4.63% | 2.06(1.74-2.43) | <0.001 | <0.001 |  |
| **Baseline to Death** |  |  |  |  |  |  | <0.001 |
| Quintile 2 | 14991 | 1210 | 8.07% | 0.99(0.91-1.10) | 0.988 | 0.988 |  |
| Quintile 3 | 14537 | 1482 | 10.19% | 1.22(1.12-1.34) | <0.001 | <0.001 |  |
| Quintile 4 | 16997 | 1853 | 10.90% | 1.22(1.12-1.33) | <0.001 | <0.001 |  |
| Quintile 5 | 14945 | 2309 | 15.45% | 1.63(1.50-1.77) | <0.001 | <0.001 |  |
| **Stroke to Death** |  |  |  |  |  |  | <0.001 |
| Quintile 2 | 469 | 151 | 32.20% | 0.96(0.70-1.31) | 0.792 | 0.951 |  |
| Quintile 3 | 484 | 173 | 35.74% | 1.01(0.74-1.38) | 0.934 | 0.988 |  |
| Quintile 4 | 593 | 232 | 39.12% | 1.12(0.83-1.51) | 0.461 | 0.614 |  |
| Quintile 5 | 692 | 301 | 43.50% | 1.26(0.94-1.69) | 0.119 | 0.179 |  |

The values are hazard ratios (HRs) and their 95% confidence intervals (CIs) from multivariable Cox proportional hazards models and Multistate models. Models were adjusted for age, sex, race, smoking status, alcohol intake, physical activity, TDI, ALT, and AST. Nominal P values are reported alongside false discovery rate (FDR)-adjusted P values, which were calculated using the Benjamini–Hochberg procedure separately for each cardiometabolic burden definition across the three prespecified multistate transitions. For continuous exposure analyses, N represents the total analytic sample; for high-versus-low analyses, N and Events represent the number of participants and events in the high-burden group, respectively, with the low-burden group included as the reference category. Proportion (%) was calculated as Events/N within the corresponding group. ALT: alanine aminotransferase; AST: aspartate aminotransferase; TDI: Townsend deprivation index

| **Table S19. Associations of cardiometabolic burden with HF and Death Evaluated by Traditional Cox and Multistate Models.** | | | | | | |  |
| --- | --- | --- | --- | --- | --- | --- | --- |
|  | **N** | **Events** | **Proportion**  **(%)** | **HR(95%CI)** | **P-value** | **P for FDR** | **P for trend** |
| **Traditional Cox model** |  |  |  |  |  |  |  |
| **Count-based Cardiometabolic Burden** |  |  |  |  |  |  |  |
| **HF** |  |  |  |  |  |  | <0.001 |
| any 2 cardiometabolic factors | 13300 | 450 | 3.38% | 1.56(1.13-2.13) | 0.006 |  |  |
| any 3 cardiometabolic factors | 23774 | 863 | 3.63% | 1.55(1.14-2.12) | 0.005 |  |  |
| any 4 cardiometabolic factors | 21528 | 1080 | 5.02% | 1.86(1.36-2.53) | <0.001 |  |  |
| any 5 cardiometabolic factors | 12048 | 1040 | 8.63% | 2.78(2.04-3.78) | <0.001 |  |  |
| **Death** |  |  |  |  |  |  | <0.001 |
| any 2 cardiometabolic factors | 13300 | 1124 | 8.45% | 1.14(0.96-1.36) | 0.136 |  |  |
| any 3 cardiometabolic factors | 23774 | 2247 | 9.45% | 1.18(0.99-1.40) | 0.058 |  |  |
| any 4 cardiometabolic factors | 21528 | 2681 | 12.45% | 1.33(1.13-1.58) | 0.001 |  |  |
| any 5 cardiometabolic factors | 12048 | 2308 | 19.16% | 1.78(1.50-2.11) | <0.001 |  |  |
| **Weighted Cardiometabolic Burden Score** | |  |  |  |  |  |  |
| **HF** |  |  |  |  |  |  | <0.001 |
| Quintile 2 | 14991 | 516 | 3.44% | 1.27 (1.09–1.48) | 0.002 |  |  |
| Quintile 3 | 14537 | 679 | 4.67% | 1.64 (1.42–1.90) | <0.001 |  |  |
| Quintile 4 | 16997 | 833 | 4.90% | 1.62 (1.40–1.87) | <0.001 |  |  |
| Quintile 5 | 14945 | 1196 | 8.00% | 2.46 (2.14–2.83) | <0.001 |  |  |
| **Death** |  |  |  |  |  |  | <0.001 |
| Quintile 2 | 14991 | 1361 | 9.08% | 1.03(0.94–1.13) | 0.484 |  |  |
| Quintile 3 | 14537 | 1655 | 11.38% | 1.25(1.14–1.36) | <0.001 |  |  |
| Quintile 4 | 16997 | 2085 | 12.27% | 1.26(1.16–1.36) | <0.001 |  |  |
| Quintile 5 | 14945 | 2610 | 17.46% | 1.67(1.54–1.81) | <0.001 |  |  |
| **Multistate model** |  |  |  |  |  |  |  |
| **Count-based Cardiometabolic Burden** |  |  |  |  |  |  |  |
| **Baseline to HF** |  |  |  |  |  |  | <0.001 |
| any 2 cardiometabolic factors | 13300 | 450 | 3.38% | 1.56(1.13-2.14) | 0.006 | 0.015 |  |
| any 3 cardiometabolic factors | 23774 | 863 | 3.63% | 1.55(1.14-2.12) | 0.005 | 0.015 |  |
| any 4 cardiometabolic factors | 21528 | 1080 | 5.02% | 1.86(1.37-2.53) | <0.001 | <0.001 |  |
| any 5 cardiometabolic factors | 12048 | 1040 | 8.63% | 2.78(2.04-3.79) | <0.001 | <0.001 |  |
| **Baseline to Death** |  |  |  |  |  |  | <0.001 |
| any 2 cardiometabolic factors | 13300 | 949 | 7.14% | 1.08(0.89-1.29) | 0.443 | 0.443 |  |
| any 3 cardiometabolic factors | 23774 | 1930 | 8.12% | 1.13(0.95-1.35) | 0.178 | 0.226 |  |
| any 4 cardiometabolic factors | 21528 | 2214 | 10.28% | 1.25(1.04-1.49) | 0.015 | 0.026 |  |
| any 5 cardiometabolic factors | 12048 | 1749 | 14.52% | 1.56(1.30-1.87) | <0.001 | <0.001 |  |
| **HF to Death** |  |  |  |  |  |  | <0.001 |
| any 2 cardiometabolic factors | 449 | 175 | 38.98% | 1.46(0.83-2.57) | 0.189 | 0.226 |  |
| any 3 cardiometabolic factors | 863 | 317 | 36.73% | 1.38(0.79-2.40) | 0.260 | 0.284 |  |
| any 4 cardiometabolic factors | 1080 | 467 | 43.24% | 1.55(0.89-2.69) | 0.123 | 0.185 |  |
| any 5 cardiometabolic factors | 1040 | 559 | 53.75% | 1.99(1.14-3.46) | 0.015 | 0.026 |  |
| **Weighted Cardiometabolic Burden Score** | |  |  |  |  |  |  |
| **Baseline to HF** |  |  |  |  |  |  | <0.001 |
| Quintile 2 | 14991 | 516 | 3.44% | 1.27(1.09-1.48) | 0.002 | 0.003 |  |
| Quintile 3 | 14537 | 679 | 4.67% | 1.64 (1.42-1.90) | <0.001 | <0.001 |  |
| Quintile 4 | 16997 | 833 | 4.90% | 1.62(1.41-1.87) | <0.001 | <0.001 |  |
| Quintile 5 | 14945 | 1196 | 8.00% | 2.47(2.15-2.83) | <0.001 | <0.001 |  |
| **Baseline to Death** |  |  |  |  |  |  | <0.001 |
| Quintile 2 | 14991 | 1176 | 7.84% | 1.01(0.92-1.11) | 0.859 | 0.918 |  |
| Quintile 3 | 14537 | 1357 | 9.33% | 1.17(1.07-1.28) | 0.001 | 0.002 |  |
| Quintile 4 | 16997 | 1741 | 10.24% | 1.21(1.10-1.32) | <0.001 | <0.001 |  |
| Quintile 5 | 14945 | 1991 | 13.32% | 1.49(1.37-1.63) | <0.001 | <0.001 |  |
| **HF to Death** |  |  |  |  |  |  | 0.037 |
| Quintile 2 | 516 | 185 | 35.85% | 0.99(0.76-1.28) | 0.918 | 0.918 |  |
| Quintile 3 | 679 | 298 | 43.89% | 1.24 (0.97-1.58) | 0.082 | 0.109 |  |
| Quintile 4 | 833 | 344 | 41.30% | 1.11(0.87-1.41) | 0.397 | 0.476 |  |
| Quintile 5 | 1196 | 619 | 51.76% | 1.46 (1.16-1.84) | 0.001 | 0.002 |  |

The values are hazard ratios (HRs) and their 95% confidence intervals (CIs) from multivariable Cox proportional hazards models and Multistate models. Models were adjusted for age, sex, race, smoking status, alcohol intake, physical activity, TDI, ALT, and AST. Nominal P values are reported alongside false discovery rate (FDR)-adjusted P values, which were calculated using the Benjamini–Hochberg procedure separately for each cardiometabolic burden definition across the three prespecified multistate transitions. For continuous exposure analyses, N represents the total analytic sample; for high-versus-low analyses, N and Events represent the number of participants and events in the high-burden group, respectively, with the low-burden group included as the reference category. Proportion (%) was calculated as Events/N within the corresponding group. HF: heart failure ALT: alanine aminotransferase; AST: aspartate aminotransferase; TDI: Townsend deprivation index

| **Table S20. Associations of cardiometabolic burden with AF and Death Evaluated by Traditional Cox and Multistate Models.** | | | | | | |  |
| --- | --- | --- | --- | --- | --- | --- | --- |
|  | **N** | **Events** | **Proportion(%)** | **HR(95%CI)** | **P-value** | **P for FDR** | **P for trend** |
| **Traditional Cox model** |  |  |  |  |  |  |  |
| **Count-based Cardiometabolic Burden** | |  |  |  |  |  |  |
| **AF** |  |  |  |  |  |  | <0.001 |
| any 2 cardiometabolic factors | 13300 | 963 | 7.24% | 1.06(0.88-1.27) | 0.568 |  |  |
| any 3 cardiometabolic factors | 23774 | 1747 | 7.35% | 1(0.84-1.19) | 0.998 |  |  |
| any 4 cardiometabolic factors | 21528 | 1940 | 9.01% | 1.09(0.92-1.31) | 0.327 |  |  |
| any 5 cardiometabolic factors | 12048 | 1472 | 12.22% | 1.37(1.15-1.64) | 0.001 |  |  |
| **Death** |  |  |  |  |  |  | <0.001 |
| any 2 cardiometabolic factors | 13300 | 1124 | 8.45% | 1.14(0.96-1.36) | 0.136 |  |  |
| any 3 cardiometabolic factors | 23774 | 2247 | 9.45% | 1.18(0.99-1.40) | 0.058 |  |  |
| any 4 cardiometabolic factors | 21528 | 2681 | 12.45% | 1.33(1.13-1.58) | 0.001 |  |  |
| any 5 cardiometabolic factors | 12048 | 2308 | 19.16% | 1.78(1.50-2.11) | <0.001 |  |  |
| **Weighted Cardiometabolic Burden Score** | |  |  |  |  |  |  |
| **AF** |  |  |  |  |  |  | <0.001 |
| Quintile 2 | 14991 | 1125 | 7.50% | 1.20 (1.08–1.32) | <0.001 |  |  |
| Quintile 3 | 14537 | 1235 | 8.50% | 1.37 (1.24–1.51) | <0.001 |  |  |
| Quintile 4 | 16997 | 1631 | 9.60% | 1.47 (1.33–1.62) | <0.001 |  |  |
| Quintile 5 | 14945 | 1716 | 11.48% | 1.75 (1.58–1.93) | <0.001 |  |  |
| **Death** |  |  |  |  |  |  | <0.001 |
| Quintile 2 | 14991 | 1361 | 9.08% | 1.03(0.94–1.13) | 0.484 |  |  |
| Quintile 3 | 14537 | 1655 | 11.38% | 1.25(1.14–1.36) | <0.001 |  |  |
| Quintile 4 | 16997 | 2085 | 12.27% | 1.26(1.16–1.36) | <0.001 |  |  |
| Quintile 5 | 14945 | 2610 | 17.46% | 1.67(1.54–1.81) | <0.001 |  |  |
| **Multistate model** |  |  |  |  |  |  |  |
| **Count-based Cardiometabolic Burden** |  |  |  |  |  |  |  |
| **Baseline to AF** |  |  |  |  |  |  | <0.001 |
| any 2 cardiometabolic factors | 13300 | 963 | 7.24% | 1.05(0.88-1.27) | 0.573 | 0.625 |  |
| any 3 cardiometabolic factors | 23774 | 1747 | 7.35% | 1(0.84-1.19) | 0.997 | 0.997 |  |
| any 4 cardiometabolic factors | 21528 | 1940 | 9.01% | 1.09(0.92-1.31) | 0.327 | 0.392 |  |
| any 5 cardiometabolic factors | 12048 | 1472 | 12.22% | 1.37(1.15-1.64) | 0.001 | 0.002 |  |
| **Baseline to Death** |  |  |  |  |  |  | <0.001 |
| any 2 cardiometabolic factors | 13300 | 904 | 6.80% | 1.12(0.92-1.35) | 0.267 | 0.355 |  |
| any 3 cardiometabolic factors | 23774 | 1841 | 7.74% | 1.17(0.97-1.41) | 0.097 | 0.194 |  |
| any 4 cardiometabolic factors | 21528 | 2120 | 9.85% | 1.3(1.08-1.57) | 0.006 | 0.017 |  |
| any 5 cardiometabolic factors | 12048 | 1726 | 14.33% | 1.68(1.39-2.02) | <0.001 | <0.001 |  |
| **AF to Death** |  |  |  |  |  |  | <0.001 |
| any 2 cardiometabolic factors | 961 | 219 | 22.79% | 1.33(0.86-2.04) | 0.198 | 0.297 |  |
| any 3 cardiometabolic factors | 1744 | 403 | 23.11% | 1.34(0.88-2.05) | 0.169 | 0.290 |  |
| any 4 cardiometabolic factors | 1936 | 557 | 28.77% | 1.58(1.04-2.41) | 0.032 | 0.076 |  |
| any 5 cardiometabolic factors | 1468 | 578 | 39.37% | 2.11(1.39-3.21) | 0.001 | 0.002 |  |
| **Weighted Cardiometabolic Burden Score** | |  |  |  |  |  |  |
| **Baseline to AF** |  |  |  |  |  |  | <0.001 |
| Quintile 2 | 14991 | 1125 | 7.50% | 1.2 (1.08-1.32) | <0.001 | <0.001 |  |
| Quintile 3 | 14537 | 1235 | 8.50% | 1.37(1.24-1.52) | <0.001 | <0.001 |  |
| Quintile 4 | 16997 | 1631 | 9.60% | 1.47(1.33-1.62) | <0.001 | <0.001 |  |
| Quintile 5 | 14945 | 1716 | 11.48% | 1.75(1.59-1.93) | <0.001 | <0.001 |  |
| **Baseline to Death** |  |  |  |  |  |  | <0.001 |
| Quintile 2 | 14991 | 1121 | 7.48% | 1.02(0.92-1.12) | 0.77 | 0.77 |  |
| Quintile 3 | 14537 | 1322 | 9.09% | 1.20(1.09-1.32) | <0.001 | <0.001 |  |
| Quintile 4 | 16997 | 1621 | 9.54% | 1.19(1.09-1.30) | <0.001 | <0.001 |  |
| Quintile 5 | 14945 | 1965 | 13.15% | 1.55(1.41-1.69) | <0.001 | <0.001 |  |
| **AF to Death** |  |  |  |  |  |  | <0.001 |
| Quintile 2 | 1125 | 240 | 21.33% | 1.09(0.87-1.36) | 0.473 | 0.516 |  |
| Quintile 3 | 1235 | 333 | 26.96% | 1.31(1.06-1.63) | 0.014 | 0.017 |  |
| Quintile 4 | 1631 | 464 | 28.45% | 1.34(1.08-1.65) | 0.007 | 0.009 |  |
| Quintile 5 | 1716 | 645 | 37.59% | 1.79 (1.46-2.2) | <0.001 | <0.001 |  |

The values are hazard ratios (HRs) and their 95% confidence intervals (CIs) from multivariable Cox proportional hazards models and Multistate models. Models were adjusted for age, sex, race, smoking status, alcohol intake, physical activity, TDI, ALT, and AST. Nominal P values are reported alongside false discovery rate (FDR)-adjusted P values, which were calculated using the Benjamini–Hochberg procedure separately for each cardiometabolic burden definition across the three prespecified multistate transitions. For continuous exposure analyses, N represents the total analytic sample; for high-versus-low analyses, N and Events represent the number of participants and events in the high-burden group, respectively, with the low-burden group included as the reference category. Proportion (%) was calculated as Events/N within the corresponding group. AF:atrial fibrillation; ALT: alanine aminotransferase; AST: aspartate aminotransferase; TDI: Townsend deprivation index.

| **Table S21. Transfer probability matrix for the three states of participants exposed to high and low cardiometabolic burden.** | | | | | |
| --- | --- | --- | --- | --- | --- |
|  | **Count-based Cardiometabolic Burden** | | | **Weighted Cardiometabolic Burden Score** | |
|  | **High(57,350)** | | **Low(n=15,572)** | **High(n=31,942)** | **Low(n=40,980)** |
| **1-year follow up** |  |  | |  |  |
| Baseline | 99.24% | 99.4% | | 99.18% | 99.36% |
| CVD | 0.70% | 0.56% | | 0.76% | 0.59% |
| Death | 0.06% | 0.05% | | 0.07% | 0.05% |
| **3-year follow up** |  |  | |  |  |
| Baseline | 97.40% | 97.93% | | 97.18% | 97.80% |
| CVD | 2.29% | 1.83% | | 2.48% | 1.94% |
| Death | 0.31% | 0.24% | | 0.34% | 0.26% |
| **5-year follow up** |  |  | |  |  |
| Baseline | 95.17% | 96.15% | | 94.76% | 95.91% |
| CVD | 4.09% | 3.28% | | 4.43% | 3.48% |
| Death | 0.74% | 0.57% | | 0.81% | 0.61% |
| **10-year follow up** |  |  | |  |  |
| Baseline | 87.98% | 90.34% | | 87.00% | 89.75% |
| CVD | 9.45% | 7.69% | | 10.18% | 8.14% |
| Death | 2.56% | 1.97% | | 2.82% | 2.11% |
| **15-year follow up** |  |  | |  |  |
| Baseline | 80.06% | 83.82% | | 78.50% | 82.87% |
| CVD | 14.40% | 11.91% | | 15.42% | 12.56% |
| Death | 5.54% | 4.27% | | 6.08% | 4.57% |

Adjusted transition probabilities were derived from a three-state multistate Cox model including baseline, CVD, and death states. State occupation probabilities were numerically estimated using transition-specific baseline cumulative hazards and covariate-adjusted risk estimates, and were extracted at 1, 3, 5, 10, and 15 years.

Models were adjusted for age, sex, race, smoking status, alcohol intake, physical activity, TDI, ALT, and AST.

CVD: cardiovascular disease ; ALT: alanine aminotransferase; AST: aspartate aminotransferase; TDI: Townsend deprivation index

| **Table S22. Mediated role of CRP in associations of cardiometabolic burden with CVD and death transitions in multistate models** | | | | | |
| --- | --- | --- | --- | --- | --- |
| **Transitions** | **Effect Type** | **Effect Value** | **P-Value for effect** | **Mediated proportion (%)** | **P-Value for mediated proportion** |
| **Count-based Cardiometabolic Burden** |  |  |  |  |  |
| **Baseline to CVD** | Total | 1.1727 (1.1590-1.1906) | <0.001 |  |  |
|  | Direct | 1.1717 (1.1580-1.1894) | <0.001 |  |  |
|  | Indirect | 1.0009 (1.0004-1.0015) | 0.005 | 0.54 (0.23-0.95) | 0.005 |
| **Baseline to Death** | Total | 1.0943 (1.0636-1.1320) | <0.001 |  |  |
|  | Direct | 1.0929 (1.0623-1.1306) | <0.001 |  |  |
|  | Indirect | 1.0013 (1.0004-1.0022) | 0.004 | 1.39 (0.47-2.63) | 0.015 |
| **CVD to Death** | Total | 1.1258 (1.0922-1.1653) | <0.001 |  |  |
|  | Direct | 1.1243 (1.0914-1.1649) | <0.001 |  |  |
|  | Indirect | 1.0013 (0.9998-1.0027) | 0.094 | 1.10 (-0.18-2.32) | 0.106 |
| **Weighted Cardiometabolic Burden Score** |  |  |  |  |  |
| **Baseline to CVD** | Total | 1.2268 (1.2072-1.2457) | <0.001 |  |  |
|  | Direct | 1.2234 (1.2048-1.2417) | <0.001 |  |  |
|  | Indirect | 1.0028 (1.0019-1.0036) | <0.001 | 1.34 (0.96-1.73) | <0.001 |
| **Baseline to Death** | Total | 1.1209 (1.0867-1.1581) | <0.001 |  |  |
|  | Direct | 1.1161 (1.0827-1.1533) | <0.001 |  |  |
|  | Indirect | 1.0043 (1.0030-1.0059) | <0.001 | 3.76 (2.46-5.55) | <0.001 |
| **CVD to Death** | Total | 1.1766 (1.1411-1.2161) | <0.001 |  |  |
|  | Direct | 1.1715 (1.1372-1.2127) | <0.001 |  |  |
|  | Indirect | 1.0044 (1.0022-1.0068) | <0.001 | 2.68 (1.36-4.43) | 0.001 |

Direct, indirect, and total effects and the proportion mediated were estimated using g-computation within the multistate framework, with transition-specific Cox models used for each disease-state transition. 95% CIs were derived from 2,000 bootstrap replications.

Models were adjusted for age, sex, race, smoking status, alcohol intake, physical activity, TDI, ALT, and AST.

CVD: cardiovascular disease ; ALT: alanine aminotransferase; AST: aspartate aminotransferase; TDI: Townsend deprivation index

| **Table S23. Mediated role of SII in associations of cardiometabolic burden with CVD and death transitions in multistate models** | | | | | |
| --- | --- | --- | --- | --- | --- |
| **Transitions** | **Effect Type** | **Effect Value** | **P-Value for effect** | **Mediated proportion (%)** | **P-Value for mediated proportion** |
| **Count-based Cardiometabolic Burden** |  |  |  |  |  |
| **Baseline to CVD** | Total | 1.1710 (1.1560-1.1907) | <0.001 |  |  |
|  | Direct | 1.1697 (1.1548-1.1896) | <0.001 |  |  |
|  | Indirect | 1.0011 (1.0007-1.0018) | <0.001 | 0.70 (0.48-1.14) | <0.001 |
| **Baseline to Death** | Total | 1.0960 (1.0646-1.1349) | <0.001 |  |  |
|  | Direct | 1.0945 (1.0635-1.1326) | <0.001 |  |  |
|  | Indirect | 1.0014 (1.0008-1.0023) | <0.001 | 1.53 (0.85-2.84) | 0.004 |
| **CVD to Death** | Total | 1.1233 (1.0944-1.1667) | <0.001 |  |  |
|  | Direct | 1.1206 (1.0920-1.1645) | <0.001 |  |  |
|  | Indirect | 1.0024 (1.0011-1.0040) | 0.002 | 2.06 (0.80-3.67) | 0.007 |
| **Weighted Cardiometabolic Burden Score** |  |  |  |  |  |
| **Baseline to CVD** | Total | 1.2246 (1.2014-1.2468) | <0.001 |  |  |
|  | Direct | 1.2216 (1.1982-1.2428) | <0.001 |  |  |
|  | Indirect | 1.0024 (1.0019-1.0035) | <0.001 | 1.20 (0.92-1.70) | <0.001 |
| **Baseline to Death** | Total | 1.1189 (1.0864-1.1538) | <0.001 |  |  |
|  | Direct | 1.1154 (1.0824-1.1505) | <0.001 |  |  |
|  | Indirect | 1.0031 (1.0022-1.0048) | <0.001 | 2.77 (1.73-5.17) | 0.003 |
| **CVD to Death** | Total | 1.1668 (1.1244-1.2097) | <0.001 |  |  |
|  | Direct | 1.1620 (1.1191-1.2056) | <0.001 |  |  |
|  | Indirect | 1.0042 (1.0032-1.0067) | <0.001 | 2.69 (1.78-4.76) | <0.001 |

Direct, indirect, and total effects and the proportion mediated were estimated using g-computation within the multistate framework, with transition-specific Cox models used for each disease-state transition. 95% CIs were derived from 2,000 bootstrap replications.

Models were adjusted for age, sex, race, smoking status, alcohol intake, physical activity, TDI, ALT, and AST.

CVD: cardiovascular disease ; ALT: alanine aminotransferase; AST: aspartate aminotransferase; TDI: Townsend deprivation index;

SII: immune-inflammation index

| **Table S24. Mediated role of IBI in associations of cardiometabolic burden with CVD and death transitions in multistate models** | | | | | |
| --- | --- | --- | --- | --- | --- |
| **Transitions** | **Effect Type** | **Effect Value** | **P-Value for effect** | **Mediated proportion (%)** | **P-Value for mediated proportion** |
| **Count-based Cardiometabolic Burden** |  |  |  |  |  |
| **Baseline to CVD** | Total | 1.1717 (1.1536-1.1908) | <0.001 |  |  |
|  | Direct | 1.1710 (1.1528-1.1900) | <0.001 |  |  |
|  | Indirect | 1.0006 (1.0003-1.0010) | 0.001 | 0.40 (0.18-0.61) | 0.001 |
| **Baseline to Death** | Total | 1.0966 (1.0652-1.1336) | <0.001 |  |  |
|  | Direct | 1.0956 (1.0646-1.1323) | <0.001 |  |  |
|  | Indirect | 1.0009 (1.0004-1.0016) | 0.008 | 0.98 (0.37-1.85) | 0.012 |
| **CVD to Death** | Total | 1.1228 (1.0894-1.1615) | <0.001 |  |  |
|  | Direct | 1.1215 (1.0878-1.1600) | <0.001 |  |  |
|  | Indirect | 1.0011 (0.9999-1.0024) | 0.085 | 0.98 (-0.07-2.36) | 0.116 |
| **Weighted Cardiometabolic Burden Score** |  |  |  |  |  |
| **Baseline to CVD** | Total | 1.2257 (1.2005-1.2500) | <0.001 |  |  |
|  | Direct | 1.2230 (1.1979-1.2469) | <0.001 |  |  |
|  | Indirect | 1.0022 (1.0017-1.0033) | <0.001 | 1.09 (0.79-1.65) | <0.001 |
| **Baseline to Death** | Total | 1.1199 (1.0853-1.1653) | <0.001 |  |  |
|  | Direct | 1.1164 (1.0824-1.1618) | <0.001 |  |  |
|  | Indirect | 1.0031 (1.0025-1.0051) | <0.001 | 2.76 (2.01-4.69) | <0.001 |
| **CVD to Death** | Total | 1.1665 (1.1195-1.2055) | <0.001 |  |  |
|  | Direct | 1.1628 (1.1161-1.2011) | <0.001 |  |  |
|  | Indirect | 1.0032 (1.0022-1.0052) | <0.001 | 2.07 (1.41-3.89) | <0.001 |

Direct, indirect, and total effects and the proportion mediated were estimated using g-computation within the multistate framework, with transition-specific Cox models used for each disease-state transition. 95% CIs were derived from 2,000 bootstrap replications.

Models were adjusted for age, sex, race, smoking status, alcohol intake, physical activity, TDI, ALT, and AST.

CVD: cardiovascular disease ; ALT: alanine aminotransferase; AST: aspartate aminotransferase; TDI: Townsend deprivation ;

IBI: inflammatory burden index

| **Table S25. Mediated role of NLR in associations of cardiometabolic burden with CVD and death transitions in multistate models** | | | | | |
| --- | --- | --- | --- | --- | --- |
| **Transitions** | **Effect Type** | **Effect Value** | **P-Value for effect** | **Mediated proportion (%)** | **P-Value for mediated proportion** |
| **Count-based Cardiometabolic Burden** |  |  |  |  |  |
| **Baseline to CVD** | Total | 1.1715 (1.1552-1.1900) | <0.001 |  |  |
|  | Direct | 1.1714 (1.1551-1.1899) | <0.001 |  |  |
|  | Indirect | 1.0000 (0.9998-1.0003) | 0.874 | 0.01 (-0.15-0.15) | 0.872 |
| **Baseline to Death** | Total | 1.0945 (1.0654-1.1251) | <0.001 |  |  |
|  | Direct | 1.0944 (1.0654-1.1248) | <0.001 |  |  |
|  | Indirect | 1.0000 (0.9996-1.0005) | 0.875 | 0.04 (-0.44-0.61) | 0.883 |
| **CVD to Death** | Total | 1.1218 (1.0947-1.1504) | <0.001 |  |  |
|  | Direct | 1.1211 (1.0943-1.1513) | <0.001 |  |  |
|  | Indirect | 1.0006 (0.9994-1.0019) | 0.332 | 0.54 (-0.51-1.74) | 0.337 |
| **Weighted Cardiometabolic Burden Score** |  |  |  |  |  |
| **Baseline to CVD** | Total | 1.2241 (1.2060-1.2464) | <0.001 |  |  |
|  | Direct | 1.2232 (1.2051-1.2455) | <0.001 |  |  |
|  | Indirect | 1.0007 (1.0003-1.0011) | 0.003 | 0.33 (0.14-0.57) | 0.003 |
| **Baseline to Death** | Total | 1.1199 (1.0858-1.1570) | <0.001 |  |  |
|  | Direct | 1.1185 (1.0844-1.1558) | <0.001 |  |  |
|  | Indirect | 1.0012 (1.0006-1.0020) | 0.002 | 1.03 (0.46-1.95) | 0.007 |
| **CVD to Death** | Total | 1.1659 (1.1280-1.2098) | <0.001 |  |  |
|  | Direct | 1.1639 (1.1269-1.2084) | <0.001 |  |  |
|  | Indirect | 1.0017 (1.0004-1.0032) | 0.023 | 1.13 (0.26-2.18) | 0.031 |

Direct, indirect, and total effects and the proportion mediated were estimated using g-computation within the multistate framework, with transition-specific Cox models used for each disease-state transition. 95% CIs were derived from 2,000 bootstrap replications.

Models were adjusted for age, sex, race, smoking status, alcohol intake, physical activity, TDI, ALT, and AST.

CVD: cardiovascular disease ; ALT: alanine aminotransferase; AST: aspartate aminotransferase; TDI: Townsend deprivation ;

NLR: neutrophil-to-lymphocyte ratio

| **Table S26. Mediated role of smoking status in associations of cardiometabolic burden with CVD and death transitions in multistate models** | | | | | |
| --- | --- | --- | --- | --- | --- |
| **Transitions** | **Effect Type** | **Effect Value** | **P-Value for effect** | **Mediated proportion (%)** | **P-Value for mediated proportion** |
| **Count-based Cardiometabolic Burden** |  |  |  |  |  |
| **Baseline to CVD** | Total | 1.1802 (1.1596-1.1999) | <0.001 |  |  |
|  | Direct | 1.1717 (1.1501-1.1915) | <0.001 |  |  |
|  | Indirect | 1.0073 (1.0056-1.0088) | <0.001 | 4.40 (3.38-5.41) | <0.001 |
| **Baseline to Death** | Total | 1.1080 (1.0703-1.1426) | <0.001 |  |  |
|  | Direct | 1.0914 (1.0538-1.1248) | <0.001 |  |  |
|  | Indirect | 1.0151 (1.0121-1.0188) | <0.001 | 14.66 (10.41-23.73) | <0.001 |
| **CVD to Death** | Total | 1.1437 (1.1159-1.1771) | <0.001 |  |  |
|  | Direct | 1.1267 (1.0977-1.1598) | <0.001 |  |  |
|  | Indirect | 1.0151 (1.0099-1.0187) | <0.001 | 11.15 (7.19-15.55) | <0.001 |
| **Weighted Cardiometabolic Burden Score** |  |  |  |  |  |
| **Baseline to CVD** | Total | 1.2296 (1.2063-1.2491) | <0.001 |  |  |
|  | Direct | 1.2253 (1.2028-1.2454) | <0.001 |  |  |
|  | Indirect | 1.0035 (1.0026-1.0049) | <0.001 | 1.68 (1.23-2.39) | <0.001 |
| **Baseline to Death** | Total | 1.1248 (1.0943-1.1663) | <0.001 |  |  |
|  | Direct | 1.1167 (1.0864-1.1577) | <0.001 |  |  |
|  | Indirect | 1.0073 (1.0050-1.0101) | <0.001 | 6.19 (3.81-9.99) | <0.001 |
| **CVD to Death** | Total | 1.1844 (1.1502-1.2293) | <0.001 |  |  |
|  | Direct | 1.1762 (1.1431-1.2201) | <0.001 |  |  |
|  | Indirect | 1.0070 (1.0023-1.0116) | 0.003 | 4.13 (1.25-7.20) | 0.003 |

Direct, indirect, and total effects and the proportion mediated were estimated using g-computation within the multistate framework, with transition-specific Cox models used for each disease-state transition. 95% CIs were derived from 2,000 bootstrap replications.

Models were adjusted for age, sex, race, smoking status, alcohol intake, physical activity, TDI, ALT, and AST.

CVD: cardiovascular disease ; ALT: alanine aminotransferase; AST: aspartate aminotransferase; TDI: Townsend deprivation index.

| **Table S27. Mediated role of drinking status in associations of cardiometabolic burden with CVD and death transitions in multistate models** | | | | | |
| --- | --- | --- | --- | --- | --- |
| **Transitions** | **Effect Type** | **Effect Value** | **P-Value for effect** | **Mediated proportion (%)** | **P-Value for mediated proportion** |
| **Count-based Cardiometabolic Burden** |  |  |  |  |  |
| **Baseline to CVD** | Total | 1.1807 (1.1576-1.1985) | <0.001 |  |  |
|  | Direct | 1.1744 (1.1522-1.1925) | <0.001 |  |  |
|  | Indirect | 1.0054 (1.0043-1.0069) | <0.001 | 3.22 (2.49-4.18) | <0.001 |
| **Baseline to Death** | Total | 1.1039 (1.0752-1.1302) | <0.001 |  |  |
|  | Direct | 1.0983 (1.0711-1.1249) | <0.001 |  |  |
|  | Indirect | 1.0051 (1.0028-1.0083) | <0.001 | 5.11 (2.78-8.19) | <0.001 |
| **CVD to Death** | Total | 1.1324 (1.0968-1.1754) | <0.001 |  |  |
|  | Direct | 1.1276 (1.0925-1.1697) | <0.001 |  |  |
|  | Indirect | 1.0042 (1.0019-1.0068) | 0.003 | 3.40 (1.51-5.89) | 0.006 |
| **Weighted Cardiometabolic Burden Score** |  |  |  |  |  |
| **Baseline to CVD** | Total | 1.2330 (1.2143-1.2503) | <0.001 |  |  |
|  | Direct | 1.2275 (1.2091-1.2440) | <0.001 |  |  |
|  | Indirect | 1.0045 (1.0034-1.0058) | <0.001 | 2.12 (1.60-2.82) | <0.001 |
| **Baseline to Death** | Total | 1.1290 (1.0943-1.1651) | <0.001 |  |  |
|  | Direct | 1.1249 (1.0904-1.1607) | <0.001 |  |  |
|  | Indirect | 1.0036 (1.0015-1.0060) | 0.003 | 2.99 (1.41-4.85) | 0.003 |
| **CVD to Death** | Total | 1.1834 (1.1420-1.2267) | <0.001 |  |  |
|  | Direct | 1.1787 (1.1364-1.2217) | <0.001 |  |  |
|  | Indirect | 1.0041 (1.0010-1.0075) | 0.023 | 2.40 (0.57-4.89) | 0.033 |

Direct, indirect, and total effects and the proportion mediated were estimated using g-computation within the multistate framework, with transition-specific Cox models used for each disease-state transition. 95% CIs were derived from 2,000 bootstrap replications.

Models were adjusted for age, sex, race, smoking status, alcohol intake, physical activity, TDI, ALT, and AST.

CVD: cardiovascular disease ; ALT: alanine aminotransferase; AST: aspartate aminotransferase; TDI: Townsend deprivation index

| **Table S28. Mediated role of physical activity in associations of cardiometabolic burden with CVD and death transitions in multistate models** | | | | | |
| --- | --- | --- | --- | --- | --- |
| **Transitions** | **Effect Type** | **Effect Value** | **P-Value for effect** | **Mediated proportion (%)** | **P-Value for mediated proportion** |
| **Count-based Cardiometabolic Burden** |  |  |  |  |  |
| **Baseline to CVD** | Total | 1.1745 (1.1514-1.2005) | <0.001 |  |  |
|  | Direct | 1.1720 (1.1488-1.1975) | <0.001 |  |  |
|  | Indirect | 1.0021 (1.0011-1.0032) | <0.001 | 1.33 (0.66-1.98) | <0.001 |
| **Baseline to Death** | Total | 1.0997 (1.0676-1.1419) | <0.001 |  |  |
|  | Direct | 1.0970 (1.0650-1.1375) | <0.001 |  |  |
|  | Indirect | 1.0025 (1.0007-1.0043) | 0.012 | 2.61 (0.83-5.30) | 0.020 |
| **CVD to Death** | Total | 1.1264 (1.0921-1.1650) | <0.001 |  |  |
|  | Direct | 1.1208 (1.0861-1.1590) | <0.001 |  |  |
|  | Indirect | 1.0050 (1.0026-1.0078) | <0.001 | 4.21 (1.75-7.52) | 0.005 |
| **Weighted Cardiometabolic Burden Score** |  |  |  |  |  |
| **Baseline to CVD** | Total | 1.2275 (1.2010-1.2549) | <0.001 |  |  |
|  | Direct | 1.2255 (1.1989-1.2526) | <0.001 |  |  |
|  | Indirect | 1.0016 (1.0008-1.0027) | 0.002 | 0.80 (0.37-1.35) | 0.002 |
| **Baseline to Death** | Total | 1.1186 (1.0802-1.1661) | <0.001 |  |  |
|  | Direct | 1.1165 (1.0785-1.1648) | <0.001 |  |  |
|  | Indirect | 1.0018 (1.0002-1.0035) | 0.043 | 1.65 (0.12-3.53) | 0.084 |
| **CVD to Death** | Total | 1.1771 (1.1233-1.2278) | <0.001 |  |  |
|  | Direct | 1.1717 (1.1193-1.2225) | <0.001 |  |  |
|  | Indirect | 1.0046 (1.0018-1.0083) | 0.006 | 2.82 (1.07-5.03) | 0.006 |

Direct, indirect, and total effects and the proportion mediated were estimated using g-computation within the multistate framework, with transition-specific Cox models used for each disease-state transition. 95% CIs were derived from 2,000 bootstrap replications.

Models were adjusted for age, sex, race, smoking status, alcohol intake, physical activity, TDI, ALT, and AST.

CVD: cardiovascular disease ; ALT: alanine aminotransferase; AST: aspartate aminotransferase; TDI: Townsend deprivation index

| **Table S29. Mediated role of diet in associations of cardiometabolic burden with CVD and death transitions in multistate models** | | | | | |
| --- | --- | --- | --- | --- | --- |
| **Transitions** | **Effect Type** | **Effect Value** | **P-Value for effect** | **Mediated proportion (%)** | **P-Value for mediated proportion** |
| **Count-based Cardiometabolic Burden** |  |  |  |  |  |
| **Baseline to CVD** | Total | 1.1712 (1.1541-1.1891) | <0.001 |  |  |
|  | Direct | 1.1713 (1.1542-1.1891) | <0.001 |  |  |
|  | Indirect | 1.0000 (0.9999-1.0001) | 0.679 | -0.01 (-0.09-0.05) | 0.679 |
| **Baseline to Death** | Total | 1.0923 (1.0601-1.1233) | <0.001 |  |  |
|  | Direct | 1.0923 (1.0600-1.1233) | <0.001 |  |  |
|  | Indirect | 1.0000 (0.9998-1.0002) | 0.900 | -0.01 (-0.22-0.20) | 0.900 |
| **CVD to Death** | Total | 1.1295 (1.1020-1.1712) | <0.001 |  |  |
|  | Direct | 1.1296 (1.1021-1.1711) | <0.001 |  |  |
|  | Indirect | 0.9999 (0.9990-1.0004) | 0.771 | -0.07 (-0.82-0.35) | 0.776 |
| **Weighted Cardiometabolic Burden Score** |  |  |  |  |  |
| **Baseline to CVD** | Total | 1.2241 (1.2038-1.2433) | <0.001 |  |  |
|  | Direct | 1.2241 (1.2038-1.2433) | <0.001 |  |  |
|  | Indirect | 1.0000 (0.9999-1.0001) | 0.896 | 0.00 (-0.05-0.06) | 0.894 |
| **Baseline to Death** | Total | 1.1151 (1.0813-1.1573) | <0.001 |  |  |
|  | Direct | 1.1151 (1.0813-1.1573) | <0.001 |  |  |
|  | Indirect | 1.0000 (0.9998-1.0002) | 0.993 | 0.00 (-0.16-0.17) | 0.993 |
| **CVD to Death** | Total | 1.1747 (1.1361-1.2184) | <0.001 |  |  |
|  | Direct | 1.1747 (1.1360-1.2185) | <0.001 |  |  |
|  | Indirect | 1.0000 (0.9995-1.0009) | 0.887 | 0.03 (-0.36-0.55) | 0.887 |

Direct, indirect, and total effects and the proportion mediated were estimated using g-computation within the multistate framework, with transition-specific Cox models used for each disease-state transition. 95% CIs were derived from 2,000 bootstrap replications.

Models were adjusted for age, sex, race, smoking status, alcohol intake, physical activity, TDI, ALT, and AST.

CVD: cardiovascular disease ; ALT: alanine aminotransferase; AST: aspartate aminotransferase; TDI: Townsend deprivation index

| **Table S30. Subgroup Analyses of the Associations Between Cardiometabolic Burden and Multistate Transitions by Age and Sex** | | | | | | | | | |
| --- | --- | --- | --- | --- | --- | --- | --- | --- | --- |
| **Exposure** | **Transition** | **Subgroup** | **Category** | **N** | **Events** | **Event rate (%** | **HR (95% CI)** | **P value** | **P for interaction** |
| **Count-based Cardiometabolic Burden**  **(Per 1 Higher Count)** | **Baseline to CVD** | **Age** | <60 | 40463 | 5809 | 14.4 | 1.26 (1.23–1.29) | <0.001 | <0.001 |
|  |  |  | >=60 | 32459 | 9543 | 29.4 | 1.17 (1.15–1.19) | <0.001 |  |
|  |  | **Sex** | Female | 29931 | 5438 | 18.2 | 1.28 (1.25–1.31) | <0.001 | 0.863 |
|  |  |  | Male | 42991 | 9914 | 23.1 | 1.30 (1.27–1.33) | <0.001 |  |
|  | **Baseline to Death** | **Age** | <60 | 40463 | 1389 | 3.4 | 1.19 (1.13–1.25) | <0.001 | 0.006 |
|  |  |  | >=60 | 32459 | 2884 | 8.9 | 1.10 (1.06–1.14) | <0.001 |  |
|  |  | **Sex** | Female | 29931 | 1695 | 5.7 | 1.21 (1.15–1.26) | <0.001 | 0.306 |
|  |  |  | Male | 42991 | 2578 | 6.0 | 1.27 (1.22–1.32) | <0.001 |  |
|  | **CVD to Death** | **Age** | <60 | 5808 | 1016 | 17.5 | 1.21 (1.13–1.28) | <0.001 | 0.101 |
|  |  |  | >=60 | 9542 | 3212 | 33.7 | 1.13 (1.09–1.17) | <0.001 |  |
|  |  | **Sex** | Female | 5437 | 1442 | 26.5 | 1.19 (1.13–1.25) | <0.001 | 0.613 |
|  |  |  | Male | 9913 | 2786 | 28.1 | 1.22 (1.17–1.26) | <0.001 |  |
| **Count-based Cardiometabolic Burden**  **(High vs. low)** | **Baseline to CVD** | **Age** | <60 | 40463 | 5809 | 14.4 | 1.52 (1.42–1.62) | <0.001 | <0.001 |
|  |  |  | >=60 | 32459 | 9543 | 29.4 | 1.25 (1.18–1.33) | <0.001 |  |
|  |  | **Sex** | Female | 29931 | 5438 | 18.2 | 1.54 (1.43–1.66) | <0.001 | 0.342 |
|  |  |  | Male | 42991 | 9914 | 23.1 | 1.50 (1.43–1.59) | <0.001 |  |
|  | **Baseline to Death** | **Age** | <60 | 40463 | 1389 | 3.4 | 1.27 (1.12–1.45) | <0.001 | 0.195 |
|  |  |  | >=60 | 32459 | 2884 | 8.9 | 1.16 (1.05–1.29) | 0.004 |  |
|  |  | **Sex** | Female | 29931 | 1695 | 5.7 | 1.36 (1.20–1.55) | <0.001 | 0.946 |
|  |  |  | Male | 42991 | 2578 | 6.0 | 1.41 (1.27–1.57) | <0.001 |  |
|  | **CVD to Death** | **Age** | <60 | 5808 | 1016 | 17.5 | 1.24 (1.04–1.47) | 0.015 | 0.447 |
|  |  |  | >=60 | 9544 | 3212 | 33.7 | 1.13 (1.01–1.25) | 0.025 |  |
|  |  | **Sex** | Female | 5437 | 1442 | 26.5 | 1.38 (1.17–1.63) | <0.001 | 0.077 |
|  |  |  | Male | 9915 | 2786 | 28.1 | 1.20 (1.08–1.34) | 0.001 |  |
| **Weighted Cardiometabolic Burden Score**  **(Per 1 SD)** | **Baseline to CVD** | **Age** | <60 | 40463 | 5809 | 14.4 | 1.29 (1.26–1.33) | <0.001 | 0.004 |
|  |  |  | >=60 | 32459 | 9543 | 29.4 | 1.23 (1.20–1.26) | <0.001 |  |
|  |  | **Sex** | Female | 29931 | 5438 | 18.2 | 1.34 (1.30–1.38) | <0.001 | 0.738 |
|  |  |  | Male | 42991 | 9914 | 23.1 | 1.37 (1.34–1.40) | <0.001 |  |
|  | **Baseline to Death** | **Age** | <60 | 40463 | 1389 | 3.4 | 1.21 (1.15–1.27) | <0.001 | 0.022 |
|  |  |  | >=60 | 32459 | 2884 | 8.9 | 1.13 (1.09–1.18) | <0.001 |  |
|  |  | **Sex** | Female | 29931 | 1695 | 5.7 | 1.22 (1.16–1.28) | <0.001 | 0.017 |
|  |  |  | Male | 42991 | 2578 | 6.0 | 1.33 (1.28–1.39) | <0.001 |  |
|  | **CVD to Death** | **Age** | <60 | 5808 | 1016 | 17.5 | 1.27 (1.18–1.35) | <0.001 | 0.124 |
|  |  |  | >=60 | 9544 | 3212 | 33.7 | 1.18 (1.14–1.23) | <0.001 |  |
|  |  | **Sex** | Female | 5437 | 1442 | 26.5 | 1.23 (1.16–1.31) | <0.001 | 0.253 |
|  |  |  | Male | 9915 | 2786 | 28.1 | 1.29 (1.24–1.35) | <0.001 |  |
| **Weighted Cardiometabolic Burden Score**  **(High vs. low)** | **Baseline to CVD** | **Age** | <60 | 40463 | 5809 | 14.4 | 1.43 (1.36–1.51) | <0.001 | 0.041 |
|  |  |  | >=60 | 32459 | 9543 | 29.4 | 1.34 (1.28–1.39) | <0.001 |  |
|  |  | **Sex** | Female | 29931 | 5438 | 18.2 | 1.53 (1.45–1.62) | <0.001 | 0.549 |
|  |  |  | Male | 42991 | 9914 | 23.1 | 1.54 (1.48–1.60) | <0.001 |  |
|  | **Baseline to Death** | **Age** | <60 | 40463 | 1389 | 3.4 | 1.34 (1.21–1.49) | <0.001 | 0.014 |
|  |  |  | >=60 | 32459 | 2884 | 8.9 | 1.16 (1.08–1.25) | <0.001 |  |
|  |  | **Sex** | Female | 29931 | 1695 | 5.7 | 1.31 (1.19–1.44) | <0.001 | 0.096 |
|  |  |  | Male | 42991 | 2578 | 6.0 | 1.47 (1.36–1.59) | <0.001 |  |
|  | **CVD to Death** | **Age** | <60 | 5808 | 1016 | 17.5 | 1.25 (1.10–1.42) | <0.001 | 0.746 |
|  |  |  | >=60 | 9544 | 3212 | 33.7 | 1.23 (1.15–1.32) | <0.001 |  |
|  |  | **Sex** | Female | 5437 | 1442 | 26.5 | 1.22 (1.10–1.36) | <0.001 | 0.076 |
|  |  |  | Male | 9915 | 2786 | 28.1 | 1.39 (1.29–1.50) | <0.001 |  |

HRs and 95% CIs were estimated using transition-specific Cox proportional hazards models. Count-based cardiometabolic burden was modelled either continuously per one additional metabolic abnormality or categorically as high versus low. The weighted cardiometabolic burden score was modelled per 1-SD increment or as high versus low.

Age-stratified models were adjusted for sex, race, alcohol intake, smoking, physical activity, ALT, AST, and Townsend deprivation index; sex-stratified models were adjusted for age and the remaining covariates.

P values for interaction were derived from likelihood-ratio tests comparing models with and without the exposure-by-subgroup interaction term. N and Events correspond to the exact complete-case risk set used for each stratum-specific model.

| **Table S31. Sensitivity Analysis I: Associations of cardiometabolic burden with CVD and Death Evaluated by Traditional Cox and Multistate Models after excluding self-reported outcome events.** | | | | | | |
| --- | --- | --- | --- | --- | --- | --- |
|  | **N** | **Events** | **Proportion(%)** | **HR(95%CI)** | **P-value** | **P for FDR** |
| **Traditional Cox model** |  |  |  |  |  |  |
| **Count-based Cardiometabolic Burden(Per 1 higher count)** |  |  |  |  |  |  |
| CVD | 72476 | 14906 | 20.57% | 1.18(1.16-1.20) | <0.001 |  |
| Death | 72476 | 8476 | 11.69% | 1.17(1.14-1.19) | <0.001 |  |
| **Count-based Cardiometabolic Burden(High vs. low)** |  |  |  |  |  |  |
| CVD | 56990 | 12609 | 22.12% | 1.30(1.24-1.36) | <0.001 |  |
| Death | 56990 | 7214 | 12.66% | 1.22(1.15-1.30) | <0.001 |  |
| **Weighted Cardiometabolic Burden Score(Per 1 higher SD)** |  |  |  |  |  |  |
| CVD | 72476 | 14906 | 20.57% | 1.23(1.21-1.25) | <0.001 |  |
| Death | 72476 | 8476 | 11.69% | 1.22(1.19-1.25) | <0.001 |  |
| **Weighted Cardiometabolic Burden Score(High vs. low)** |  |  |  |  |  |  |
| CVD | 31753 | 7941 | 25.01% | 1.35(1.30-1.39) | <0.001 |  |
| Death | 31753 | 4678 | 14.73% | 1.30(1.25-1.36) | <0.001 |  |
| **Multistate model** |  |  |  |  |  |  |
| **Count-based Cardiometabolic Burden(Per 1 higher count)** |  |  |  |  |  |  |
| Baseline to CVD | 72476 | 14906 | 20.57% | 1.18(1.16-1.20) | <0.001 | <0.001 |
| Baseline to Death | 72476 | 4273 | 5.90% | 1.09(1.06-1.13) | <0.001 | <0.001 |
| CVD to Death | 14906 | 4203 | 28.20% | 1.13(1.09-1.16) | <0.001 | <0.001 |
| **Count-based Cardiometabolic Burden(High vs. low)** |  |  |  |  |  |  |
| Baseline to CVD | 56989 | 12609 | 22.13% | 1.30(1.25-1.36) | <0.001 | <0.001 |
| Baseline to Death | 56989 | 3557 | 6.24% | 1.14(1.05-1.23) | 0.002 | 0.003 |
| CVD to Death | 12609 | 3657 | 29.00% | 1.13(1.03-1.24) | 0.008 | 0.008 |
| **Weighted Cardiometabolic Burden Score(Per 1 higher SD)** |  |  |  |  |  |  |
| Baseline to CVD | 72476 | 14906 | 20.57% | 1.23(1.21-1.25) | <0.001 | <0.001 |
| Baseline to Death | 72476 | 4273 | 5.90% | 1.12(1.08-1.16) | <0.001 | <0.001 |
| CVD to Death | 14906 | 4203 | 28.20% | 1.18(1.14-1.22) | <0.001 | <0.001 |
| **Weighted Cardiometabolic Burden Score(High vs. low)** |  |  |  |  |  |  |
| Baseline to CVD | 31753 | 7941 | 25.01% | 1.35(1.30-1.39) | <0.001 | <0.001 |
| Baseline to Death | 31753 | 2171 | 6.84% | 1.17(1.1-1.24) | <0.001 | <0.001 |
| CVD to Death | 7941 | 2507 | 31.57% | 1.20(1.13-1.28) | <0.001 | <0.001 |

The values are hazard ratios (HRs) and their 95% confidence intervals (CIs) from multivariable Cox proportional hazards models and Multistate models. Models were adjusted for age, sex, race, smoking status, alcohol intake, physical activity, TDI, ALT, and AST. Nominal P values are reported alongside FDR-adjusted P values, which were calculated using the Benjamini–Hochberg. For continuous exposure analyses, N represents the total analytic sample; for high-versus-low analyses, N and Events represent the number of participants and events in the high-burden group, respectively, with the low-burden group included as the reference category. Proportion (%) was calculated as Events/N within the corresponding group. CVD, cardiovascular disease; ALT, alanine aminotransferase; AST, aspartate aminotransferase; TDI, Townsend deprivation index

| **Table S32. Sensitivity AnalysisⅡ: Associations of cardiometabolic burden with CVD and Death Evaluated by Traditional Cox and Multistate Models after excluding participants who developed cardiovascular disease or died within the first year of follow-up.** | | | | | | |
| --- | --- | --- | --- | --- | --- | --- |
|  | **N** | **Events** | **Proportion(%)** | **HR(95%CI)** | **P-value** | **P for FDR** |
| **Traditional Cox model** |  |  |  |  |  |  |
| **Count-based Cardiometabolic Burden(Per 1 higher count)** |  |  |  |  |  |  |
| CVD | 72131 | 14615 | 20.26% | 1.17(1.15-1.19) | <0.001 |  |
| Death | 72131 | 8230 | 11.41% | 1.16(1.14-1.19) | <0.001 |  |
| **Count-based Cardiometabolic Burden(High vs. low)** |  |  |  |  |  |  |
| CVD | 56671 | 12336 | 21.77% | 1.29(1.24-1.35) | <0.001 |  |
| Death | 56671 | 6994 | 12.34% | 1.21(1.14-1.29) | <0.001 |  |
| **Weighted Cardiometabolic Burden Score(Per 1 higher SD)** |  |  |  |  |  |  |
| CVD | 72131 | 14615 | 20.26% | 1.22(1.20-1.25) | <0.001 |  |
| Death | 72131 | 8230 | 11.41% | 1.21(1.18-1.24) | <0.001 |  |
| **Weighted Cardiometabolic Burden Score(High vs. low)** |  |  |  |  |  |  |
| CVD | 31493 | 7717 | 24.50% | 1.33(1.29-1.38) | <0.001 |  |
| Death | 31493 | 4517 | 14.34% | 1.29(1.23-1.35) | <0.001 |  |
| Multistate model |  |  |  |  |  |  |
| **Count-based Cardiometabolic Burden(Per 1 higher count)** |  |  |  |  |  |  |
| Baseline to CVD | 72131 | 14615 | 20.26% | 1.17(1.15-1.19) | <0.001 | <0.001 |
| Baseline to Death | 72131 | 4219 | 5.85% | 1.09(1.06-1.13) | <0.001 | <0.001 |
| CVD to Death | 14615 | 4011 | 27.44% | 1.13(1.10-1.17) | <0.001 | <0.001 |
| **Count-based Cardiometabolic Burden(High vs. low)** |  |  |  |  |  |  |
| Baseline to CVD | 56671 | 12336 | 21.77% | 1.29(1.24-1.35) | <0.001 | <0.001 |
| Baseline to Death | 56671 | 3512 | 6.20% | 1.13(1.05-1.23) | 0.003 | 0.004 |
| CVD to Death | 12336 | 3482 | 28.23% | 1.13(1.03-1.24) | 0.012 | 0.012 |
| **Weighted Cardiometabolic Burden Score(Per 1 higher SD)** |  |  |  |  |  |  |
| Baseline to CVD | 72131 | 14615 | 20.26% | 1.22(1.20-1.25) | <0.001 | <0.001 |
| Baseline to Death | 72131 | 4219 | 5.85% | 1.11(1.08-1.15) | <0.001 | <0.001 |
| CVD to Death | 14615 | 4011 | 27.44% | 1.18(1.14-1.23) | <0.001 | <0.001 |
| **Weighted Cardiometabolic Burden Score(High vs. low)** |  |  |  |  |  |  |
| Baseline to CVD | 31493 | 7717 | 24.50% | 1.33(1.29-1.38) | <0.001 | <0.001 |
| Baseline to Death | 31493 | 2135 | 6.78% | 1.16(1.09-1.24) | <0.001 | <0.001 |
| CVD to Death | 7717 | 2382 | 30.87% | 1.20(1.13-1.28) | <0.001 | <0.001 |

The values are hazard ratios (HRs) and their 95% confidence intervals (CIs) from multivariable Cox proportional hazards models and Multistate models. Models were adjusted for age, sex, race, smoking status, alcohol intake, physical activity, TDI, ALT, and AST. Nominal P values are reported alongside FDR-adjusted P values, which were calculated using the Benjamini–Hochberg. For continuous exposure analyses, N represents the total analytic sample; for high-versus-low analyses, N and Events represent the number of participants and events in the high-burden group, respectively, with the low-burden group included as the reference category. Proportion (%) was calculated as Events/N within the corresponding group. CVD, cardiovascular disease; ALT, alanine aminotransferase; AST, aspartate aminotransferase; TDI, Townsend deprivation index

| **Table S33. Sensitivity Analysis Ⅲ: Hazard Ratios for cardiometabolic burden and Disease Transition Risks Across Varying Time Intervals Among Participants with Same-Day CVD and Death Diagnoses.** | | | | | | | | | | | | | | | |
| --- | --- | --- | --- | --- | --- | --- | --- | --- | --- | --- | --- | --- | --- | --- | --- |
|  | ***30*** ***days*** |  |  | ***180*** ***days*** |  |  | ***1*** ***year*** |  |  | ***2*** ***years*** |  |  | ***5*** ***years*** |  |  |
|  | **HR(95%CI)** | **P-value** | **P for FDR** | **HR(95%CI)** | **P-value** | **P for FDR** | **HR(95%CI)** | **P-value** | **P for FDR** | **HR(95%CI)** | **P-value** | **P for FDR** | **HR(95%CI)** | **P-value** | **P for FDR** |
| **Multistate model** | | | |  |  |  |  |  |  |  |  |  |  |  |  |
| **Count-based Cardiometabolic Burden(Per 1 higher count)** | | | | | | | | | | | | | | | |
| Baseline to CVD | 1.17  (1.15-1.19) | <0.001 | <0.001 | 1.17  (1.15-1.19) | <0.001 | <0.001 | 1.17  (1.15-1.19) | <0.001 | <0.001 | 1.17  (1.15-1.19) | <0.001 | <0.001 | 1.17  (1.15-1.19) | <0.001 | <0.001 |
| Baseline to Death | 1.09  (1.06-1.13) | <0.001 | <0.001 | 1.09  (1.06-1.13) | <0.001 | <0.001 | 1.09  (1.06-1.13) | <0.001 | <0.001 | 1.09  (1.06-1.13) | <0.001 | <0.001 | 1.09  (1.06-1.13) | <0.001 | <0.001 |
| CVD to Death | 1.13  (1.10-1.17) | <0.001 | <0.001 | 1.13  (1.10-1.17) | <0.001 | <0.001 | 1.13  (1.1-1.17) | <0.001 | <0.001 | 1.13  (1.10-1.17) | <0.001 | <0.001 | 1.12  (1.09-1.16) | <0.001 | <0.001 |
| **Count-based Cardiometabolic Burden(High vs. low)** | | | | | | | | | | | | | | | |
| Baseline to CVD | 1.30  (1.24-1.36) | <0.001 | <0.001 | 1.30  (1.24-1.36) | <0.001 | <0.001 | 1.30  (1.24-1.36) | <0.001 | <0.001 | 1.30  (1.24-1.36) | <0.001 | <0.001 | 1.30  (1.24-1.36) | <0.001 | <0.001 |
| Baseline to Death | 1.13  (1.05-1.23) | 0.002 | 0.003 | 1.13  (1.05-1.23) | 0.002 | 0.003 | 1.13  (1.05-1.23) | 0.002 | 0.003 | 1.13  (1.05-1.23) | 0.002 | 0.003 | 1.13  (1.05-1.23) | 0.002 | 0.003 |
| CVD to Death | 1.14  (1.04-1.25) | 0.005 | 0.005 | 1.14  (1.04-1.25) | 0.005 | 0.005 | 1.14  (1.04-1.24) | 0.006 | 0.006 | 1.13  (1.03-1.23) | 0.01 | 0.010 | 1.10  (1-1.199) | 0.049 | 0.049 |
| **Weighted Cardiometabolic Burden Score(Per 1 higher SD)** | | | | | | | | | | | | | | | |
| Baseline to CVD | 1.23  (1.20-1.25) | <0.001 | <0.001 | 1.23  (1.20-1.25) | <0.001 | <0.001 | 1.23  (1.20-1.25) | <0.001 | <0.001 | 1.23  (1.20-1.25) | <0.001 | <0.001 | 1.23  (1.20-1.25) | <0.001 | <0.001 |
| Baseline to Death | 1.12  (1.08-1.16) | <0.001 | <0.001 | 1.12  (1.08-1.16) | <0.001 | <0.001 | 1.12  (1.08-1.16) | <0.001 | <0.001 | 1.12  (1.08-1.16) | <0.001 | <0.001 | 1.12  (1.08-1.16) | <0.001 | <0.001 |
| CVD to Death | 1.19  (1.14-1.23) | <0.001 | <0.001 | 1.19  (1.15-1.23) | <0.001 | <0.001 | 1.19  (1.15-1.23) | <0.001 | <0.001 | 1.19  (1.15-1.23) | <0.001 | <0.001 | 1.18  (1.14-1.22) | <0.001 | <0.001 |
| **Weighted Cardiometabolic Burden Score(High vs. low)** | | | | | | | | | | | | | | | |
| Baseline to CVD | 1.34  (1.29-1.38) | <0.001 | <0.001 | 1.34  (1.29-1.38) | <0.001 | <0.001 | 1.34  (1.29-1.38) | <0.001 | <0.001 | 1.34  (1.30-1.38) | <0.001 | <0.001 | 1.34  (1.29-1.38) | <0.001 | <0.001 |
| Baseline to Death | 1.17  (1.1-1.24) | <0.001 | <0.001 | 1.17  (1.1-1.24) | <0.001 | <0.001 | 1.17  (1.1-1.24) | <0.001 | <0.001 | 1.17  (1.1-1.24) | <0.001 | <0.001 | 1.17  (1.1-1.24) | <0.001 | <0.001 |
| CVD to Death | 1.22  (1.14-1.30) | <0.001 | <0.001 | 1.22  (1.14-1.30) | <0.001 | <0.001 | 1.22  (1.14-1.30) | <0.001 | <0.001 | 1.22  (1.14-1.30) | <0.001 | <0.001 | 1.21  (1.13-1.29) | <0.001 | <0.001 |

The values are hazard ratios (HRs) and their 95% confidence intervals (CIs) from multivariable Cox proportional hazards models and Multistate models. Models were adjusted for age, sex, race, smoking status, alcohol intake, physical activity, TDI, ALT, and AST. Nominal P values are reported alongside FDR-adjusted P values, which were calculated using the Benjamini–Hochberg. For continuous exposure analyses, N represents the total analytic sample; for high-versus-low analyses, N and Events represent the number of participants and events in the high-burden group, respectively, with the low-burden group included as the reference category. Proportion (%) was calculated as Events/N within the corresponding group. CVD, cardiovascular disease; ALT, alanine aminotransferase; AST, aspartate aminotransferase; TDI, Townsend deprivation index

| **Table S34. Sensitivity AnalysisⅣ: Associations of cardiometabolic burden with CVD and Death Evaluated by Traditional Cox and Multistate Models After Inclusion of Participants with Baseline Cancer.** | | | | | | |
| --- | --- | --- | --- | --- | --- | --- |
|  | **N** | **Events** | **Proportion(%)** | **HR(95%CI)** | **P-value** | **P for FDR** |
| **Traditional Cox model** |  |  |  |  |  |  |
| **Count-based Cardiometabolic Burden(Per 1 higher count)** |  |  |  |  |  |  |
| CVD | 80391 | 17351 | 21.58% | 1.17(1.15-1.19) | <0.001 |  |
| Death | 80391 | 10348 | 12.87% | 1.15(1.13-1.17) | <0.001 |  |
| **Count-based Cardiometabolic Burden(High vs. low)** |  |  |  |  |  |  |
| CVD | 63483 | 14687 | 23.14% | 1.29(1.23-1.34) | <0.001 |  |
| Death | 63483 | 8808 | 13.87% | 1.20(1.14-1.27) | <0.001 |  |
| **Weighted Cardiometabolic Burden Score(Per 1 higher SD)** |  |  |  |  |  |  |
| CVD | 80391 | 17351 | 21.58% | 1.22(1.20-1.24) | <0.001 |  |
| Death | 80391 | 10348 | 12.87% | 1.20(1.17-1.23) | <0.001 |  |
| **Weighted Cardiometabolic Burden Score(High vs. low)** |  |  |  |  |  |  |
| CVD | 24595 | 6675 | 27.14% | 1.31(1.27-1.35) | <0.001 |  |
| Death | 24595 | 4390 | 17.85% | 1.39(1.34-1.45) | <0.001 |  |
| **Multistate model** |  |  |  |  |  |  |
| **Count-based Cardiometabolic Burden(Per 1 higher count)** |  |  |  |  |  |  |
| Baseline to CVD | 80391 | 17351 | 21.58% | 1.16(1.15-1.19) | <0.001 | <0.001 |
| Baseline to Death | 80391 | 5345 | 6.65% | 1.08(1.05-1.11) | <0.001 | <0.001 |
| CVD to Death | 17351 | 5003 | 28.83% | 1.12(1.09-1.16) | <0.001 | <0.001 |
| **Count-based Cardiometabolic Burden(High vs. low)** |  |  |  |  |  |  |
| Baseline to CVD | 63483 | 14687 | 23.14% | 1.29(1.24-1.34) | <0.001 | <0.001 |
| Baseline to Death | 63483 | 4448 | 7.01% | 1.12(1.04-1.20) | 0.003 | 0.003 |
| CVD to Death | 14687 | 4360 | 29.69% | 1.14(1.05-1.24) | 0.002 | 0.003 |
| **Weighted Cardiometabolic Burden Score(Per 1 higher SD)** |  |  |  |  |  |  |
| Baseline to CVD | 80391 | 17351 | 21.58% | 1.22(1.20-1.24) | <0.001 | <0.001 |
| Baseline to Death | 80391 | 5345 | 6.65% | 1.11(1.07-1.14) | <0.001 | <0.001 |
| CVD to Death | 17351 | 5003 | 28.83% | 1.17(1.13-1.21) | <0.001 | <0.001 |
| **Weighted Cardiometabolic Burden Score(High vs. low)** |  |  |  |  |  |  |
| Baseline to CVD | 24595 | 6675 | 27.14% | 1.31(1.27-1.35) | <0.001 | <0.001 |
| Baseline to Death | 24595 | 2046 | 8.32% | 1.24(1.17-1.31) | <0.001 | <0.001 |
| CVD to Death | 6675 | 2344 | 35.12% | 1.35(1.27-1.43) | <0.001 | <0.001 |

The values are hazard ratios (HRs) and their 95% confidence intervals (CIs) from multivariable Cox proportional hazards models and Multistate models. Models were adjusted for age, sex, race, smoking status, alcohol intake, physical activity, TDI, ALT, and AST. Nominal P values are reported alongside FDR-adjusted P values, which were calculated using the Benjamini–Hochberg. For continuous exposure analyses, N represents the total analytic sample; for high-versus-low analyses, N and Events represent the number of participants and events in the high-burden group, respectively, with the low-burden group included as the reference category. Proportion (%) was calculated as Events/N within the corresponding group. CVD, cardiovascular disease; ALT, alanine aminotransferase; AST, aspartate aminotransferase; TDI, Townsend deprivation index.

| **Table S35. Sensitivity Analysis Ⅴ: Associations of cardiometabolic burden with CVD and Death Evaluated by Traditional Cox and Multistate Models based on five multiple imputed datasets combined using Rubin’s rules.** | | | | | | |
| --- | --- | --- | --- | --- | --- | --- |
|  | **N** | **Events** | **Proportion(%)** | **HR(95%CI)** | **P-value** | **P for FDR** |
| **Traditional Cox model** |  |  |  |  |  |  |
| **Count-based Cardiometabolic Burden(Per 1 higher count)** |  |  |  |  |  |  |
| CVD | 72922 | 15352 | 21.05% | 1.17(1.15-1.19) | <0.001 |  |
| Death | 72922 | 8501 | 11.66% | 1.17(1.14-1.19) | <0.001 |  |
| **Count-based Cardiometabolic Burden(High vs. low)** |  |  |  |  |  |  |
| CVD | 57350 | 12970 | 22.62% | 1.30(1.22-1.36) | <0.001 |  |
| Death | 57350 | 7236 | 12.62% | 1.22(1.15-1.30) | <0.001 |  |
| **Weighted Cardiometabolic Burden Score(Per 1 higher SD)** |  |  |  |  |  |  |
| CVD | 72922 | 15352 | 21.05% | 1.23(1.20-1.25) | <0.001 |  |
| Death | 72922 | 8501 | 11.66% | 1.22(1.19-1.25) | <0.001 |  |
| **Weighted Cardiometabolic Burden Score(High vs. low)** |  |  |  |  |  |  |
| CVD | 31942 | 8130 | 25.45% | 1.34(1.30-1.38) | <0.001 |  |
| Death | 31942 | 4695 | 14.70% | 1.31(1.25-1.37) | <0.001 |  |
| **Multistate model** |  |  |  |  |  |  |
| **Count-based Cardiometabolic Burden(Per 1 higher count)** |  |  |  |  |  |  |
| Baseline to CVD | 72922 | 15352 | 21.05% | 1.17(1.15-1.19) | <0.001 | <0.001 |
| Baseline to Death | 72922 | 4273 | 5.86% | 1.09(1.06-1.13) | <0.001 | <0.001 |
| CVD to Death | 15352 | 4228 | 27.54% | 1.14(1.10-1.17) | <0.001 | <0.001 |
| **Count-based Cardiometabolic Burden(High vs. low)** |  |  |  |  |  |  |
| Baseline to CVD | 57350 | 12970 | 22.62% | 1.30(1.24-1.36) | <0.001 | <0.001 |
| Baseline to Death | 57350 | 3557 | 6.20% | 1.14(1.05-1.23) | 0.002 | 0.002 |
| CVD to Death | 12970 | 3679 | 28.37% | 1.15(1.05-1.26) | 0.003 | 0.003 |
| **Weighted Cardiometabolic Burden Score(Per 1 higher SD)** |  |  |  |  |  |  |
| Baseline to CVD | 72922 | 15352 | 21.05% | 1.23(1.21-1.25) | <0.001 | <0.001 |
| Baseline to Death | 72922 | 4273 | 5.86% | 1.12(1.08-1.16) | <0.001 | <0.001 |
| CVD to Death | 15352 | 4228 | 27.54% | 1.19(1.15-1.23) | <0.001 | <0.001 |
| **Weighted Cardiometabolic Burden Score(High vs. low)** |  |  |  |  |  |  |
| Baseline to CVD | 31942 | 8130 | 25.45% | 1.34(1.30-1.38) | <0.001 | <0.001 |
| Baseline to Death | 31942 | 2171 | 6.80% | 1.17(1.10-1.25) | <0.001 | <0.001 |
| CVD to Death | 8130 | 2524 | 31.05% | 1.22(1.15-1.30) | <0.001 | <0.001 |

The values are hazard ratios (HRs) and their 95% confidence intervals (CIs) from multivariable Cox proportional hazards models and Multistate models. Models were adjusted for age, sex, race, smoking status, alcohol intake, physical activity, TDI, ALT, and AST. Nominal P values are reported alongside FDR-adjusted P values, which were calculated using the Benjamini–Hochberg. For continuous exposure analyses, N represents the total analytic sample; for high-versus-low analyses, N and Events represent the number of participants and events in the high-burden group, respectively, with the low-burden group included as the reference category. Proportion (%) was calculated as Events/N within the corresponding group. CVD, cardiovascular disease; ALT, alanine aminotransferase; AST, aspartate aminotransferase; TDI, Townsend deprivation index.

| **Table S36. Sensitivity AnalysisⅥ : Associations of cardiometabolic burden with CVD and Death Evaluated by Traditional Cox and Multistate Models with additional adjustment for diet score, CRP, and eGFR.** | | | | | | |
| --- | --- | --- | --- | --- | --- | --- |
|  | **N** | **Events** | **Proportion(%)** | **HR(95%CI)** | **P-value** | **P for FDR** |
| **Traditional Cox model** |  |  |  |  |  |  |
| **Count-based Cardiometabolic Burden(Per 1 higher count)** |  |  |  |  |  |  |
| CVD | 72922 | 15352 | 21.05% | 1.17(1.15-1.19) | <0.001 |  |
| Death | 72922 | 8501 | 11.66% | 1.17(1.14-1.19) | <0.001 |  |
| **Count-based Cardiometabolic Burden(High vs. low)** |  |  |  |  | <0.001 |  |
| CVD | 57350 | 12970 | 22.62% | 1.29(1.24-1.35) | <0.001 |  |
| Death | 57350 | 7236 | 12.62% | 1.22(1.15-1.30) | <0.001 |  |
| **Weighted Cardiometabolic Burden Score(Per 1 higher SD)** |  |  |  |  | <0.001 |  |
| CVD | 72922 | 15352 | 21.05% | 1.22(1.20-1.24) | <0.001 |  |
| Death | 72922 | 8501 | 11.66% | 1.22(1.19-1.25) | <0.001 |  |
| **Weighted Cardiometabolic Burden Score(High vs. low)** |  |  |  |  | <0.001 |  |
| CVD | 31942 | 8130 | 25.45% | 1.33(1.29-1.37) | <0.001 |  |
| Death | 31942 | 4695 | 14.70% | 1.30(1.25-1.36) | <0.001 |  |
| **Multistate model** |  |  |  |  |  |  |
| **Count-based Cardiometabolic Burden(Per 1 higher count)** |  |  |  |  |  |  |
| Baseline to CVD | 72922 | 15352 | 21.05% | 1.17(1.15-1.19) | <0.001 | <0.001 |
| Baseline to Death | 72922 | 4273 | 5.86% | 1.09(1.06-1.13) | <0.001 | <0.001 |
| CVD to Death | 15352 | 4228 | 27.54% | 1.13(1.10-1.17) | <0.001 | <0.001 |
| **Count-based Cardiometabolic Burden(High vs. low)** |  |  |  |  |  |  |
| Baseline to CVD | 57350 | 12970 | 22.62% | 1.29(1.24-1.35) | <0.001 | <0.001 |
| Baseline to Death | 57350 | 3557 | 6.20% | 1.14(1.05-1.24) | 0.002 | 0.003 |
| CVD to Death | 12970 | 3679 | 28.37% | 1.14(1.04-1.25) | 0.004 | 0.004 |
| **Weighted Cardiometabolic Burden Score(Per 1 higher SD)** |  |  |  |  |  |  |
| Baseline to CVD | 72922 | 15352 | 21.05% | 1.22(1.20-1.24) | <0.001 | <0.001 |
| Baseline to Death | 72922 | 4273 | 5.86% | 1.12(1.08-1.15) | <0.001 | <0.001 |
| CVD to Death | 15352 | 4228 | 27.54% | 1.19(1.14-1.23) | <0.001 | <0.001 |
| **Weighted Cardiometabolic Burden Score(High vs. low)** |  |  |  |  |  |  |
| Baseline to CVD | 31942 | 8130 | 25.45% | 1.33(1.29-1.37) | <0.001 | <0.001 |
| Baseline to Death | 31942 | 2171 | 6.80% | 1.17(1.10-1.24) | <0.001 | <0.001 |
| CVD to Death | 8130 | 2524 | 31.05% | 1.22(1.14-1.30) | <0.001 | <0.001 |

The values are hazard ratios (HRs) and their 95% confidence intervals (CIs) from multivariable Cox proportional hazards models and Multistate models. Models were adjusted for age, sex, race, smoking status, alcohol intake, physical activity, TDI, ALT, and AST. Nominal P values are reported alongside FDR-adjusted P values, which were calculated using the Benjamini–Hochberg. For continuous exposure analyses, N represents the total analytic sample; for high-versus-low analyses, N and Events represent the number of participants and events in the high-burden group, respectively, with the low-burden group included as the reference category. Proportion (%) was calculated as Events/N within the corresponding group. CVD, cardiovascular disease; ALT, alanine aminotransferase; AST, aspartate aminotransferase; TDI, Townsend deprivation index.

| **Table S37. Sensitivity AnalysisⅦ : Associations of cardiometabolic burden with CVD and Death Evaluated by Traditional Cox and Multistate Models after redefining cardiovascular disease as a composite of coronary artery disease, stroke, and heart failure.** | | | | | | |
| --- | --- | --- | --- | --- | --- | --- |
|  | **N** | **Events** | **Proportion(%)** | **HR(95%CI)** | **P-value** | **P for FDR** |
| **Traditional Cox model** |  |  |  |  |  |  |
| **Count-based Cardiometabolic Burden(Per 1 higher count)** |  |  |  |  |  |  |
| CVD | 72922 | 12442 | 17.06% | 1.20(1.18-1.22) | <0.001 |  |
| Death | 72922 | 8501 | 11.66% | 1.17(1.14-1.19) | <0.001 |  |
| **Count-based Cardiometabolic Burden(High vs. low)** |  |  |  |  |  |  |
| CVD | 57350 | 10656 | 18.58% | 1.41(1.34-1.49) | <0.001 |  |
| Death | 57350 | 7236 | 12.62% | 1.22(1.15-1.30) | <0.001 |  |
| **Weighted Cardiometabolic Burden Score(Per 1 higher SD)** |  |  |  |  |  |  |
| CVD | 72922 | 12442 | 17.06% | 1.23(1.20-1.26) | <0.001 |  |
| Death | 72922 | 8501 | 11.66% | 1.22(1.19-1.25) | <0.001 |  |
| **Weighted Cardiometabolic Burden Score(High vs. low)** |  |  |  |  |  |  |
| CVD | 34603 | 7337 | 21.20% | 1.35(1.3-1.41) | <0.001 |  |
| Death | 34603 | 5184 | 14.98% | 1.34(1.28-1.40) | <0.001 |  |
| **Multistate model** |  |  |  |  |  |  |
| **Count-based Cardiometabolic Burden(Per 1 higher count)** |  |  |  |  |  |  |
| Baseline to CVD | 72922 | 12442 | 17.06% | 1.22(1.19-1.24) | <0.001 | <0.001 |
| Baseline to Death | 72922 | 4849 | 6.65% | 1.1(1.07-1.13) | <0.001 | <0.001 |
| CVD to Death | 12442 | 3652 | 29.35% | 1.11(1.08-1.15) | <0.001 | <0.001 |
| **Count-based Cardiometabolic Burden(High vs. low)** |  |  |  |  |  |  |
| Baseline to CVD | 57350 | 10656 | 18.58% | 1.42(1.35-1.49) | <0.001 | <0.001 |
| Baseline to Death | 57350 | 4038 | 7.04% | 1.13(1.05-1.22) | 0.002 | 0.003 |
| CVD to Death | 10656 | 3198 | 30.01% | 1.09(0.99-1.21) | 0.075 | 0.075 |
| **Weighted Cardiometabolic Burden Score(Per 1 higher SD)** |  |  |  |  |  |  |
| Baseline to CVD | 72922 | 12442 | 17.06% | 1.26(1.23-1.28) | <0.001 | <0.001 |
| Baseline to Death | 72922 | 4849 | 6.65% | 1.13(1.1-1.17) | <0.001 | <0.001 |
| CVD to Death | 12442 | 3652 | 29.35% | 1.17(1.12-1.21) | <0.001 | <0.001 |
| **Weighted Cardiometabolic Burden Score(High vs. low)** |  |  |  |  |  |  |
| Baseline to CVD | 34603 | 7337 | 21.20% | 1.39(1.34-1.44) | <0.001 | <0.001 |
| Baseline to Death | 34603 | 2765 | 7.99% | 1.21(1.14-1.28) | <0.001 | <0.001 |
| CVD to Death | 7337 | 2419 | 32.97% | 1.23(1.15-1.32) | <0.001 | <0.001 |

The values are hazard ratios (HRs) and their 95% confidence intervals (CIs) from multivariable Cox proportional hazards models and Multistate models. Models were adjusted for age, sex, race, smoking status, alcohol intake, physical activity, TDI, ALT, and AST. Nominal P values are reported alongside FDR-adjusted P values, which were calculated using the Benjamini–Hochberg. For continuous exposure analyses, N represents the total analytic sample; for high-versus-low analyses, N and Events represent the number of participants and events in the high-burden group, respectively, with the low-burden group included as the reference category. Proportion (%) was calculated as Events/N within the corresponding group. CVD, cardiovascular disease; ALT, alanine aminotransferase; AST, aspartate aminotransferase; TDI, Townsend deprivation index.

| **Table S38. Sensitivity AnalysisⅧ : Associations of cardiometabolic burden with CVD and Death Evaluated by Traditional Cox and Multistate Models** | | | | | | |
| --- | --- | --- | --- | --- | --- | --- |
|  | **N** | **Events** | **Proportion(%)** | **HR(95%CI)** | **P-value** | **P for FDR** |
| **Training** |  |  |  |  |  |  |
| **Traditional Cox model** |  |  |  |  |  |  |
| **Count-based Cardiometabolic Burden(Per 1 higher count)** |  |  |  |  |  |  |
| CVD | 43753 | 9211 | 21.05% | 1.18(1.15–1.20) | <0.001 |  |
| Death | 43753 | 5117 | 11.70% | 1.16(1.13–1.19) | <0.001 |  |
| **Count-based Cardiometabolic Burden(High vs. low)** |  |  |  |  |  |  |
| CVD | 34352 | 7808 | 22.73% | 1.33(1.26–1.41) | <0.001 |  |
| Death | 34352 | 4353 | 12.67% | 1.23(1.14–1.33) | <0.001 |  |
| **Weighted Cardiometabolic Burden Score(Per 1 higher SD)** |  |  |  |  |  |  |
| CVD | 43753 | 9211 | 21.05% | 1.23(1.20–1.26) | <0.001 |  |
| Death | 43753 | 5117 | 11.70% | 1.22(1.18–1.26) | <0.001 |  |
| **Weighted Cardiometabolic Burden Score(High vs. low)** |  |  |  |  |  |  |
| CVD | 13297 | 3578 | 26.91% | 1.34(1.29–1.40) | <0.001 |  |
| Death | 13297 | 2207 | 16.60% | 1.44(1.36–1.53) | <0.001 |  |
| **Multistate model** |  |  |  |  |  |  |
| **Count-based Cardiometabolic Burden(Per 1 higher count)** |  |  |  |  |  |  |
| Baseline to CVD | 43753 | 9211 | 21.05% | 1.18(1.15–1.20) | <0.001 | <0.001 |
| Baseline to Death | 43753 | 2554 | 5.84% | 1.09(1.04–1.13) | <0.001 | <0.001 |
| CVD to Death | 9211 | 2563 | 27.83% | 1.13(1.09–1.18) | <0.001 | <0.001 |
| **Count-based Cardiometabolic Burden(High vs. low)** |  |  |  |  |  |  |
| Baseline to CVD | 34352 | 7808 | 22.73% | 1.34(1.26–1.42) | <0.001 | <0.001 |
| Baseline to Death | 34352 | 2111 | 6.15% | 1.11(1.01–1.24) | 0.002 | 0.040 |
| CVD to Death | 7808 | 2242 | 28.72% | 1.20(1.06–1.35) | 0.003 | 0.003 |
| **Weighted Cardiometabolic Burden Score(Per 1 higher SD)** |  |  |  |  |  |  |
| Baseline to CVD | 43753 | 9211 | 21.05% | 1.24(1.21–1.26) | <0.001 | <0.001 |
| Baseline to Death | 43753 | 2554 | 5.84% | 1.11(1.07–1.16) | <0.001 | <0.001 |
| CVD to Death | 9211 | 2563 | 27.83% | 1.19(1.14–1.25) | <0.001 | <0.001 |
| **Weighted Cardiometabolic Burden Score(High vs. low)** |  |  |  |  |  |  |
| Baseline to CVD | 13297 | 3578 | 26.91% | 1.35(1.29–1.41) | <0.001 | <0.001 |
| Baseline to Death | 13297 | 986 | 7.42% | 1.29(1.19–1.40) | <0.001 | <0.001 |
| CVD to Death | 3578 | 1221 | 34.13% | 1.37(1.27–1.49) | <0.001 | <0.001 |
| **Validation** |  |  |  |  |  |  |
| **Traditional Cox model** |  |  |  |  |  |  |
| **Count-based Cardiometabolic Burden(Per 1 higher count)** |  |  |  |  |  |  |
| CVD | 29169 | 6141 | 21.05% | 1.17(1.14–1.20) | <0.001 |  |
| Death | 29169 | 3384 | 11.60% | 1.17(1.13–1.21) | <0.001 |  |
| **Count-based Cardiometabolic Burden(High vs. low)** |  |  |  |  |  |  |
| CVD | 22998 | 5162 | 22.45% | 1.24(1.16–1.33) | <0.001 |  |
| Death | 22998 | 2883 | 12.54% | 1.20(1.09–1.32) | <0.001 |  |
| **Weighted Cardiometabolic Burden Score(Per 1 higher SD)** |  |  |  |  |  |  |
| CVD | 29169 | 6141 | 21.05% | 1.21(1.17–1.24) | <0.001 |  |
| Death | 29169 | 3384 | 11.60% | 1.23(1.18–1.27) | <0.001 |  |
| **Weighted Cardiometabolic Burden Score(High vs. low)** |  |  |  |  |  |  |
| CVD | 8696 | 2279 | 26.21% | 1.27(1.20–1.34) | <0.001 |  |
| Death | 8696 | 1424 | 16.38% | 1.39(1.30–1.50) | <0.001 |  |
| **Multistate model** |  |  |  |  |  |  |
| **Count-based Cardiometabolic Burden(Per 1 higher count)** |  |  |  |  |  |  |
| Baseline to CVD | 29169 | 6141 | 21.05% | 1.17(1.14–1.19) | <0.001 | <0.001 |
| Baseline to Death | 29169 | 1719 | 5.89% | 1.10(1.05–1.16) | <0.001 | <0.001 |
| CVD to Death | 6141 | 1665 | 27.12% | 1.14(1.08–1.20) | <0.001 | <0.001 |
| **Count-based Cardiometabolic Burden(High vs. low)** |  |  |  |  |  |  |
| Baseline to CVD | 22998 | 5162 | 22.45% | 1.24(1.16–1.33) | <0.001 | <0.001 |
| Baseline to Death | 22998 | 1446 | 6.29% | 1.17(1.03–1.33) | 0.002 | 0.019 |
| CVD to Death | 5162 | 1437 | 27.84% | 1.09(0.94–1.25) | 0.075 | 0.261 |
| **Weighted Cardiometabolic Burden Score(Per 1 higher SD)** |  |  |  |  |  |  |
| Baseline to CVD | 29169 | 6141 | 21.05% | 1.21(1.18–1.24) | <0.001 | <0.001 |
| Baseline to Death | 29169 | 1719 | 5.89% | 1.13(1.07–1.19) | <0.001 | <0.001 |
| CVD to Death | 6141 | 1665 | 27.12% | 1.20(1.14–1.27) | <0.001 | <0.001 |
| **Weighted Cardiometabolic Burden Score(High vs. low)** |  |  |  |  |  |  |
| Baseline to CVD | 8696 | 2279 | 26.21% | 1.27(1.21–1.34) | <0.001 | <0.001 |
| Baseline to Death | 8696 | 654 | 7.52% | 1.25(1.13–1.38) | <0.001 | <0.001 |
| CVD to Death | 2279 | 770 | 33.79% | 1.39(1.26–1.53) | <0.001 | <0.001 |

The values are hazard ratios (HRs) and their 95% confidence intervals (CIs) from multivariable Cox proportional hazards models and Multistate models. Models were adjusted for age, sex, race, smoking status, alcohol intake, physical activity, TDI, ALT, and AST. Nominal P values are reported alongside FDR-adjusted P values, which were calculated using the Benjamini–Hochberg. For continuous exposure analyses, N represents the total analytic sample; for high-versus-low analyses, N and Events represent the number of participants and events in the high-burden group, respectively, with the low-burden group included as the reference category. Proportion (%) was calculated as Events/N within the corresponding group. CVD, cardiovascular disease; ALT, alanine aminotransferase; AST, aspartate aminotransferase; TDI, Townsend deprivation index.

| **Table S39. Sensitivity Analysis Ⅸ A: Associations of Cardiometabolic Burden With Cardiovascular Outcomes Using Fine-Gray Competing Risk Models** | | | | | |
| --- | --- | --- | --- | --- | --- |
|  | **N** | **Events** | **Proportion(%)** | **SHR(95%CI)** | **P-value** |
| **Count-based Cardiometabolic Burden(Per 1 higher count)** |  |  |  |  |  |
| CVD | 72922 | 15352 | 21.05% | 1.17 (1.15–1.19) | <0.001 |
| CAD | 72922 | 9174 | 12.58% | 1.23 (1.21–1.26) | <0.001 |
| HF | 72922 | 3475 | 4.77% | 1.23 (1.19–1.28) | <0.001 |
| AF | 72922 | 6253 | 8.57% | 1.09 (1.06–1.11) | <0.001 |
| Stroke | 72922 | 2422 | 3.32% | 1.15 (1.10–1.20) | <0.001 |
| **Count-based Cardiometabolic Burden(High vs. low)** |  |  |  |  |  |
| CVD | 57350 | 12970 | 22.62% | 1.29(1.23–1.35) | <0.001 |
| CAD | 57350 | 7891 | 13.76% | 1.46 (1.38–1.55) | <0.001 |
| HF | 57350 | 2983 | 5.20% | 1.29 (1.17–1.42) | <0.001 |
| AF | 57350 | 5159 | 9.00% | 1.06 (0.99–1.13) | 0.114 |
| Stroke | 57350 | 2080 | 3.63% | 1.36 (1.21–1.52) | <0.001 |
| **Weighted Cardiometabolic Burden Score(Per 1 higher SD)** |  |  |  |  |  |
| CVD | 72922 | 15352 | 21.05% | 1.22(1.20–1.24) | <0.001 |
| CAD | 72922 | 9174 | 12.58% | 1.26 (1.23–1.29) | <0.001 |
| HF | 72922 | 3475 | 4.77% | 1.34 (1.29–1.39) | <0.001 |
| AF | 72922 | 6253 | 8.57% | 1.17 (1.14–1.20) | <0.001 |
| Stroke | 72922 | 2422 | 3.32% | 1.20 (1.15–1.25) | <0.001 |
| **Weighted Cardiometabolic Burden Score(High vs. low)** |  |  |  |  |  |
| CVD | 31942 | 8130 | 25.45% | 1.33 (1.28–1.37) | <0.001 |
| CAD | 31942 | 4968 | 15.55% | 1.39 (1.34–1.45) | <0.001 |
| HF | 31942 | 2029 | 6.35% | 1.45 (1.35–1.55) | <0.001 |
| AF | 31942 | 3347 | 10.48% | 1.28 (1.22–1.35) | <0.001 |
| Stroke | 31942 | 1285 | 4.02% | 1.22 (1.12–1.32) | <0.001 |

The values are hazard ratios (HRs) and their 95% confidence intervals (CIs) from multivariable Cox proportional hazards models and Multistate models. Models were adjusted for age, sex, race, smoking status, alcohol intake, physical activity, TDI, ALT, and AST. Nominal P values are reported alongside FDR-adjusted P values, which were calculated using the Benjamini–Hochberg. For continuous exposure analyses, N represents the total analytic sample; for high-versus-low analyses, N and Events represent the number of participants and events in the high-burden group, respectively, with the low-burden group included as the reference category. Proportion (%) was calculated as Events/N within the corresponding group. CVD, cardiovascular disease; ALT, alanine aminotransferase; AST, aspartate aminotransferase; TDI, Townsend deprivation index.

| **Table S40. Sensitivity Analysis X: Associations of Cardiometabolic Burden With CVD and Death After Redefining MASLD Using MRI-Derived PDFF, Evaluated by Traditional Cox and Multistate Models** | | | | | | |
| --- | --- | --- | --- | --- | --- | --- |
|  | **N** | **Events** | **Proportion(%)** | **HR(95%CI)** | **P-value** | **P for FDR** |
| **Traditional Cox model** |  |  |  |  |  |  |
| **Count-based Cardiometabolic Burden(Per 1 higher count)** |  |  |  |  |  |  |
| CVD | 4243 | 528 | 12.44% | 1.20(1.10-1.31) | <0.001 |  |
| Death | 4243 | 146 | 3.40% | 1.23(1.04-1.46) | <0.001 |  |
| **Count-based Cardiometabolic Burden(High vs. low)** |  |  |  |  |  |  |
| CVD | 2715 | 410 | 15.10% | 1.59(1.26-1.99) | <0.001 |  |
| Death | 2715 | 110 | 4.01% | 1.41(0.91-2.17) | 0.125 |  |
| **Weighted Cardiometabolic Burden Score(Per 1 higher SD)** |  |  |  |  |  |  |
| CVD | 4243 | 528 | 12.44% | 1.33(1.20-1.48) | <0.001 |  |
| Death | 4243 | 146 | 3.44% | 1.25(1.03-1.52) | <0.001 |  |
| **Weighted Cardiometabolic Burden Score(High vs. low)** |  |  |  |  |  |  |
| CVD | 2101 | 341 | 16.20% | 1.58(1.29-1.93) | <0.001 |  |
| Death | 2101 | 86 | 4.10% | 1.34(0.92-1.95) | 0.129 |  |
| **Multistate model** |  |  |  |  |  |  |
| **Count-based Cardiometabolic Burden(Per 1 higher count)** |  |  |  |  |  |  |
| Baseline to CVD | 4243 | 528 | 12.44% | 1.20(1.10-1.31) | <0.001 | <0.001 |
| Baseline to Death | 4243 | 81 | 1.91% | 1.17(0.94-1.46) | 0.156 | 0.156 |
| CVD to Death | 528 | 65 | 12.31% | 1.23(0.93-1.62) | 0.153 | 0.156 |
| **Count-based Cardiometabolic Burden(High vs. low)** |  |  |  |  |  |  |
| Baseline to CVD | 2715 | 410 | 15.10% | 1.58(1.26-2.00) | <0.001 | <0.001 |
| Baseline to Death | 2715 | 58 | 2.14% | 1.25(0.72-2.15) | 0.428 | 0.428 |
| CVD to Death | 410 | 52 | 12.68% | 1.37(0.65-2.89) | 0.414 | 0.428 |
| **Weighted Cardiometabolic Burden Score(Per 1 higher SD)** |  |  |  |  |  |  |
| Baseline to CVD | 4243 | 528 | 12.44% | 1.33(1.20-1.48) | <0.001 | <0.001 |
| Baseline to Death | 4243 | 81 | 1.91% | 1.21(0.94-1.56) | 0.143 | 0.214 |
| CVD to Death | 528 | 65 | 12.31% | 1.11(0.82-1.52) | 0.495 | 0.495 |
| **Weighted Cardiometabolic Burden Score(High vs. low)** |  |  |  |  |  |  |
| Baseline to CVD | 2101 | 341 | 16.23% | 1.58(1.29-1.93) | <0.001 | <0.001 |
| Baseline to Death | 2101 | 45 | 2.14% | 1.34(0.82-2.19) | 0.244 | 0.367 |
| CVD to Death | 341 | 41 | 12.02% | 1.02(0.56-1.86) | 0.941 | 0.941 |

The values are hazard ratios (HRs) and their 95% confidence intervals (CIs) from multivariable Cox proportional hazards models and Multistate models. Models were adjusted for age, sex, race, smoking status, alcohol intake, physical activity, TDI, ALT, and AST. Nominal P values are reported alongside FDR-adjusted P values, which were calculated using the Benjamini–Hochberg. For continuous exposure analyses, N represents the total analytic sample; for high-versus-low analyses, N and Events represent the number of participants and events in the high-burden group, respectively, with the low-burden group included as the reference category. Proportion (%) was calculated as Events/N within the corresponding group. CVD, cardiovascular disease; ALT, alanine aminotransferase; AST, aspartate aminotransferase; TDI, Townsend deprivation index.

| **Table S41. Sensitivity Analysis XⅠ: Tests for non-linearity of the associations between weighted metabolic score and multistate transitions** | | | | | | |
| --- | --- | --- | --- | --- | --- | --- |
| **Transition** | **Participants** | **Events** | **P for non linearity (df=2)** | **P for non linearity (df=4)** | **P for non linearity (df=5)** | **P for non linearity (df=6)** |
| Baseline to CVD | 72922 | 15352 | 0.022 | 0.036 | 0.044 | 0.055 |
| Baseline to Death | 72922 | 4273 | 0.007 | 0.002 | 0.001 | 0.002 |
| CVD to Death | 15352 | 4228 | 0.007 | 0.017 | 0.008 | 0.003 |

P values were derived from likelihood-ratio tests comparing the linear Cox model with spline models using 2, 4, 5, and 6 degrees of freedom.

Models were adjusted for age, sex, race, smoking status, alcohol intake, physical activity , TDI, ALT, and AST. P values for non-linearity are presented in each panel.

CVD: cardiovascular disease.

| **Table S42. Sensitivity Analysis XⅡ: Associations of Cardiometabolic Burden With CVD and Death Evaluated by Traditional Cox and Multistate Models With Log-Transformed Alcohol Intake, ALT, and AST** | | | | | | |
| --- | --- | --- | --- | --- | --- | --- |
|  | **N** | **Events** | **Proportion (%)** | **HR(95%CI)** | **P-value** | **FDR** |
| **Traditional Cox model** |  |  |  |  |  |  |
| **Count-based Cardiometabolic Burden(Per 1 higher count)** |  |  |  |  |  |  |
| CVD | 72922 | 15352 | 21.05% | 1.17(1.15-1.19) | <0.001 |  |
| Death | 72922 | 8501 | 11.66% | 1.17(1.14-1.19) | <0.001 |  |
| **Count-based Cardiometabolic Burden(High vs. low)** |  |  |  |  |  |  |
| CVD | 57350 | 12970 | 22.62% | 1.30(1.24-1.36) | <0.001 |  |
| Death | 57350 | 7236 | 12.62% | 1.23(1.15-1.30) | <0.001 |  |
| **Weighted Cardiometabolic Burden Score (Per 1 higher SD)** |  |  |  |  |  |  |
| CVD | 72922 | 15352 | 21.05% | 1.22(1.20-1.25) | <0.001 |  |
| Death | 72922 | 8501 | 11.66% | 1.22(1.19-1.25) | <0.001 |  |
| **Weighted Cardiometabolic Burden Score (High vs. low)** |  |  |  |  |  |  |
| CVD | 31942 | 8130 | 25.45% | 1.34(1.29-1.38) | <0.001 |  |
| Death | 31942 | 4695 | 14.70% | 1.31(1.25-1.36) | <0.001 |  |
| **Multistate model** |  |  |  |  |  |  |
| **Count-based Cardiometabolic Burden(Per 1 higher count)** |  |  |  |  |  |  |
| Baseline to CVD | 72922 | 15352 | 21.05% | 1.17(1.15-1.19) | <0.001 | <0.001 |
| Baseline to Death | 72922 | 4273 | 5.86% | 1.09(1.06-1.13) | <0.001 | <0.001 |
| CVD to Death | 15352 | 4228 | 27.54% | 1.13(1.10-1.17) | <0.001 | <0.001 |
| **Count-based Cardiometabolic Burden(High vs. low)** |  |  |  |  |  |  |
| Baseline to CVD | 57350 | 12970 | 22.62% | 1.30(1.24-1.36) | <0.001 | <0.001 |
| Baseline to Death | 57350 | 3557 | 6.20% | 1.14(1.05-1.23) | 0.002 | 0.003 |
| CVD to Death | 12970 | 3679 | 28.37% | 1.15(1.05-1.26) | 0.003 | 0.003 |
| **Weighted Cardiometabolic Burden Score (Per 1 higher SD)** |  |  |  |  |  |  |
| Baseline to CVD | 72922 | 15352 | 21.05% | 1.22(1.20-1.25) | <0.001 | <0.001 |
| Baseline to Death | 72922 | 4273 | 5.86% | 1.12(1.08-1.16) | <0.001 | <0.001 |
| CVD to Death | 15352 | 4228 | 27.54% | 1.19(1.15-1.23) | <0.001 | <0.001 |
| **Weighted Cardiometabolic Burden Score (High vs. low)** |  |  |  |  |  |  |
| Baseline to CVD | 31942 | 8130 | 25.45% | 1.34(1.29-1.38) | <0.001 | <0.001 |
| Baseline to Death | 31942 | 2171 | 6.80% | 1.17(1.10-1.24) | <0.001 | <0.001 |
| CVD to Death | 8130 | 2524 | 31.05% | 1.22(1.15-1.30) | <0.001 | <0.001 |

The values are hazard ratios (HRs) and their 95% confidence intervals (CIs) from multivariable Cox proportional hazards models and Multistate models. Models were adjusted for age, sex, race, smoking status, alcohol intake, physical activity, TDI, ALT, and AST. Nominal P values are reported alongside FDR-adjusted P values, which were calculated using the Benjamini–Hochberg. For continuous exposure analyses, N represents the total analytic sample; for high-versus-low analyses, N and Events represent the number of participants and events in the high-burden group, respectively, with the low-burden group included as the reference category. Proportion (%) was calculated as Events/N within the corresponding group. CVD, cardiovascular disease; ALT, alanine aminotransferase; AST, aspartate aminotransferase; TDI, Townsend deprivation index.

**Figure S1. The study flow chart**.

**Figure S2. Hazard ratios of five cardiometabolic abnormalities for CVD**

The values are hazard ratios (HRs) and their 95% confidence intervals (CIs) from multivariable Cox proportional hazards models for CVD.

Models were adjusted for age, sex, race, smoking status, alcohol intake, physical activity, TDI, ALT, and AST

CVD: cardiovascular disease; ALT: alanine aminotransferase; AST: aspartate aminotransferase; TDI: Townsend deprivation index

**Figure S3. Forest Plots of Subgroup Associations of Cardiometabolic Burden with CVD and Death by Sex and Age**

Forest plots showing the HRs and 95% CIs for transitions from baseline to CVD, baseline to death, and CVD to death associated with cardiometabolic burden in subgroups defined by sex and age. The values are hazard ratios (HRs) and 95% confidence intervals (CIs) derived from multivariable multistate models.

Models were adjusted for age, sex, race, smoking status, alcohol intake, physical activity, TDI, ALT, and AST.

P for interaction was calculated to assess heterogeneity of associations across subgroups. The reference line at HR = 1 indicates no association.

CVD, cardiovascular disease; ALT, alanine aminotransferase; AST, aspartate aminotransferase; TDI, Townsend deprivation ;

**Figure S4. Time-stratified associations between cardiometabolic burden and risks of transitions from baseline to CVD, baseline to death, and CVD to death after excluding self-reported outcome events.**

Piecewise Cox models were adjusted for age, sex, race, smoking status, alcohol intake, physical activity, TDI, ALT, and AST. Time-specific hazard ratios (HRs) and 95% confidence intervals (CIs) for the associations of cardiometabolic burden with transitions from baseline to CVD, baseline to death, and CVD to death were estimated within predefined follow-up intervals (0–1 year, 1–3 years, and >3 years).

Nominal P values are reported alongside P for FDR-adjusted P values, which were calculated using the Benjamini–Hochberg

CVD: cardiovascular disease; ALT: alanine aminotransferase; AST: aspartate aminotransferase; TDI: Townsend deprivation index

**Figure S5. Time-stratified associations between cardiometabolic burden and risks of transitions from baseline to CVD, baseline to death, and CVD to death After Inclusion of Participants with Baseline Cancer.**

Piecewise Cox models were adjusted for age, sex, race, smoking status, alcohol intake, physical activity, TDI, ALT, and AST. Time-specific hazard ratios (HRs) and 95% confidence intervals (CIs) for the associations of cardiometabolic burden with transitions from baseline to CVD, baseline to death, and CVD to death were estimated within predefined follow-up intervals (0–1 year, 1–3 years, and >3 years).

Nominal P values are reported alongside P for FDR-adjusted P values, which were calculated using the Benjamini–Hochberg

CVD: cardiovascular disease; ALT: alanine aminotransferase; AST: aspartate aminotransferase; TDI: Townsend deprivation index

**Figure S6.Time-stratified associations between cardiometabolic burden and risks of transitions from baseline to CVD, baseline to death, and CVD to death with additional adjustment for diet score, CRP, and eGFR**.

Piecewise Cox models were adjusted for age, sex, race, smoking status, alcohol intake, physical activity, TDI, ALT, and AST. Time-specific hazard ratios (HRs) and 95% confidence intervals (CIs) for the associations of cardiometabolic burden with transitions from baseline to CVD, baseline to death, and CVD to death were estimated within predefined follow-up intervals (0–1 year, 1–3 years, and >3 years).

Nominal P values are reported alongside P for FDR-adjusted P values, which were calculated using the Benjamini–Hochberg

CVD: cardiovascular disease; ALT: alanine aminotransferase; AST: aspartate aminotransferase; TDI: Townsend deprivation index

**Figure S7. Time-stratified associations between cardiometabolic burden and risks of transitions from baseline to CVD, baseline to death, and CVD to death after redefining cardiovascular disease as a composite of coronary artery disease, stroke, and heart failure**.

Piecewise Cox models were adjusted for age, sex, race, smoking status, alcohol intake, physical activity, TDI, ALT, and AST. Time-specific hazard ratios (HRs) and 95% confidence intervals (CIs) for the associations of cardiometabolic burden with transitions from baseline to CVD, baseline to death, and CVD to death were estimated within predefined follow-up intervals (0–1 year, 1–3 years, and >3 years).

Nominal P values are reported alongside P for FDR-adjusted P values, which were calculated using the Benjamini–Hochberg

CVD, cardiovascular disease; ALT, alanine aminotransferase; AST, aspartate aminotransferase; TDI, Townsend deprivation index
